# Supplementary material for: CGMega: explainable graph neural network framework with attention mechanisms for cancer gene module dissection
Source: Nat Commun. 2024 Jul 17;15:5997. doi: 10.1038/s41467-024-50426-6 (PMC11252405; doi:10.1038/s41467-024-50426-6)
Supplement: Supplementary file 1 — Supplementary Information [file 41467_2024_50426_MOESM1_ESM.pdf]

CGMega: Explainable Graph Neural Network Framework with Attention Mechanisms for Cancer Gene Module Dissection

Supplemental Information

Supplementary Figures

Fig. S1 robustness of GNNExplainer in CGMega

Fig. S2 CGMega performance in cancer gene prediction task

Fig. S3 Methods comparison on input data with or without Hi-C

Fig. S4 Gene modules in breast cancer cell line

Fig. S5 Inhibitor combination experiments in breast cancer cell line

Fig. S6 Gene modules in AML patients

Supplementary Tables

Supplementary Table 1. The reported dataset in the main text (Dataset-1) and an external one (Dataset-2) for robustness evaluation on CGMega

Supplementary Table 2. CGMega performance across different breast cancer datasets

Supplementary Table 3. Candidate AML genes identified by CGMega in total

Supplementary Table 4. Candidate AML genes present in all AML patients

Supplementary Table 5. Candidate AML genes present in known AML gene modules for AML patient

Supplementary Table 6. Patient-specific candidate AML genes

Figure S1

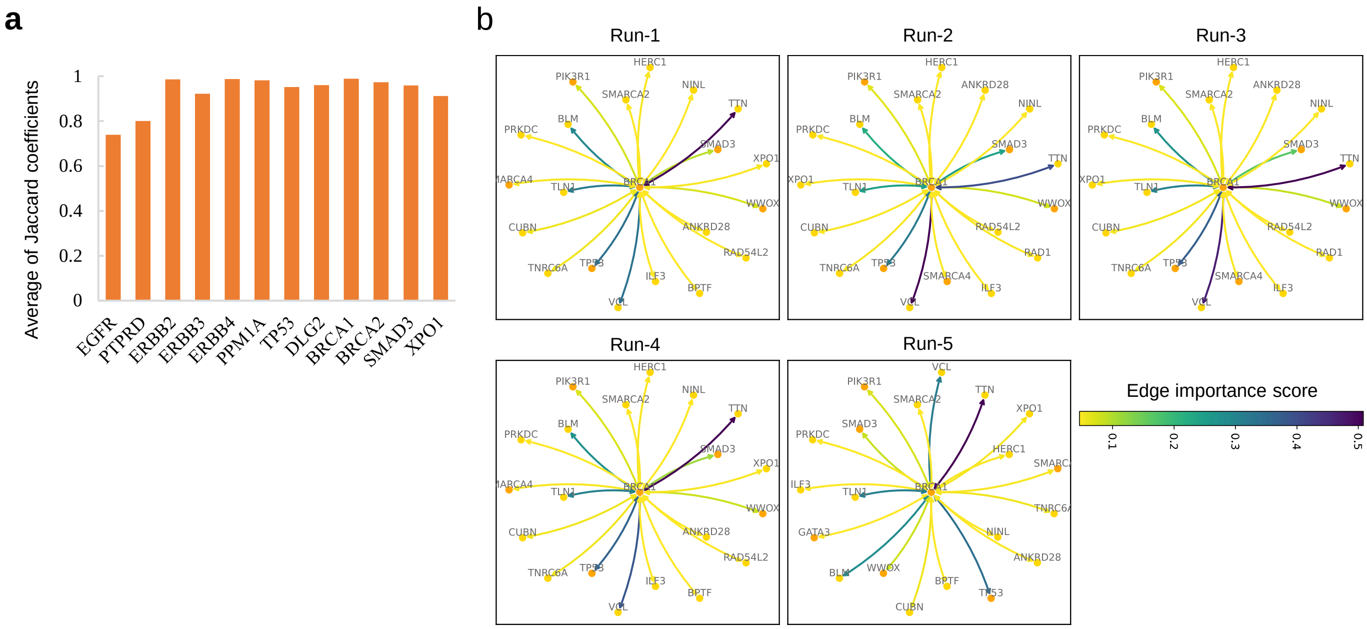

Fig. S1 robustness of GNNExplainer in CGMega. a We repeated GNNExplainer five times on 12 randomly selected cancer genes, and calculated the average Jaccard coefficients between any two repeats. **b** Taken BRCA1 gene module as an example to show the robustness of GNNExplainer interpretation. Source data are provided as a Source Data file.

Figure S2

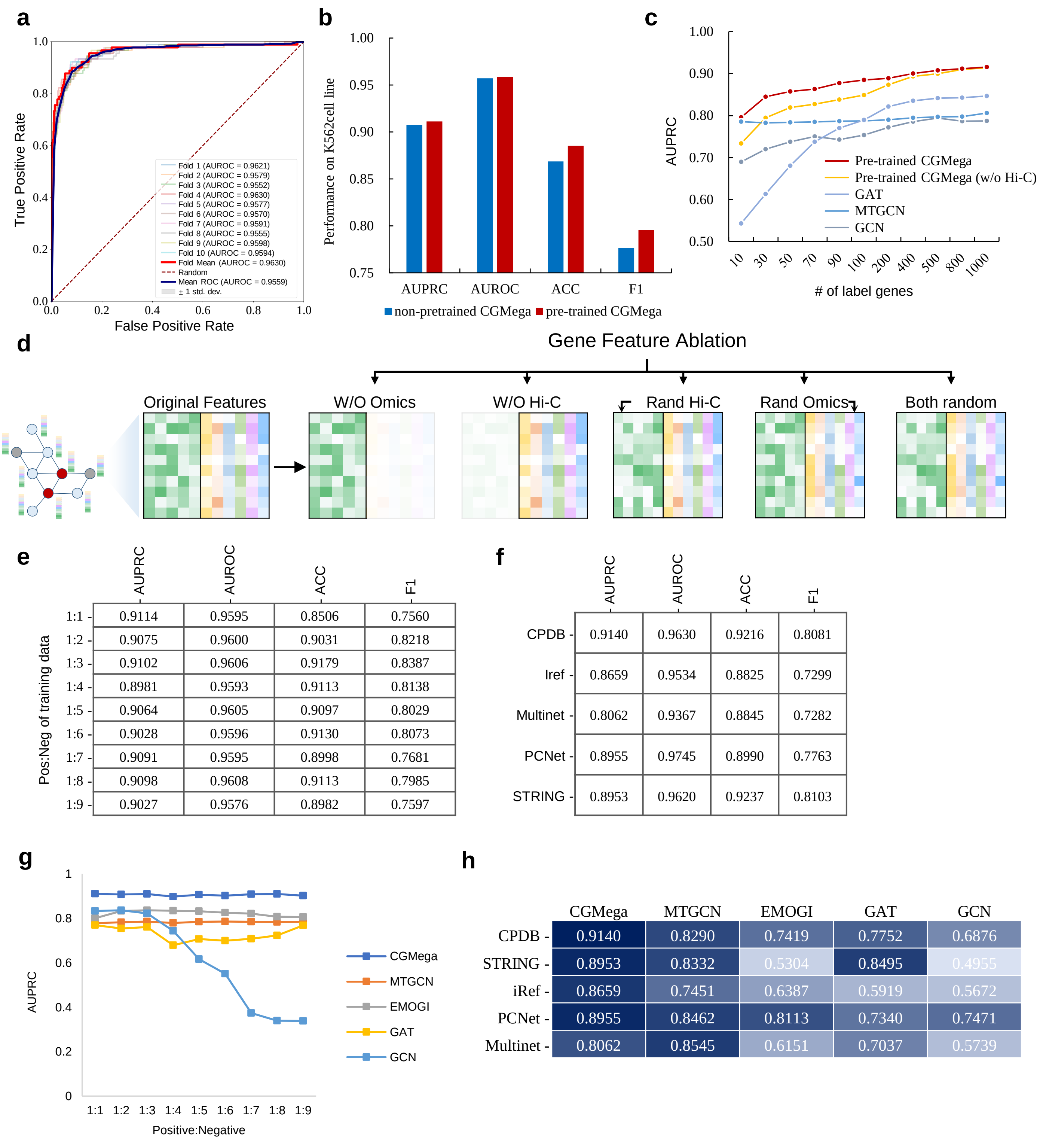

e

Pos:Neg of training data	AUPRC	AUROC	ACC	F1
1:1	0.9114	0.9595	0.8506	0.7560
1:2	0.9075	0.9600	0.9031	0.8218
1:3	0.9102	0.9606	0.9179	0.8387
1:4	0.8981	0.9593	0.9113	0.8138
1:5	0.9064	0.9605	0.9097	0.8029
1:6	0.9028	0.9596	0.9130	0.8073
1:7	0.9091	0.9595	0.8998	0.7681
1:8	0.9098	0.9608	0.9113	0.7985
1:9	0.9027	0.9576	0.8982	0.7597

f

	AUPRC	AUROC	ACC	F1
CPDB	0.9140	0.9630	0.9216	0.8081
Iref	0.8659	0.9534	0.8825	0.7299
Multinet	0.8062	0.9367	0.8845	0.7282
PCNet	0.8955	0.9745	0.8990	0.7763
STRING	0.8953	0.9620	0.9237	0.8103

h

	CGMega	MTGCN	EMOGI	GAT	GCN
CPDB	0.9140	0.8290	0.7419	0.7752	0.6876
STRING	0.8953	0.8332	0.5304	0.8495	0.4955
iRef	0.8659	0.7451	0.6387	0.5919	0.5672
PCNet	0.8955	0.8462	0.8113	0.7340	0.7471
Multinet	0.8062	0.8545	0.6151	0.7037	0.5739

Fig. S2 CGMega performance in cancer gene prediction task. a AUROC on breast cancer cell line MCF7. **b** Performance of non-pretrained and pre-trained CGMega on K562 cell line. **c** Methods comparison on datasets with different number of labeled genes. **d** Gene feature ablation illustration. Five types of feature ablation inputs were generated, including the removal of omics feature and Hi-C feature and random generation of Hi-C feature, omics feature, and both omics and Hi-C features. **e** Performance of CGMega on datasets with different positive to negative ratios. **f** Performance of CGMega on different PPI databases. **g** AUROCs of different methods on datasets with different positive to negative ratios. **h** AUPRC of different methods on different PPI datasets. Source data are provided as a Source Data file.

Figure S3

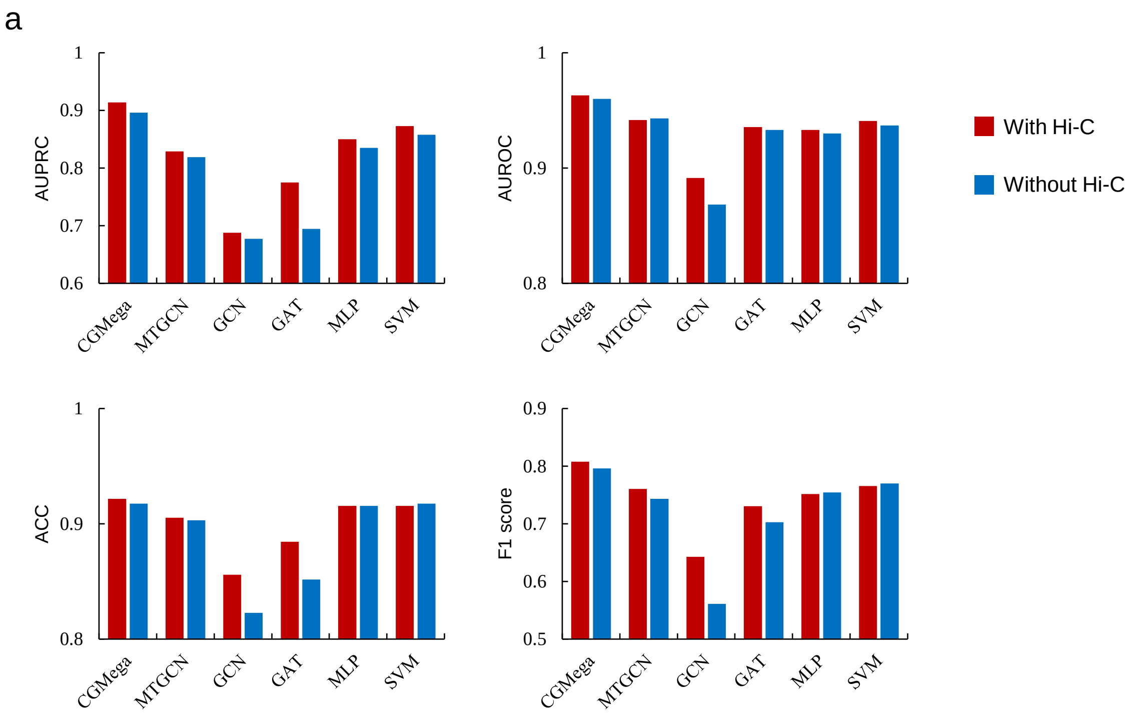

Fig. S3 a Methods comparison on input data with or without Hi-C. Source data are provided as a Source Data file.

Figure S4

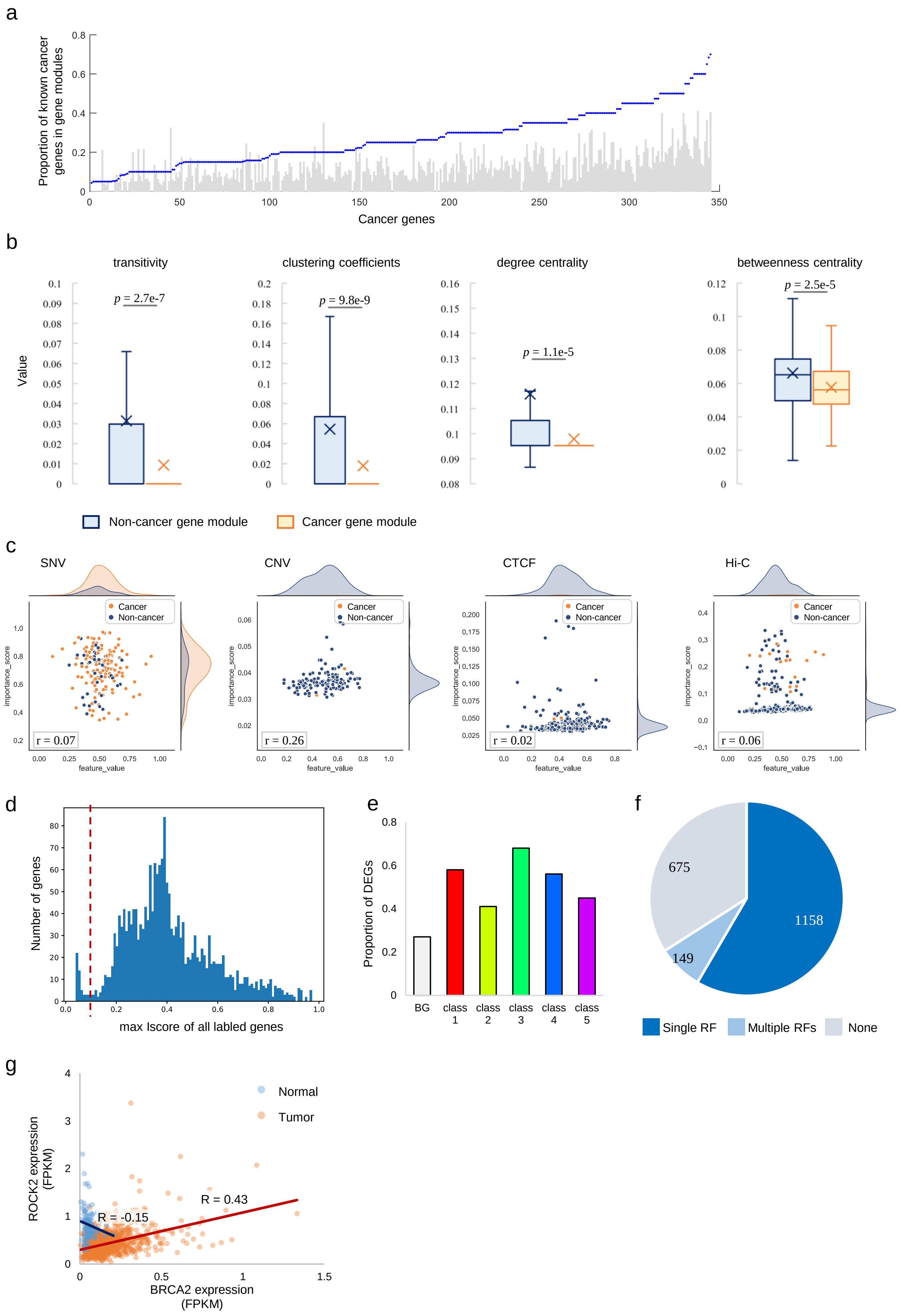

a
Proportion of known cancer genes in gene modules
Cancer genes
b
transitivity
clustering coefficients
degree centrality
betweenness centrality
Value
p = 2.7e-7
p = 9.8e-9
p = 1.1e-5
p = 2.5e-5
Non-cancer gene module
Cancer gene module
c
SNV
CNV
CTCF
Hi-C
Cancer
Non-cancer
importance_score
feature_value
r = 0.07
r = 0.26
r = 0.02
r = 0.06
d
Number of genes
max lscore of all labled genes
e
Proportion of DEGs
BG
class 1
class 2
class 3
class 4
class 5
f
675
1158
149
Single RF
Multiple RFs
None
g
ROCK2 expression (FPKM)
BRCA2 expression (FPKM)
Normal
Tumor
R = 0.43
R = -0.15

Fig. S4 Gene modules in breast cancer cell line. a For each cancer gene module, the proportion of known cancer genes was calculated (blue dots). Random gene modules with the same number of genes was shuffled 100 times, and the average proportion of known cancer genes in the module was calculated (gray bars). **b** Graphical metrics of gene modules, p values were calculated by paired t-test. **c** Scatter plots show the distributions of raw feature inputs and importance scores calculated by GNNExplainer. **d** Distribution of the highest importance score of genes showed that only dozens of genes with importance score lower than 0.1 (red line). **e** Proportion of DEGs in each class, data were from TCGA project, colors of each class corresponded to Fig. 4b**. f** Distribution of genes with different numbers of RFs. **g** scatter plot showing the co-expression of BRCA2 and ROCK2 in normal (blue dots) and tumor (orange dots), expression data were obtained from TCGA project. Source data are provided as a Source Data file.

Figure S5

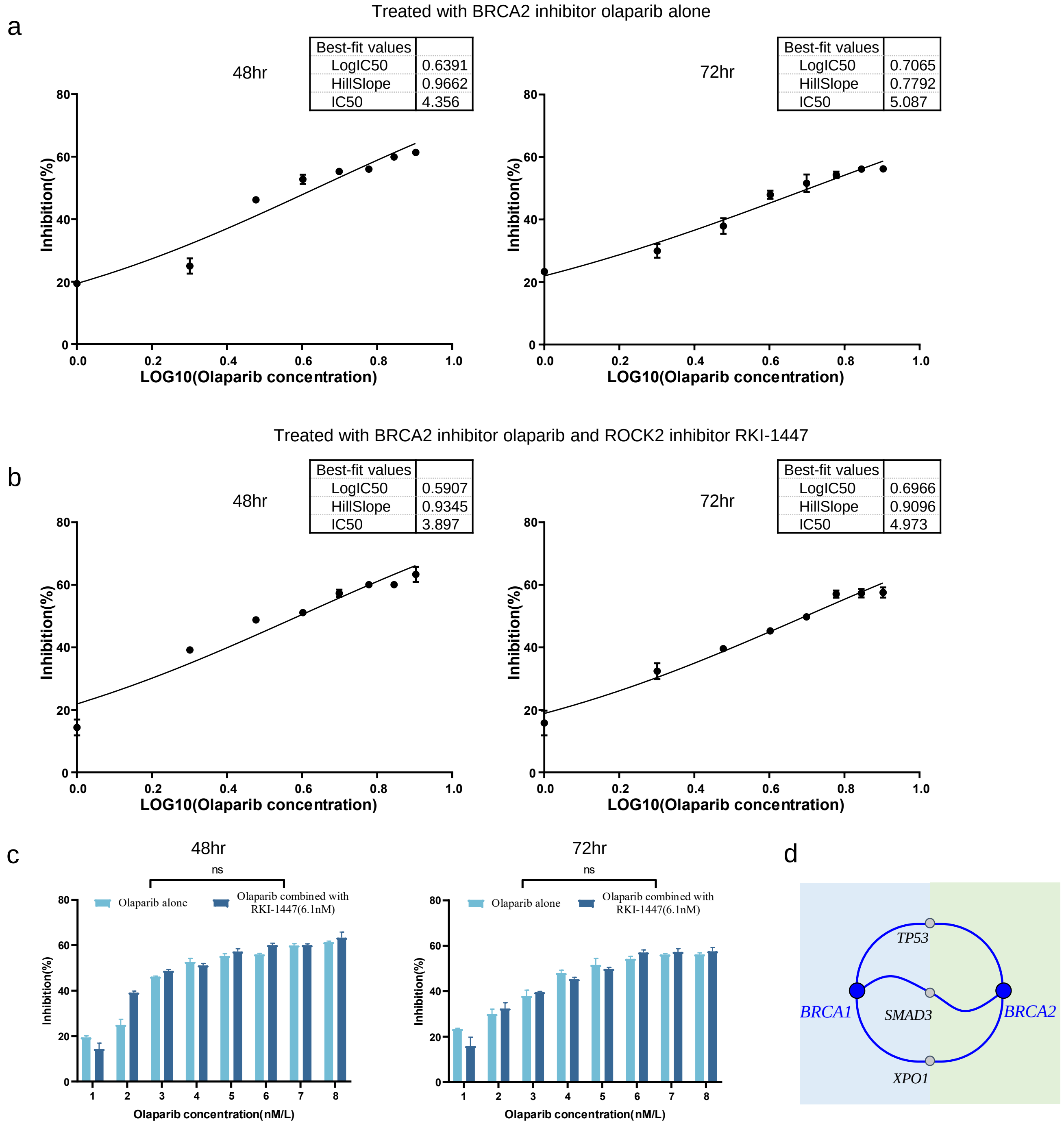

Fig. S5 Inhibitor combination experiments in breast cancer cell line. a,b Half maximal inhibitory concentration (IC_{50}) value of olaparib treatment (**a**) and olaparib/RKI-1447 combination treatment (**b**) after 48 hr and 72 hr. **c** Paired *t*-test analysis based on the inhibition rates between olaparib alone and olaparib/RKI-1447 combination after 48 hr treatment (left) and 72 hr treatment (right). Paired *t*-test was used to analyze the two groups. **d** *BRCA1* and *BRCA2* gene modules formed a high-order gene module through three shared genes including *TP53*, *SMAD3*, and *XPO1*. Source data are provided as a Source Data file.

Figure S6

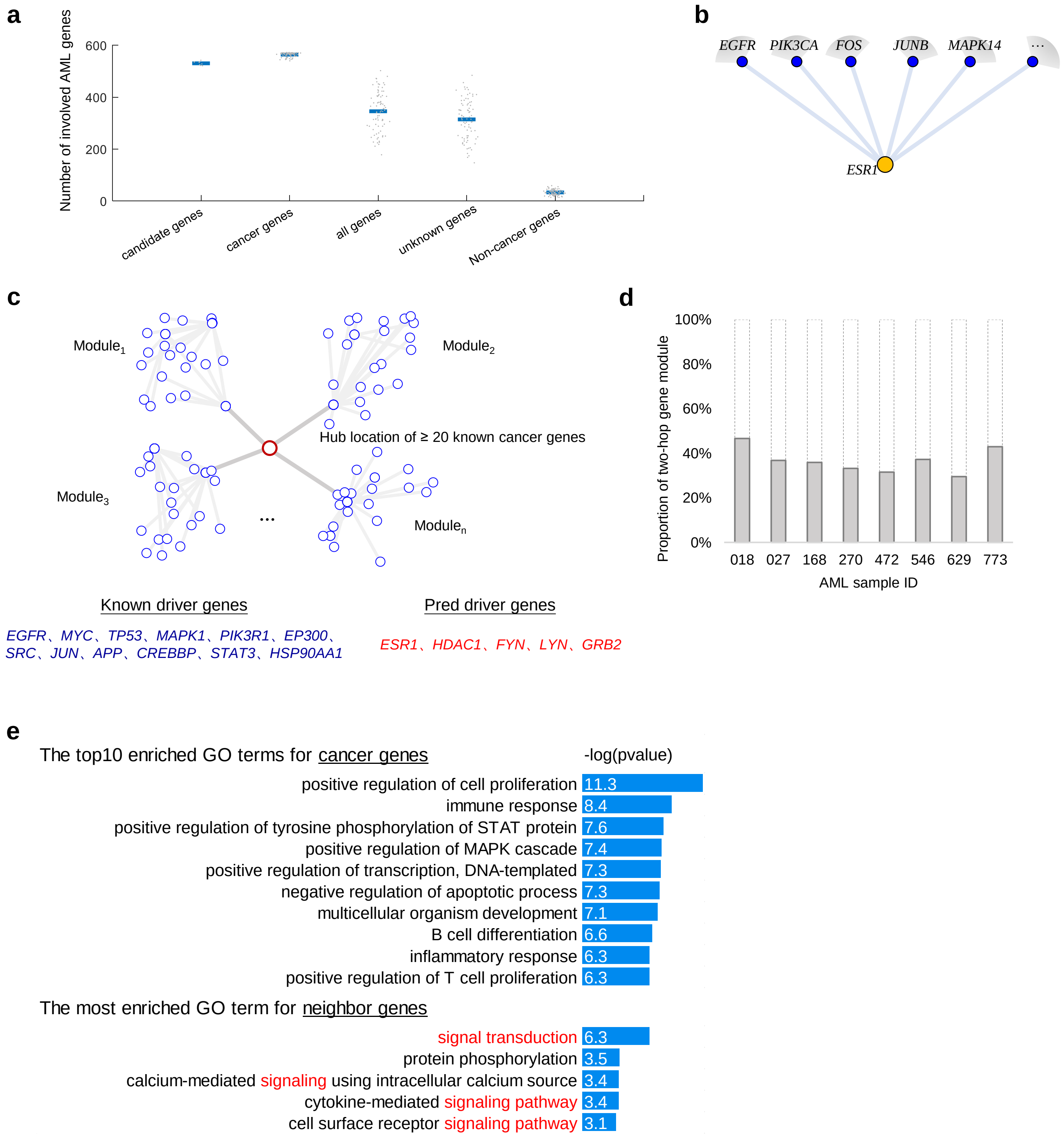

Fig. S6 Gene modules in AML patients. a Number of AML genes present in the module of candidate gene set and other gene set (10 times randomly sampled with the same size). Each dot in the first column (candidate genes) or from the second to the fifth column (other genes) indicates the scale of involved AML genes in the corresponding gene set. **b** Illustration of *ESR1* in various cancer gene modules. Gray background indicates other parts of gene modules expect *ESR1*. **c** Illustration showing the 12 known AML genes and 5 candidate AML genes in all eight AML samples. **d** Proportion of two-hop patterns in AML gene modules for each AML sample. **e** GO analysis for genes in 142 neighbor-cancer gene pairs. For cancer genes, we shows the top 10 GO terms. For neighbor genes, only 5 GO term were significant (P value < 1e-3). GO analysis was conducted using DAVID. Source data are provided as a Source Data file.

Supplementary Table 1. The reported dataset in the main text (Dataset-1) and an external one (Dataset-2) for robustness evaluation on CGMega

	**Dataset-1**	**Dataset-2**
**H3K4me3**	ENCODE project<br>ENCFF145CCI / ENCFF268RXB	ENCODE project<br>ENCFF078BWS / ENCFF251QQR
**H3K27ac**	ENCODE project<br>ENCFF340KSH / ENCFF491LQY	ENCODE project<br>ENCFF054VCV / ENCFF071XTD
**ATAC-seq**	ENCODE project<br>ENCFF821OEF	ENCODE project<br>ENCFF976UNK
**CTCF**	ENCODE project<br>ENCFF138LHE / ENCFF163JHE / ENCFF198YOJ	ENCODE project<br>ENCFF157EYO / ENCFF237BZX / ENCFF915BMD
**CNV**	TCGA project<br>530 Breast cancer samples	TCGA project<br>106 randomly selected Breast cancer samples
**SNV**	TCGA project<br>4 Breast cancer samples	TCGA project<br>2 randomly selected Breast cancer samples
**Hi-C**	Genome Biology, 2015<br>GSE66733	Nucleic Acids Research, 2021<br>GSE182306

Supplementary Table 2. CGMega performance across different breast cancer datasets

**CGMega performance across different breast cancer datasets**				
	**AUPRC**	**AUROC**	**ACC**	**F1**
Dataset-1 (Reported in paper)	0.9140	0.9630	0.9216	0.8081
Dataset-2	0.9072	0.9627	0.9320	0.8272

Supplementary Table 3. Candidate AML genes identified by CGMega in total

MATN2	SMC3	UBC	PRKACA	FOXO1	BSN	FER	DCC	CEP120	EIF4E
KRT8	CASK	PRKD1	TNKS2	DYRK1A	EPB41L3	PRKCE	PPP2R2B	KAT2B	SMAD2
NRG1	RARB	VTN	RPS6KA2	CTNND2	LARP7	NFATC2	TNK2	CUL3	ANK2
HNRNPM	PDPK1	AKAP12	ELN	HNRNPU	KAT5	NTRK2	FAF1	SNW1	TANC1
SHANK1	TRPC3	CGN	MAP3K5	PRKG1	E2F3	KHDRBS2	HCK	STAT1	AMOT
BICD1	GSN	CTBP1	EXOC1	SF1	PXN	ITPR1	SMAD3	SMARCE1	SHC1
DYNC1I1	MCM3	POR	SPTAN1	ANK1	IFT74	SMARCC1	GRIN2B	ALMS1	POU2F1
HIPK2	CAMK2D	TP63	SNX9	KCNIP4	PDE4D	MAPK10	AP2M1	YWHAG	RGS7
CDC16	ACTN4	EZR	SREBF2	ESRRA	IQGAP1	ACTA1	FLNC	CDC5L	KCND2
MYO6	AP2S1	DLGAP2	CD2AP	ANXA7	THRA	SUMO1	ITGB5	NCOR2	MEF2A
HDAC6	RTN4	KCNB2	HNRNPD	NUDC	RBPJ	XRCC6	TSHR	PLA2G4A	PPM1A
GAN	TNKS	NTM	ATXN1	FBN1	TEAD2	MEF2C	DNM3	DAB2	DAG1
SIRT6	CHCHD3	DLGAP3	GRB2	HOMER1	PRKDC	SMAD1	RPA1	PTPRA	DTNBP1
HDAC7	CDC25C	PRKAR1A	ECT2	PTK2	DLG1	AMPH	PABPC1	AP2B1	CEP128
LATS2	KIF13B	PTPRB	MET	FYN	FOXJ2	S100A10	GRB10	CPSF6	HNRNPA1
NIN	CAMK2B	MYH9	TUBA1B	LYN	YWHAH	CARM1	PTPRK	EXOC2	PLEKHA7
MMP10	FAM20C	PTBP3	NCOA2	CALM3	CCT7	ADRB2	GRID2	PSEN1	GNA12
HDAC5	SMARCA4	LEF1	ETS1	PHGDH	TGFBR2	IMMP2L	KIAA0753	ACTA2	XRCC5
TYK2	SP1	TAB2	LMNA	VAV3	FBN2	SGK1	GAK	NME7	FLNA
EPHA4	NCOR1	PPP3CA	PPP1CB	EGLN3	SUMO2	NCOA3	RPA2	MAPKAP1	GJA1
GRM1	MACF1	KCNB1	GRIK2	TUBA1C	GNA13	CACNA1D	HMGB1	DLG2	ESR1
FBXW11	PRKAR2A	DISC1	TLN1	NEDD4	CEP170	CHUK	PDGFRA	NR2C2	RNF41
VDAC1	BTRC	ANK3	PLCG2	HDAC1	GAB1	CNTRL	SQSTM1	OPTN	ACTN1
CEP290	VCP	CDC20	EPHA2	BCAR1	YBX1	GRM7	CDK9	PHLDB2	CSNK1A1
ROBO1	TPM3	TNIK	NLGN1	AURKA	GNAO1	PRKCZ	BARD1	YWHAB	AKAP5
BLM	MLH1	MED13	PICK1	SH3GL3	DOCK1	MAPT	UBXN7	GRIK4	PPP2R1A
GRIA2	IRS1	PTPRR	APPL1	PRKCA	PTPRJ	ODF2	PRKACB	MED23	POU6F2
ARNT	COIL	DLC1	NINL	MAPK9	PCM1	EXOC6	CDC14A	SRPK2	NRXN1
ARRB1	ESR2	FUS	HMGA1	MYO19	TRAF6	RAD50	PPARD	YWHAZ	MTOR
MAD1L1	CEP135	HNRNPF	CUL1	ITSN1	EXOC4	LSAMP	KIF20A	NEB	RPS6KA3
MAGI1	MSN	BBS7	MED4	TUBB	NKX2-1	TERF1	ENO1	MAX	HTT
PPP3R1	WWTR1	CTNND1	ATR	PRKCI	RNF2	RXRG	STAU1	DHX15	PAK2
GNAI2	YES1	ACTN2	PLCG1	PIK3R3	AFAP1	ANLN	ROBO2	GRM3	PIBF1
CSNK2A1	TBP	MAML1	GNAI3	LRP2	EIF3E	TRAF3IP1	MAP3K3	CNTN1	ITGB1
VAV1	RELA	GRIK5	PTPRZ1	NR2F1	EXOC7	ABI2	RYR2	PPP2R5C	HDAC3
CAMK2G	TUBB3	TJP1	RPA3	PAFAH1B1	GRIA1	IKBKB	PRKAR2B	RBBP4	DLG3
PTPRG	LIMA1	PPARA	ACTB	SPTBN1	DNM1	RYR1	ABI1	TNS1	PPM1G
HSP90B1	SVIL	RHOA	CAMK2A	HSP90AB1	ERC1	KCNA2	SIRT1	SPICE1	PAK1
HOOK1	CCT4	TRIM28	THRB	RANGAP1	CENPJ	AHR	IGFBP5	TRIM24	GNAQ
CDH5	CSNK2A2	MAGI2	PLK1	DDX5	TSC2	ATXN2	TTN	OFD1	MYO5C
NETO1	ADCY5	TES	SMARCB1	NTRK3	BIN1	TRIM25	SYNCRIP	TOP1	FOXP1

DHX9	TEAD1	TFAP2A	NCL	PRKCG	WWP2	PIAS1	PDE1A	MED17	TPM2
HSPA6	ENAH	CEP350	SCN2B	GNB1	RPTOR	PSMD2	DDB1	KMT2A	GNB5
FRS3	REL	KAT2A	CDH18	CAV3	UBE3A	CCND3	DVL2	AMOTL1	KIF5A
DCN	RAPH1	CEP63	TLE1	HSPG2	GNB2	KIF11	MYO18A	FANCD2	PSEN2
SENP3	PRKAR1B	TAF5	PRKACG	KIFAP3	DRD2	HDAC4	VAPB	TAF1	PTGER3
LIMK1	EFNA5	DYNC1I2	EPHA8	CALM1	DLG4	RIMS1	NUMB	WWP1	SRSF1
CEP104	LIN7A	COL4A3	TPM1	MED27	AR	CEP85	NEK4	TAB1	UBE2E2
MORF4L2	KIF23	VRK1	GRIA4	RBM47	RYR3	GRIN1	SYNE2	LTBP1	MAD2L2
RFX3	ITCH	PIP5K1C	PIAS2	AP1G1	OS9	SORBS1	NEBL	LRRC49	INSR
SCRIB	DPF2	NTRK1	SMARCA5	RAP1A	RPRD1B	PPP1CC	FKBP1B	ADAMTSL1	RAE1
STON2	MYH10	GOPC	PRKG2	MED24	CANX	CDK8	RPH3A	CTBP2	TIAM1
MNAT1	RSU1	BICD2	RBM14	CAMSAP1	WWOX	GABARAP	CACYBP	BLK	MSH3
BBS2	MORF4L1	ELMO1	AKAP13	PPP2R2A	MYO5B	SUGT1	ANKS1B	NFE2L2	RUVBL1
IFT88	SIAH1	CDC27	CIT	RFC3	EPS15	MYOM2	MYO5A	RCC1	ACTC1
ATRX	MIB1	GTF2B	SIN3A	GLI3	IQCB1	SMARCD1	MAP2	NR1H3	CAPZA2
SATB1	WDR5	HAUS3	KLHL22	TRIM5	ITGB3	SUPT5H	VWCE	BRD4	SOX2
USH1C	KIF14	SMARCA2	PRELP	EXOC6B	RPS6KB1	GRIN2A	MTUS2	NFYA	NR1H4
VCL	MCM5	EFEMP1	RDX	GRB7	MGAT4C	NUP98	SYT1	EEA1	GRIP1
PSMA1	RAD51	CACNA1B	CDH23	RNGTT	CD81	ATF2	MARK4	NONO	FHL2
PLD1	AP2A1	RASGRF1	RAB3A	GRIN3A	MYO7A	KCNMA1	MTA2	NCK2	CACNB3
ABLIM1	WASF1	KPNB1	RBBP5	NF2	LZTS2	CCDC14	CHD3	RBM39	TBC1D31
ANKRD28	HAX1	DCLK1	CYLD	C2CD3	CIC	ILF3	MYCBP2	CEP192	GSK3B
PKP4	DYNLL1	TCF7L2	PDLIM7	GSK3A	WRAP73	APOB	TRIM27	LMNB1	NDC80
TP53BP1	PKM	SLIT2	CBLB	CEP152	CAPZA1	KIF3A	CORO1C	FLNB	PPP1R12A
GAB2	LOX	ATF7IP	NEDD8	VASP	PUF60	NFATC1	DDX21	ERC2	MED1
CIITA	VDAC2	DHPS	TCF12	CDH2	RUNX2	FBLN2	TARDBP	AKAP6	IRF2
BMI1	DPF3	TMOD1	OCRL	INS	CTTN	KCTD10	HNRNPK	UBB	RAC1
RPS3A	TUBB2B	TADA2A	FRAS1	CNTNAP2	LRRK2	SYNPO	AGRN	FGFR4	NOS1
DACH1	STAG1	TNRC6B	SMURF1	ATF4	YWHAQ	STAT5B	RPS6KA5	PLCB1	OPRD1
HNRNPA2B1	PIK3CG	TCHP	VAPA	SNRNP70	SH3GL2	MATR3	UBE2I	SNTB1	OPCML
GABRG2	APC	VAV2	DMD	UBAP2L	MCRS1	RSPRY1	MAPRE1	SERPINE1	SMAD4
PPP2CA	NBN	HMGA2	ARRB2	SRPK1	COL1A1	EPAS1	ANXA1	CDC37	TCF4
LASP1	CACNA1C	HSPA2	DDX1	NSF	PRKCD	FKBP1A	POC5	STUB1	MRPS22
ARHGAP32	NDEL1	LNX1	MYO1C	PLB1	NCF1	UBE2L3	STK11	HTRA1	EVC2
AGO2	DAPK1	KRT18	ADAM17	CDH7	UNC13B	GNG2	PTPRD	CACNB1	SMURF2
ADAM10	CDH8	NCOA1	SPTBN2	DPYSL2	NUP93	GPHN	DDB2	ELK1	CEP89
KCND3	CAPN1	GRID1	CDC73	STX1A	NFX1	KCNE1	GIPC1	FOXC1	GNA11
GABRB3	PSMC5	CTNNA3	CRK	XPO7	BBS4	ELAVL1	ILF2	MYOM1	ASH2L
SPOP	MED19	TBL1X	CNTROB	NEK6	TUBB4B	CBX5	CAMK1	OCLN	TRDN
PTPN7	FIP1L1	KCNQ5	MFAP5	KLHL13	EXOC3	RAP1B	CFL1	PIP5K1A	CALU
BBS5	CUL7	TXN	GABARAPL2	BBS1	GABRB2	MCC	GRIN2D	NRCAM	GNG4
NAV1	KIF2C	SYNGAP1	PIK3CB	STXBP1	TAF12	GNG12	CDC25B	GTSE1	NR1H2
FOXA1	LRWD1	EGFL7	TNRC6A	PCLO	HNRNPDL	CHD4	FMR1	MYH1	RAD18

PDE1C	GRID2IP	REPS1	MED16	MAP1A	ESRRG	AKAP9	GOLGA2	ORC2	PLK4
GABRA1	RAB11A	CUL2	NUFIP1	IFI16	PSMC2	RNF4	PTPN13	SYBU	L3MBTL1
MAPK7	PDK1	SSBP1	PARD6B	TGFB2	CBX3	ZC3H18	RPGRIP1L	CEP250	NR2F2
FXR2	MSX1	CDC42	TRIM37	TUBG1	SDHB	EEF1D	TGFBR1	TNFAIP3	CHRNA7
FXR1	CSPP1	MPDZ	PLD2	PIAS4	OBSL1	PSPC1	PIK3R2	BYSL	ALK
SMAD9	CDH12	CLTCL1	NR1I2	GRIA3	STX3	KANSL1	TLE3	LRRC4C	MCM2
PRKCB	CADPS	DAP3	DUSP6	MYPN	ANKRD11	PLEC	MTUS1	GNG7	HDAC2
TSC1	ZFHX3	EXOSC10	RXRB	GATAD2B	VDAC3	TJP2	AVIL	FBP2	IGF2BP2
CACNB2	SRSF5	ATP2B2	EPB41	AP2A2	PPP3CB	DOCK2	UBR5	ABL2	RACGAP1
PKP2	ARNT2	YAP1	PTPN12	SH3PXD2A	PARVA	BMPR1A	ROCK1	KCNC1	KEAP1
MID1	LDLR	FOXE1	PALLD	NOTCH2	SH2B3	SMARCAD1	ACTR1A	NDUFS2	GABBR1
IGF2BP1	GPS2	BMP1	CNTNAP1	SNRNP200	EXOC8	MAP2K6	EHD1	ATG5	EEF2
TTC8	DCTN2	CLASP1	TXNRD1	YY1	ARNTL	TNC	MGMT	LRRC59	ASPH
ZW10	NECAP2	SNRPC	FZD1	SF3A3	IKZF3	USF1	IGF2R	ATXN3	SNRPN
SH3KBP1	RYK	PYHIN1	BBS12	TADA1	DST	FLOT1	CCT3	FAF2	MRPS31
NELL1	NANOG	RC3H1	LONP1	RICTOR	PRC1	CLU	CCDC6	SNAP23	NOP56
TPT1	NEO1	RABGAP1	HELZ	UPF1	GABBR2	PARD6A	SEC23A	RPS27A	RPS6
PRPF40A	UVRAG	DPP10	NADK	LOXL1	PPP2CB	NFYB	POLD1	PRPF3	MAGOH
ITPR3	UGT1A6	DSP	ALB	NUP214	SGCG	DBN1	KSR1	TRPC1	LARS2
PARD3	FOXO3	TRAF3	STIL	EEF1A1	NRP1	AP1B1	EPHB2	RNF111	MYBPC2
HNRNPC	RPL4	ANAPC10	EGLN2	LDHA	NELL2	SUPT20H	SGIP1	USP22	EPN1
PPARGC1B	KCNE2	PPP2R2C	MYO10	HDAC8	PDE4B	BUB1	LIMK2	MYBL2	ECM1
KCNJ4	ERBB3	RAD23A	BBS10	BMX	MARK2	GRM5	PRKCH	MAP2K4	ANAPC7
MMP2	FZR1	MAP2K5	TCP1	LMO4	HOMER2	MAD2L1	PTPN1	RBBP7	HADHB
AURKB	TGFBI	PLCE1	TFAP2C	RBL1	HK1	PCDH15	SASS6	MYBPC1	CACNB4
FSCN1	FAM161A	SSRP1	UGT1A10	ACLY	MDC1	CLUAP1	CAMK4	MYL2	ADCY2
NR1I3	KCNQ1	SUV39H1	RAPGEF2	CCKBR	ATF1	SART1	SMAD5	CSN1S1	MSX2
SNAPIN	LRRC4	BAIAP2	EML1	HOOK2	CHRM3	ACAN	COPS6	MYLK	PSAT1
GOLM1	SLC25A5	CDC42BPA	AP3B1	ARHGEF2	NR0B2	SPTA1	SPAG5	CCT6A	TNNT1
INA	UGT1A4	NLGN2	SRI	WASL	AHNAK	VWA8	DGKI	GRB14	CEP97
CSGALNACT2	CRIPT	HIRA	HLA-B	RAB7A	SF3B3	NEDD9	EFTUD2	COL8A2	KIFC3
CHSY3	HERC2	TPX2	TERF2IP	ACVR1	FBF1	TRPC4	MAGI3	LAMC1	CNOT1
LTBP4	CACNA1A	COL5A1	KCNF1	RSL1D1	TUBA1A	NSMCE2	PRPS1	PARVB	CEP70
ACTG1	KCNQ3	PARP2	PRPF8	FERMT2	CEP57	USP8	NDE1	TIMP3	TERF2
TRPC6	UGT1A3	PFDN1	PSMB4	KIF1B	COL13A1	PTPRM	SUPT7L	PGK1	ATN1
KIF5B	AUH	CAPZB	SNX5	SYNE1	COL4A4	ATF3	PAPSS1	FEZ1	JUND
UGT1A9	SLC25A12	ORC3	PHLPP1	KIF1A	RIPK1	PTPN4	ATAD3A	WAS	BCL6
PRMT1	PIH1D1	NR4A2	WFS1	ITGAV	ACTL6A	MUTYH	NLRP3	CACNA2D1	WRN
APBB1	MAPK8IP1	RASAL2	SIRT7	SUPT3H	DYNC1LI2	GRIN3B	GRIK1	COL1A2	ACTR2
UGDH	FES	SGCZ	CBX1	HNRNPA0	ATP1A1	SNX2	PTPRO	WDR62	TRAF2
BUB1B	RBMX	SF3A1	ZNF423	CALD1	KDM1A	KAT6B	COPS2	CDT1	TBK1
UGT1A1	RAPGEF4	MED26	RNF31	TAF4	CTNNA2	STAT2	CACNA1S	SPRY2	EFNB2
MAPRE3	FANCC	PGD	ZC3H15	LPP	FBLN5	ERCC1	COPB1	FZD4	GNB4

OGT	LINGO1	APBB2	RIMS2	CNN1	NEDD4L	PAICS	TUBB2A	HIP1	BBS9
SND1	TRIM21	UBE2A	SLC25A4	SETDB1	CLIP2	DNAJB5	SEC13	TAF3	NOTCH3
PLCB3	DDX6	ASIC3	HNRNPH1	FBLN1	UQCRB	FRS2	RPLP0	COL4A6	NUDT21
CEP72	FAM110B	FKBP5	CNKSR2	TBXA2R	IPO5	TTF2	FNDC3B	HAUS6	ZYX
MYH7	POLR2E	KCNIP1	AXIN1	EIF4G1	STK3	GTF2F2	RPS8	LDHB	PCOLCE
TTLL5	SNTG2	ANKRD26	SOX5	LAMA1	PLTP	NDUFV1	CKAP4	NOTCH4	ADD1
CDK5RAP2	PTPRS	E2F6	KLC1	LAMP1	KIF7	SOD1	CDC25A	FBXW8	AEBP2
POLR2D	OLFM2	HTR2A	HNRNPA3	MDH2	HSD17B10	SF3B2	KCNG4	GTF2A1	HAUS8
RMND1	CADM1	ERCC4	CDH9	ATF6	CBLN1	C1QBP	RAB27B	U2AF2	AKAP11
MICAL3	IRS4	RAB11FIP2	DNAH10	CHEK1	SLC25A11	SLC25A3	PACRG	DNAJA1	GATA4
KCNQ2	PTPRF	ARHGEF1	SMARCD3	UBASH3B	TIMM44	NDUFA9	DMAP1	RGS2	DYNC1LI1
COL4A5	HTR1A	SRRM1	PSMD4	TRIP4	SDC2	MRPS27	STX8	SUCLA2	SUPT16H
SMAD6	SFXN1	TRAF1	FHL3	ATG16L1	NID1	ARPC2	PMS2	PLXNA1	MTTP
RAB11FIP3	PDCD6IP	TDG	PDHA1	POU5F1	CYP3A4	PKD2	SLC25A41	NEGR1	BRD8
SH3GLB1	EPHB1	MED8	NCKAP1	GAD2	LIN7B	MYBPC3	ALG13	FGFR3	ACVR2A
HYOU1	PPIB	SH3GL1	DYNC1H1	ELMO2	XRCC4	MRPS9	C7	MED7	KPNA2
METTL3	MAP4	MAP2K7	ANAPC4	RPA4	SLC1A1	DES	TNIP1	PAXIP1	FBXO5
HOXA10	AGO1	PRDX5	KPNA3	KCNG3	TRIM63	SNAP91	PDIA6	COG6	HSPA1L
JPH3	MED30	BMPR1B	FOXP2	UQCRC1	MAN2A1	SKAP1	EPHB3	TADA3	CAMSAP2
PFDN6	PSMD1	RPL12	TUBB8	CBY1	SYN1	SCO1	TUBA4A	SCARA3	SUCLG2
PRKAA1	TAF6	ITPR2	CHN1	UBE2T	SRPRB	SOSTDC1	KCNJ6	SHC4	CASP7
CCDC8	DRD4	LIG4	EIF3B	CRMP1	KLC3	HAUS2	CACNG2	BCAN	CREB5
NR2F6	CCT8	MED14	RAB27A	MTMR2	MBD2	MTNR1A	PSMD14	RANBP2	ANAPC2
EIF3A	L1CAM	LAMB1	G3BP1	SNX6	HGS	SORBS2	LRPPRC	S100A6	FOXN3
LCP2	OBSCN	EPHA3	DNAH14	GLRB	UQCRC2	PLOD1	HAUS4	POLA1	UBE2E1
JARID2	IVNS1ABP	SORT1	LRFN1	CCNT1	PRKAA2	ARIH1	REST	CD247	SGCA
HADHA	MYBPH	HK2	AK2	SKI	POLR2C	GRM8	PSMB9	RUVBL2	PDIA3
TRIM55	RPH3AL	PRRC2A	ABCA1	OMA1	FOXG1	SMC5	VCAM1	IFT57	AHI1
CEP164	LAMC2	RPN1	ATRIP	HABP2	CUBN	EIF4B	TNNI1	CENPE	SRGAP3
UNC13A	CCT2	STARD13	BAZ1A	ATP6V1A	SRGAP2	UGT1A8	NLGN3	PBXIP1	NUPR1
E2F4	ORC4	LDLRAP1	CCNA2	BTC	WDHD1	PHF20	ANAPC5	TNR	IRF9
PDHB	RPS24	EPHA1	IL1R2	OPA1	UBR1	ITGB1BP2	ARF1	MTNR1B	C6
ASAP2	MID2	TNNI3	PLAUR	MRPL11	ARPC4	FBL	MBD3	FGL1	FREM2
LGALS3	CTTNBP2NL	DERL1	SNTG1	CYB5A	SOD2	PSMD11	FZD7	IRF1	TAF6L
SP100	EHMT2	ZC3HAV1	GABRA5	CKAP5	TSNAX	BSG	PDLIM5	KIF9	SKAP2
TAF9	SF3A2	MFAP4	IL1RAP	CHRM5	GTF2I	SPTB	RAB11FIP4	NAA15	LOXL3
LMO7	KCTD16	LATS1	DEPDC1B	COL8A1	PDE1B	TGFA	EPHA7	ERG	BCL2L14
CDC14B	STIP1	POLE	DCTN4	SHROOM3	NUP133	FCGR2B	EFNB1	CBFA2T2	TRIP10
GPC1	TAP1	F13A1	SCN5A	SACM1L	LDB2	NEDD1	PBX2	HSPE1	CNOT7
HDAC11	VMP1	NUBPL	SNTA1	SAE1	MED18	TOMM20	HLA-DRA	RBM25	CHCHD10
SPOCK3	GBE1	ACTR3	SNRPD3	MYO1D	RPE	KIF2A	APBB1IP	DNMBP	TTK
EPHA5	SCARB2	MRPS21	GOLGA5	DDX17	WEE1	ABCD3	STAMBP	NDUFA4	XRN2
NLK	LAMB2	FARSB	TOMM22	EPS15L1	FRMD6	SMG6	UBE2E3	NEXN	SAP130

TUBGCP4	BCAS3	SATB2	TPM4	EFEMP2	HTRA2	MAFB	PFKP	MYH2	EMD
GRM2	TIE1	PFKFB3	MAP2K3	IRF3	IKBKE	LRPAP1	FNBP1	SGCD	KDM5B
ERRFI1	LAMA5	PPP2R5D	EPN2	STK39	EEF1G	ETS2	DNAJC7	UBXN1	GPC4
VPS53	DOCK7	AKT3	SOS2	PIP5KL1	BHLHE40	PSME3	MAP1B	COX4I1	VPS37C
MCM7	NPC1	PSG1	ID2	LPIN3	MED13L	FLOT2	RBFOX1	PTPN21	SPP1
PMF1	SPDL1	MARK3	KIF5C	SKIL	CPS1	ARPC1B	EIF4E2	TFAP4	SEMA4C
PTPRU	PGRMC1	PPP1R12B	FGD4	STX6	GRIK3	ITGB4	PDE4A	SNAI1	DUSP10
KY	EFNA4	PRMT3	PTBP2	TOPBP1	FAM9B	PPIE	BMPER	UBE2D1	TADA2B
PSMC6	DOCK5	ITGA2B	COPG1	EXO1	KCNMB1	ATP6V1B2	DCP1A	CASQ2	RAB3B
LAMP2	JAM3	FARP2	EFNA3	MAML3	DDX39B	CHMP4C	GRK5	GPC3	WWC1
ARHGAP21	MCU	MAN2A2	NDUFS3	AGR2	XRCC1	TAF5L	PRKAB2	SMAD7	ARSG
LRP6	MYH8	OPRM1	DNAJC6	DDX23	MYOT	FKBP8	TNS4	SYT5	DENND1A
NTNG1	SNRPD1	ALOX5	ASAP1	CDKN2C	NBEA	CDKL5	ZDHHC17	BAZ1B	PTPN14
KCNV1	PDE3A	EIF3H	ADORA1	RPS6KB2	RPS15	HNRNPH2	MAP3K7	TUFM	DHX30
CHAF1A	NOC2L	KHDRBS3	OGN	SHROOM2	TRPC5	CNOT2	ARF6	KCNJ10	CDCA8
PCNT	GRN	PCCA	ANAPC1	IMMT	RPL14	PRDM16	KRT40	GRAP2	RORC
NPHP1	PDZRN3	SFN	PDGFD	SRA1	PDE3B	POLR2B	KCNJ12	ARHGEF12	USH2A
AKT2	PSMA3	TTR	CEP19	RPL28	FEN1	NGFR	PCF11	GALNT1	UBE2D3
THBS3	TUBGCP2	ZEB2	SLC9A3R2	PRIM1	EFNA1	SPTLC2	STXBP3	NFASC	NRIP1
MYOC	PHLPP2	MAP7D3	SNAP29	APC2	NECAP1	ASIC1	COX6C	C1QTNF2	TRIM33
MYO3A	GLI1	KCNA3	PIN4	SREBF1	PPP2R2D	TRAF5	DDX20	PI4KA	TEC
RPS3	MMP3	CEP44	SLX4	RTF1	SCAMP1	GORASP2	RB1CC1	PRPF31	COPS5
MGA	GLI2	COL14A1	ADAMTS4	KLC2	CSNK1D	USP7	MAPK8IP2	MITF	PAN2
SMO	CREM	DTNA	KIF13A	PGM5	ZNF263	PPP3CC	CDC23	THOC5	ATIC
ATP6V0A4	RAP1GAP	SUMF1	FLT4	LAPTM4B	RANBP3	STOM	RAB11FIP5	PCDHA10	COG2
HSPH1	TMEM192	AIFM1	CRTC2	ZFPL1	RXFP1	LOXL2	PRLR	GPAA1	MYH6
ECSIT	S1PR5	CFHR1	JKAMP	KCNQ4	NDUFA3	EYS	PSMD3	KATNAL1	PDP1
ERLIN1	ADAM12	EIF4A1	SNCAIP	BAG3	WDR6	SNIP1	SWAP70	CPE	DET1
MRPS15	RGS17	GYPB	SLC25A24	TACC1	SNX1	SLC16A2	GNG10	SMARCC2	MTCH1
EIF4G3	HCFC1	TAP2	ATP2B1	SCN2A	SYCE1	NR1D2	TEKT1	NUP210	TMEM205
KLHL2	GAD1	CDH15	FBXO7	SMCHD1	TGFBR3	RPL11	RNF8	SUSD4	ADD3
ITK	EOGT	LGALS3BP	CCNE1	ABCB10	SEMA3A	RELB	STC2	SDCBP	MEP1B
NCAPG2	PROS1	SPTLC1	VAMP8	HSD11B1	JAM2	DRD1	ACADVL	RARG	DGKZ
COL7A1	MAS1	BMP7	COLGALT2	PCBP1	ADAMTS2	DPP6	LMBR1L	PCGF1	PPFIA1
AP1M1	TXNDC12	RPS6KA1	CDCP1	TPGS2	TFDP2	UCHL5	GPR25	ARHGAP10	TMEM33
SUDS3	UMPS	PXDN	ADRA2A	MYO15A	CC2D2A	PYGL	DVL1	WLS	CYP1B1
CLDN19	IMMP1L	COPB2	GABRG1	NAP1L4	HAUS1	GBP2	SMOC2	ACAD9	GSPT2
SYNM	USP2	AIM2	POLDIP2	AGTR1	RABEP1	LIMD1	HNRNPR	SEC22A	RPL9
TPR	EYA2	NBR1	PDZD2	ESRRB	KNSTRN	KDM4A	FOXA2	RPRD2	EMC2
USP10	NCF2	MAP4K4	MATK	CDYL	IL17RC	PTBP1	RAB1A	RASD1	SDHC
SNX21	MAP2K2	KHDRBS1	AGAP1	COG5	MTA3	YBX3	LIPH	ABCE1	DOCK4
C1QTNF9	NDUFA8	SPEN	CLCA1	AHCYL1	NRG2	RHO	EMILIN2	HR	COPG2
TRIM11	CRX	ELMO3	UBXN2B	DHX36	SCN1B	MARK1	HCRTR1	S1PR4	SRP14

HSPA9	DNMT3B	CALML5	COL20A1	RRBP1	RCC2	NETO2	C8A	RAB35	NDUFS5
PRKAB1	SEC24C	CAP1	UBQLN2	SCN4B	DNAJC10	TNS3	POLE3	IARS2	MKNK1
SRSF10	LIMS1	CCNB1	BCAP31	GNAZ	SLC35A3	OXCT1	DNM1L	PKNOX2	EBF1
GLB1L2	COLEC12	STK38	LIN7C	GABRA2	GJB1	KCNMB2	KCNS3	PES1	PTGES3
ITGA3	CAMLG	MTCH2	PAX3	LAMTOR2	ZFYVE27	AGK	PCDHGB1	KCNH2	TACC2
FBXO25	TMEM237	VPS52	PKN2	KIF22	UBTF	KLHL1	CDH3	SOCS6	LEMD3
NIPBL	GPC6	CDK7	ATXN7L3	PIK3C3	OGG1	NPAT	OSMR	PPP5C	GAS7
CALCOCO2	SMARCD2	DYNLT3	UROD	BECN1	GART	HNF4G	ITGA2	STARD7	SORD
INTS7	SPATA13	TREX1	MRRF	RBM3	CD55	TSC22D1	DPYSL3	SGCB	GTF3C5
TRIM54	UHRF2	DCUN1D1	ABCC4	GAS8	RAB3IP	KTN1	DEGS1	NCS1	MEAF6
SYT17	GYG1	ACO2	NDUFAF2	ST7	LPCAT1	WDR77	WDFY3	ATPAF1	SKA3
FBXL7	POLK	PRMT2	CPT1A	PCDHA3	TUBGCP3	TOM1L1	AARS2	ARC	RBM45
GMPPA	SCLT1	TXK	QRFPR	MGST3	SLC39A8	PEX19	TLE4	PCK2	TCF7
COLEC11	CCAR2	USP4	ZNF638	NUP155	RIT2	SPATA18	PPP1R15A	SPTBN4	MYO1B
PRDX6	FOXC2	SIN3B	FOSB	SPECC1L	TONSL	NRP2	CC2D1A	NBEAL2	RGS3
U2SURP	MYL12A	GABARAPL1	IGF2BP3	RASSF1	LTV1	NAV2	TULP1	CACNA1H	P4HA1
IL17RD	KLHL20	VWF	RGS14	SRSF3	KPNA1	ABLIM2	JMJD1C	GDPD5	NEK9
OPA3	APOA1	HSF1	TAF13	C8B	CDA	ACACA	NAALADL2	CYCS	GLUL
FAT1	CERS2	THEM4	UBR3	WNK1	IL16	NAGPA	TMTC4	MAB21L2	NEK2
MAPKAPK3	PPA2	ANKRD1	RPL23	HMOX1	VGLL3	LYST	ARHGAP24	MAPK8IP3	MED12L
KLF11	PCDH20	DAB1	JAGN1	GALNT11	RGS16	ARHGEF11	PLCB4	IFT20	RTN4IP1
SLC17A2	ACSL3	IPO7	SERBP1	STT3B	SNRPB	NXF1	UBAC1	DSE	PKP3
EIF2S3	TH	MUC4	AIP	POGZ	RALY	MECP2	BABAM1	RPN2	TLK2
SLC4A7	AQR	EDA	HEY2	MTMR6	MYL9	TMTC3	MILR1	PBRM1	CTDP1
ZDHHC9	GOLGA7	ACTL6B	HSD17B12	PINK1	EHD4	TNKS1BP1	GABRA6	LPXN	CROCC
CASR	UNC93B1	LBR	RNF19A	EEF1B2	IMPDH2	SPAG16	CDH6	HCRTR2	PCDHGB4
CYP2S1	GET4	THSD7A	ITIH2	COL4A1	TNNT2	AFF3	ELP2	FGB	PTPRH
SPRED1	CTSD	ADORA2A	STK24	ALPP	CCP110	PRMT6	TK1	TCF20	KCNJ2
NUP205	DLX5	G6PD	GTF3C3	UBXN2A	ATP1B3	ATP2B4	MEN1	PAX6	KIRREL3
SOX9	TP53BP2	SLC25A13	STIM2	PLS3	HOXD13	SRSF11	GPRASP1	IPO11	MMS22L
PTGS2	DTL	PLCB2	HDGF	TRAK1	DKK3	SLC2A12	EPB42	ENC1	ARL4A
FAM83D	HSPA1B	GABRG3	TUBB4A	NFIB	SLC25A19	TANK	HNF1B	SMG7	TEAD4
DCAF7	FOXP4	BCKDHA	RBL2	THOC2	SLC7A5	CCSAP	SEMA5A	BAG1	MAP7
NR5A2	GJB7	KALRN	TSG101	LINGO2	S1PR2	GTF3C4	PITX2	TSHB	MICU1
BAG5	CSTF3	COCH	TRIM32	ERAP1	SS18L1	APLP2	EXTL3	ROCK2	RBFOX2
NUP153	P4HB	GNAT3	PPP1R9B	WIPF1	RNF123	ITGA6	FBXO45	SORL1	CHD1L
COPA	MXI1	ARPC5	SLC9A3	NEK7	AIMP1	PCBD1	VTI1A	NAP1L1	RALA
RAB14	STXBP2	KCNC2	CAPRIN1	TTC3	ORC1	SDC4	TLN2	CSTF2	CEACAM21
COMMD1	LIG3	MYH7B	BDNF	ADCY3	DRD3	XPO6	MOV10	SOCS3	PIK3C2B
LMX1B	MYRIP	KCNA1	MED6	CCDC88A	RGS4	GNB3	NDRG1	LAP3	ANKRD2
EPB41L2	CSDE1	RPAP2	CENPF	ATP2A3	RPL31	EID3	STAT4	KIDINS220	CRBN
NQO2	CLEC11A	LDB3	PELI1	FGG	NOP2	CPSF3	KMT2E	UCHL1	FARP1
PRMT5	CLIP1	EDEM3	RGS1	LRIG1	KIF3C	DYRK2	COX5B	GCDH	SETD7

COL2A1	TAF15	ANKS1A	JAKMIP2	CD28	SLAIN2	GORASP1	PCSK2	FOXM1	RPL7A
TAF7	RGS5	MSH5	BRD2	NCSTN	CLCN3	GCC2	HAUS5	PLOD2	TRIM23
MZT2B	PEBP1	IL31RA	MYOF	KRT9	DDX42	TPCN2	OTC	EIF4EBP1	MED11
PPP1R9A	WIPI2	ADRA1B	CKMT1A	POC1A	EPN3	ZFPM2	TOMM40	MZT1	BAG6
PTPN3	PHF1	OLFM3	PIK3C2A	B3GAT3	LGALS8	RAB8A	RPS4X	NTNG2	EHBP1
TTBK1	PNKP	CCHCR1	FSTL1	PA2G4	PKN3	FKBP4	BRPF3	C1D	ANTXR2
CDH10	ARF5	CCNC	MYH14	BCHE	HGF	MED20	PPP2R5A	MYO1E	IL17A
COX5A	CREB3	RGS20	GIT1	C3orf52	PARK7	POLR1C	AP4M1	DNALI1	RBM10
TRAK2	LAMA3	F11R	PRPF19	ANKRA2	SYT6	SYNJ2BP	NR5A1	GPR161	GSS
DYNLRB1	WASF2	RAC2	FOXD3	IQGAP2	DHCR24	TUBG2	REV1	ADCY1	CDC6
SEC62	DYNLT1	LCA5	HMG20A	CTPS2	KCTD3	MICU2	SH2B2	PLEKHF2	ATP4A
TRADD	S1PR1	CHD7	SUFU	IL20RA	FOXK2	APPBP2	JPH1	CUL4B	EIF3L
TDRD7	CD79B	CLTA	SPARC	INCA1	DNAJC5	RANBP9	UBR2	ALDH3A2	MED10
DIAPH1	SV2A	USP19	TRIP6	NAT10	RGS10	RABGEF1	TCOF1	RAB3C	ZFP36
VPS18	TIMM50	ABCA3	NTN4	GRK6	USO1	RPL37A	WDR26	RPL7	CEP95
PCDHGC3	STX7	METTL14	HOXC8	ELOVL5	SLC6A3	CD244	STAM2	TNNI2	MAPK11
RGMB	LMNB2	CUL4A	FAM110A	SKA1	CRTAC1	SSTR2	DEK	SORBS3	NRBF2
LRRCC1	RECQL4	MTF2	THRAP3	ITGA8	ANKRD46	CAMSAP3	MED21	TRIP11	LEPR
CIAO1	DNAJB6	SRSF7	ATF6B	NUP54	EXOC5				

Supplementary Table 4. Candidate AML genes present in all AML patients

MATN2	SMC3	UBC	PRKACA	FOXO1	BSN	FER	DCC	CEP120	EIF4E
KRT8	CASK	PRKD1	TNKS2	DYRK1A	EPB41L3	PRKCE	PPP2R2B	KAT2B	SMAD2
NRG1	RARB	VTN	RPS6KA2	CTNND2	LARP7	NFATC2	TNK2	CUL3	ANK2
HNRNPM	PDPK1	AKAP12	ELN	HNRNPU	KAT5	NTRK2	FAF1	SNW1	TANC1
SHANK1	TRPC3	CGN	MAP3K5	PRKG1	E2F3	KHDRBS2	HCK	STAT1	AMOT
BICD1	GSN	CTBP1	EXOC1	SF1	PXN	ITPR1	SMAD3	SMARCE1	SHC1
DYNC1I1	MCM3	POR	SPTAN1	ANK1	IFT74	SMARCC1	GRIN2B	ALMS1	POU2F1
HIPK2	CAMK2D	TP63	SNX9	KCNIP4	PDE4D	MAPK10	AP2M1	YWHAG	RGS7
CDC16	ACTN4	EZR	SREBF2	ESRRA	IQGAP1	ACTA1	FLNC	CDC5L	KCND2
MYO6	AP2S1	DLGAP2	CD2AP	ANXA7	THRA	SUMO1	ITGB5	NCOR2	MEF2A
HDAC6	RTN4	KCNB2	HNRNPD	NUDC	RBPJ	XRCC6	TSHR	PLA2G4A	PPM1A
GAN	TNKS	NTM	ATXN1	FBN1	TEAD2	MEF2C	DNM3	DAB2	DAG1
SIRT6	CHCHD3	DLGAP3	GRB2	HOMER1	PRKDC	SMAD1	RPA1	PTPRA	DTNBP1
HDAC7	CDC25C	PRKAR1A	ECT2	PTK2	DLG1	AMPH	PABPC1	AP2B1	CEP128
LATS2	KIF13B	PTPRB	MET	FYN	FOXJ2	S100A10	GRB10	CPSF6	HNRNPA1
NIN	CAMK2B	MYH9	TUBA1B	LYN	YWHAH	CARM1	PTPRK	EXOC2	PLEKHA7
MMP10	FAM20C	PTBP3	NCOA2	CALM3	CCT7	ADRB2	GRID2	PSEN1	GNA12
HDAC5	SMARCA4	LEF1	ETS1	PHGDH	TGFBR2	IMMP2L	KIAA0753	ACTA2	XRCC5
TYK2	SP1	TAB2	LMNA	VAV3	FBN2	SGK1	GAK	NME7	FLNA
EPHA4	NCOR1	PPP3CA	PPP1CB	EGLN3	SUMO2	NCOA3	RPA2	MAPKAP1	GJA1
GRM1	MACF1	KCNB1	GRIK2	TUBA1C	GNA13	CACNA1D	HMGB1	DLG2	ESR1

FBXW11	PRKAR2A	DISC1	TLN1	NEDD4	CEP170	CHUK	PDGFRA	NR2C2	RNF41
VDAC1	BTRC	ANK3	PLCG2	HDAC1	GAB1	CNTRL	SQSTM1	OPTN	ACTN1
CEP290	VCP	CDC20	EPHA2	BCAR1	YBX1	GRM7	CDK9	PHLDB2	CSNK1A1
ROBO1	TPM3	TNIK	NLGN1	AURKA	GNAO1	PRKCZ	BARD1	YWHAB	AKAP5
BLM	MLH1	MED13	PICK1	SH3GL3	DOCK1	MAPT	UBXN7	GRIK4	PPP2R1A
GRIA2	IRS1	PTPRR	APPL1	PRKCA	PTPRJ	ODF2	PRKACB	MED23	POU6F2
ARNT	COIL	DLC1	NINL	MAPK9	PCM1	EXOC6	CDC14A	SRPK2	NRXN1
ARRB1	ESR2	FUS	HMGA1	MYO19	TRAF6	RAD50	PPARD	YWHAZ	MTOR
MAD1L1	CEP135	HNRNPF	CUL1	ITSN1	EXOC4	LSAMP	KIF20A	NEB	RPS6KA3
MAGI1	MSN	BBS7	MED4	TUBB	NKX2-1	TERF1	ENO1	MAX	HTT
PPP3R1	WWTR1	CTNND1	ATR	PRKCI	RNF2	RXRG	STAU1	DHX15	PAK2
GNAI2	YES1	ACTN2	PLCG1	PIK3R3	AFAP1	ANLN	ROBO2	GRM3	PIBF1
CSNK2A1	TBP	MAML1	GNAI3	LRP2	EIF3E	TRAF3IP1	MAP3K3	CNTN1	ITGB1
VAV1	RELA	GRIK5	PTPRZ1	NR2F1	EXOC7	ABI2	RYR2	PPP2R5C	HDAC3
CAMK2G	TUBB3	TJP1	RPA3	PAFAH1B1	GRIA1	IKBKB	PRKAR2B	RBBP4	DLG3
PTPRG	LIMA1	PPARA	ACTB	SPTBN1	DNM1	RYR1	ABI1	TNS1	PPM1G
HSP90B1	SVIL	RHOA	CAMK2A	HSP90AB1	ERC1	KCNA2	SIRT1	SPICE1	PAK1
HOOK1	CCT4	TRIM28	THRB	RANGAP1	CENPJ	AHR	IGFBP5	TRIM24	GNAQ
CDH5	CSNK2A2	MAGI2	PLK1	DDX5	TSC2				

Supplementary Table 5. Candidate AML genes present in known AML gene modules for AML patient

<u>Sample 018</u>

**Candidate AML genes**	**Known AML genes** (of which the module includes candidate AML genes)	**ALL the AML genes involved in current module**	**Sample-ID**
ESR1	ACTBL2	ACTBL2,ESR1,NFKB1	18
ESR1	APEX1	APEX1,EGFR,ESR1,NPM1	18
ESR1	ARID1B	ARID1B,ESR1	18
ESR1	ASXL1	ASXL1,ESR1,KMT2C	18
ESR1	BCL3	BCL3,ESR1,TBL1XR1	18
ESR1	BCOR	BCOR,CREBBP,ESR1,FOS	18
ESR1	BLNK	BLNK,EGFR,ERBB4,ESR1,KIT,PDGFRB	18
ESR1	C2	C2,ESR1	18
ESR1	CAD	CAD,CDK4,CREBBP,ESR1,RARA	18
ESR1	CCND1	CCND1,EGFR,ESR1,PPARG	18
ESR1	CD6	CD6,ESR1	18
ESR1	CDH1	CDH1,CTNNB1,EGFR,ESR1,JUP	18
ESR1	CDK4	CDK4,CREBBP,ESR1,FOS,RARA	18
ESR1	CHD2	CHD2,ESR1,SMC1A	18
ESR1	CNTN2	CNTN2,ESR1,NCAM1,PTEN	18
ESR1	CREBBP	CREBBP,ESR1,RPL5	18

ESR1	CTNNB1	CDH1,CTNNB1,ERBB4,ESR1,JUP,PTEN	18
ESR1	DIO3	DIO3,ESR1,GNAI1	18
ESR1	E2F1	E2F1,ESR1	18
ESR1	ECT2L	ESR1,NFKB1	18
ESR1	EED	EED,ESR1	18
ESR1	EGFR	CDH1,CTNNB1,EGFR,ESR1,JUP	18
ESR1	EPM2A	CTNNB1,EPM2A,ESR1,FN1	18
ESR1	ERBB4	CTNNB1,ERBB4,ESR1,PTEN	18
ESR1	ETV6	ESR1,ETV6	18
ESR1	FN1	ESR1,FN1	18
ESR1	FOS	ESR1,FOS	18
ESR1	FUBP1	ESR1,FN1,FUBP1	18
ESR1	GAPDH	EGFR,ESR1,GAPDH,RPL5,SET	18
ESR1	GC	ERBB4,ESR1,GC	18
ESR1	GNAI1	ESR1,FOS,GNAI1	18
ESR1	HBA2	EGFR,ESR1,HBA2,HBB	18
ESR1	HBB	ESR1,HBA2,HBB	18
ESR1	HIF1A	ESR1,GAPDH,HIF1A,RPL5	18
ESR1	HLA-G	ESR1,HLA-G	18
ESR1	HNF4A	ESR1,HNF4A,MAPK14,PPARGC1A,SFPQ	18
ESR1	HPS4	ESR1,HPS4	18
ESR1	HSPA4	ESR1,HSPA4	18
ESR1	HSPA4L	ESR1,HSPA4L	18
ESR1	HSPA5	ESR1,HSPA5,RPL5	18
ESR1	JUNB	ESR1,FOS,JUNB,MAPK14	18
ESR1	JUP	CDH1,CTNNB1,EGFR,ESR1,JUP	18
ESR1	KDM6A	ESR1,KDM6A,KMT2C,MAPK14,NCOA6	18
ESR1	KIT	EGFR,ERBB4,ESR1,KIT,PDGFRB	18
ESR1	KMT2C	ESR1,KDM6A,KMT2C,MAPK14,NCOA6	18
ESR1	MAGED2	ESR1,MAGED2,PTEN	18
ESR1	MAP4K1	EGFR,ERBB4,ESR1,MAP4K1,SLC3A1	18
ESR1	MAPK14	ESR1,HSPA5,MAPK14	18
ESR1	MAPKAPK2	ESR1,MAPK14,MAPKAPK2	18
ESR1	MNDA	ESR1,MNDA,SET	18
ESR1	MPL	EGFR,ESR1,MPL,PDGFRB	18
ESR1	MSH6	ESR1,MSH6	18
ESR1	MUC1	ERBB4,ESR1,MUC1	18
ESR1	MVP	ESR1,MVP,PTEN	18
ESR1	MYO1G	ESR1,GNAI1,MYO1G	18
ESR1	NCAM1	CNTN2,EGFR,ESR1,NCAM1,SDC1	18
ESR1	NCOA6	ESR1,KMT2C,NCOA6,PPARG	18
ESR1	NFKB1	ESR1,HNF4A,NFKB1,PPARG	18
ESR1	NME2	ERBB4,ESR1,NME2,TBL1XR1	18

ESR1	NOS3	ESR1,NOS3	18
ESR1	NPM1	EGFR,ESR1,NPM1,TRRAP	18
ESR1	PDGFRB	EGFR,ERBB4,ESR1,KIT,PDGFRB	18
ESR1	PGR	ESR1,GNAI1,PGR,PTEN	18
ESR1	PHB2	ESR1,GAPDH,PHB2,SET	18
ESR1	PIK3CA	ESR1,PDGFRB,PIK3CA	18
ESR1	PKIA	ESR1,PKIA	18
ESR1	PPARG	EGFR,ESR1,NFKB1,PPARG	18
ESR1	PPARGC1A	ESR1,PPARGC1A,RARA,TBL1XR1	18
ESR1	PPP2R3A	EGFR,ESR1,PPP2R3A	18
ESR1	PSIP1	ESR1,FOS,PSIP1	18
ESR1	PSMC3	E2F1,ESR1,PSMC3	18
ESR1	PTEN	CTNNB1,ERBB4,ESR1,GNAI1,PTEN	18
ESR1	PTPRT	EGFR,ERBB4,ESR1,PTPRT,SLC3A1	18
ESR1	RAD21	ESR1,RAD21	18
ESR1	RAG2	ESR1,RAG2	18
ESR1	RARA	ESR1,PPARGC1A,RARA,TBL1XR1	18
ESR1	RB1	ESR1,PSMC3,RB1	18
ESR1	ROS1	CTNNB1,ESR1,ROS1	18
ESR1	RPL19	ESR1,RPL19	18
ESR1	RPL5	ESR1,RPL5	18
ESR1	SDC1	ERBB4,ESR1,SDC1	18
ESR1	SET	CTNNB1,ESR1,RPL5,SET	18
ESR1	SFPQ	CREBBP,E2F1,ESR1,SFPQ	18
ESR1	SKP1	ESR1,GAPDH,SET,SKP1	18
ESR1	SLC3A1	EGFR,ERBB4,ESR1,SLC3A1	18
ESR1	SMC1A	EGFR,ESR1,SMC1A	18
ESR1	SNRPE	ESR1,FN1,HSPA4,SNRPE	18
ESR1	STAG2	ESR1,RAD21,SMC1A,STAG2	18
ESR1	STAT5A	ERBB4,ESR1,STAT5A	18
ESR1	TBL1XR1	ESR1,PPARGC1A,RARA,TBL1XR1	18
ESR1	TOP2B	ESR1,TOP2B	18
ESR1	TRRAP	ESR1,TRRAP	18
ESR1	VDR	ESR1,PPARG,VDR	18
ESR1	XBP1	ESR1,HSPA5,MAPK14,XBP1	18
ESR1	ZMYM3	ESR1,ZMYM3	18
FYN	ABCG2	ABCG2,FYN	18
FYN	ABL1	ABL1,DNM2,EP300,FYN	18
FYN	ANPEP	ANPEP,CD36,FYN	18
FYN	BCR	BCR,DOK1,FYN,KDR	18
FYN	C5	C5,F2,FYN	18
FYN	CARD11	CARD11,FYN,ZAP70	18
FYN	CCND2	CCND2,FYN	18

FYN	CD14	CD14,FYN,LCK	18
FYN	CD19	CD19,FYN	18
FYN	CD2	CD2,CD58,FCGR3A,FYN,PTPRC	18
FYN	CD36	ANPEP,CD36,FYN	18
FYN	CD5	CD2,CD5,CD58,CD6,FYN,LCK,PTPRC,ZAP70	18
FYN	CD58	CD2,CD5,CD58,FYN,LCK,PTPRC	18
FYN	CD6	CD5,CD6,FYN	18
FYN	CD86	CD86,CTLA4,FYN,LCK	18
FYN	CSF1R	CSF1R,FYN	18
FYN	CSF2RB	BCR,CSF2RB,FYN,KIT	18
FYN	CTLA4	CD86,CTLA4,FYN	18
FYN	DLX4	ABL1,DLX4,FYN	18
FYN	DNM2	DNM2,FYN	18
FYN	DOK1	DOK1,FYN,KDR	18
FYN	DOK2	DOK2,FYN	18
FYN	EP300	DNM2,EP300,FYN	18
FYN	EPM2A	EPM2A,FYN	18
FYN	F2	C5,F2,FYN	18
FYN	FAS	FAS,FYN,LCK	18
FYN	FCGR3A	CD2,FCGR3A,FYN,PTPRC	18
FYN	FGFR2	FGFR2,FYN	18
FYN	FLT1	FLT1,FYN,KDR	18
FYN	FLT3	FLT3,FYN	18
FYN	GC	FYN,GC	18
FYN	GUCY2D	FYN,GUCY2D	18
FYN	HSP90AA1	FYN,HSP90AA1	18
FYN	HSPA8	FYN,HSPA8	18
FYN	ICOS	FYN,ICOS	18
FYN	IFNAR1	CD2,FCGR3A,FYN,IFNAR1,PTPRC,ZAP70	18
FYN	IL18R1	CD2,FYN,IL18R1,KIT,LCK	18
FYN	IL2RB	FYN,IL2RB,LCK	18
FYN	IL7	FYN,IL7,IL7R	18
FYN	IL7R	FYN,IL7,IL7R	18
FYN	ITGB2	FYN,ITGB2	18
FYN	KDR	BCR,FYN,KDR	18
FYN	KIT	FYN,KIT	18
FYN	LCK	FYN,LCK	18
FYN	MAP4K1	FYN,MAP4K1	18
FYN	MAT2A	FYN,MAT2A	18
FYN	MKI67	DNM2,EP300,FYN,MKI67	18
FYN	MPL	FYN,MPL,PTPRC	18
FYN	MS4A1	FYN,HSPA8,MS4A1,ZAP70	18
FYN	MYD88	FYN,MYD88	18

FYN	NCAM1	FYN,NCAM1	18
FYN	PIM1	ABL1,FYN,HSP90AA1,PIM1	18
FYN	PRDX2	FYN,PRDX2	18
FYN	PTPRC	FYN,LCK,PTPRC	18
FYN	RAPGEF1	FYN,RAPGEF1	18
FYN	SPN	FYN,SPN	18
FYN	THY1	CSF1R,FYN,LCK,THY1	18
FYN	ZAP70	FYN,ZAP70	18
FYN	ZMYM3	FYN,ZMYM3	18
LYN	ANGPT1	ANGPT1,ANGPT2,LYN,PTPRC	18
LYN	ANGPT2	ANGPT1,ANGPT2,LYN	18
LYN	BTK	BTK,JAK1,LYN	18
LYN	CASP9	CASP9,LYN	18
LYN	CD19	CD19,CD72,FCER2,LYN	18
LYN	CD22	CD22,INPP5D,LYN,PTPRC	18
LYN	CD36	CD36,CD9,LYN	18
LYN	CD68	CD68,LYN,MUC1	18
LYN	CD72	CD72,LYN	18
LYN	CD86	CD86,CTLA4,LCK,LYN,PIK3CA	18
LYN	CD9	CD36,CD9,LYN	18
LYN	CSF2RB	CSF2RB,INPP5D,JAK1,KIT,LYN	18
LYN	CSF3	CSF3,JAK1,LYN	18
LYN	CTLA4	CD86,CTLA4,GP1BA,LYN,POU2F2	18
LYN	DOK1	DOK1,LYN,PIK3CA	18
LYN	DOK2	DOK2,INPP5D,JAK1,LYN	18
LYN	EWSR1	EWSR1,LYN	18
LYN	FAT4	FAT4,LYN	18
LYN	FCER2	CD19,FCER2,ITGB2,LYN	18
LYN	FCGR3A	CD22,FCGR3A,LYN,PTPRC	18
LYN	FGFR2	FGFR2,LYN,PTK2B	18
LYN	FGR	FGR,LYN	18
LYN	FLT3	FLT3,LYN	18
LYN	GP1BA	GP1BA,LYN	18
LYN	IFNAR1	CD22,FCGR3A,IFNAR1,JAK1,LYN,PTPRC	18
LYN	IL1B	IL1B,LYN,TNFRSF10B	18
LYN	IL2RB	IL2RB,INPP5D,JAK1,LCK,LYN	18
LYN	IL5RA	CSF2RB,IL5RA,JAK1,LYN	18
LYN	IL6ST	IL6ST,JAK1,LIFR,LYN	18
LYN	IL7	IL7,IL7R,JAK1,LYN,PTK2B	18
LYN	IL7R	IL7,IL7R,JAK1,LYN	18
LYN	INPP5D	DOK2,INPP5D,JAK1,LYN	18
LYN	ITGAX	FCER2,ITGAX,ITGB2,LYN,PTK2B	18
LYN	ITGB2	ITGB2,LYN,PTK2B	18

LYN	JAK1	INPP5D,JAK1,LCK,LYN,PIK3CA	18
LYN	KIT	KIT,LYN	18
LYN	LCK	LCK,LYN	18
LYN	LIF	IL6ST,LIF,LIFR,LYN	18
LYN	LIFR	IL6ST,LIFR,LYN	18
LYN	MME	CD36,LYN,MME	18
LYN	MPL	LYN,MPL,PTK2B,PTPRC	18
LYN	MS4A1	LYN,MS4A1	18
LYN	MUC1	LYN,MUC1	18
LYN	MYD88	JAK1,LYN,MYD88,PTK2B	18
LYN	PBX1	LYN,PBX1	18
LYN	PIK3CA	INPP5D,JAK1,LCK,LYN,PIK3CA	18
LYN	POU2F2	LYN,PBX1,POU2F2	18
LYN	PRKCQ	LYN,PRKCQ	18
LYN	PTK2B	JAK1,LYN,PTK2B	18
LYN	PTPRC	JAK1,LCK,LYN,PTPRC	18
LYN	RPL10	LYN,RPL10	18
LYN	TNFRSF10B	IL1B,LYN,MYD88,TNFRSF10B	18
FAF1	ABCB1	ABCB1,FAF1,FAS	18
FAF1	AKT1	AKT1,FAF1,HSP90AA1	18
FAF1	ANGPT2	ANGPT2,CDKN1A,FAF1	18
FAF1	ATP2A2	ATP2A2,CA2,FAF1	18
FAF1	CA2	ATP2A2,CA2,FAF1,HSPA5,HSPD1	18
FAF1	CASP8	CASP8,FAF1,FAS,PARP1,RPL5	18
FAF1	CD40	CD40,FAF1,HSPA4,HSPA8,IL4R	18
FAF1	CD74	CD74,FAF1	18
FAF1	CDKN1A	CDKN1A,FAF1	18
FAF1	CEACAM6	CEACAM6,FAF1,HSPD1	18
FAF1	CREBBP	CREBBP,FAF1,RPL5	18
FAF1	CSF1R	CSF1R,FAF1	18
FAF1	DAZL	DAZL,FAF1	18
FAF1	DNTT	DNTT,FAF1	18
FAF1	FAS	CASP8,FAF1,FAS,HSPA5	18
FAF1	GAPDH	FAF1,FAS,GAPDH,RPL5,SET	18
FAF1	GHR	CREBBP,FAF1,GHR	18
FAF1	HIF1A	FAF1,GAPDH,HIF1A,RPL5	18
FAF1	HMBS	FAF1,HMBS	18
FAF1	HSP90AA1	FAF1,HSP90AA1	18
FAF1	HSPA4	FAF1,HSPA4	18
FAF1	HSPA4L	FAF1,HSPA4L,HSPA8	18
FAF1	HSPA5	CASP8,FAF1,HSPA5,RPL5	18
FAF1	HSPA8	FAF1,HSPA8	18
FAF1	HSPD1	FAF1,HSPD1	18

FAF1	IL4R	FAF1,IL4R	18
FAF1	IRF8	FAF1,IRF8	18
FAF1	LMOD1	FAF1,LMOD1,NPM1	18
FAF1	MAGED2	FAF1,MAGED2	18
FAF1	MRC2	FAF1,HSPD1,MRC2	18
FAF1	NEUROD1	FAF1,NEUROD1	18
FAF1	NPM1	FAF1,NPM1	18
FAF1	NPPA	FAF1,NPPA	18
FAF1	NR3C2	CREBBP,FAF1,HSP90AA1,HSPA4,NR3C2	18
FAF1	NR4A1	FAF1,NR4A1	18
FAF1	PARP1	FAF1,PARP1	18
FAF1	PHB2	FAF1,FAS,GAPDH,HSPA8,PHB2,SET	18
FAF1	POU2F2	FAF1,GAPDH,POU2F2	18
FAF1	RAG2	FAF1,RAG2	18
FAF1	RAPGEF1	FAF1,HSPA4,RAPGEF1	18
FAF1	RPL5	AKT1,CASP8,FAF1,FAS,RPL5	18
FAF1	RUNX1	CDKN1A,FAF1,HIF1A,RUNX1	18
FAF1	SET	FAF1,FAS,RPL5,SET	18
FAF1	SKP1	CASP8,FAF1,GAPDH,SET,SKP1	18
FAF1	SLC22A2	FAF1,PARP1,SLC22A2	18
FAF1	ST13	FAF1,ST13	18
FAF1	TET1	FAF1,HIF1A,TET1	18
FAF1	TNFSF10	FAF1,SET,TNFSF10,TNFSF13	18
FAF1	TNFSF13	FAF1,TNFSF10,TNFSF13	18
PRKCE	AHCY	AHCY,PRKCE	18
PRKCE	ANGPT2	ANGPT2,PRKCE	18
PRKCE	APP	APP,PRKCE	18
PRKCE	ATP2A2	ATP2A2,EGFR,PRKCE	18
PRKCE	BAD	BAD,BAX,HSPB1,MAPK3,PRKCE	18
PRKCE	BAX	BAX,HSP90AA1,HSPB1,MAPK3,PRKCE	18
PRKCE	BCOR	BCOR,MAPK3,PRKCE	18
PRKCE	BRAF	BAX,BRAF,HSPB1,MAPK3,PRKCE	18
PRKCE	BTK	BAX,BTK,HSPB1,PRKCE	18
PRKCE	CAD	CAD,PRKCE	18
PRKCE	CD2	CD2,CD22,CD58,PRKCE	18
PRKCE	CD22	CD22,EGFR,PRKCE	18
PRKCE	CD58	CD2,CD58,PRKCE	18
PRKCE	CD70	CD70,PRKCE	18
PRKCE	CEACAM1	CEACAM1,EGFR,PRKCE,SRC	18
PRKCE	CNR1	CNR1,EGFR,PRKCE	18
PRKCE	CTLA4	CTLA4,GP1BA,PRKCE	18
PRKCE	DLX4	DLX4,PRKCE,SRC	18
PRKCE	EGFR	EGFR,PRKCE	18

PRKCE	F2R	F2R,HSP90AA1,PRKCE	18
PRKCE	GP1BA	GP1BA,PRKCE	18
PRKCE	HBEGF	EGFR,HBEGF,PRKCE	18
PRKCE	HMBS	APP,HMBS,PRKCE	18
PRKCE	HSP90AA1	BAX,EGFR,HSP90AA1,HSPB1,MAPK3,PRKCE,SRC	18
PRKCE	HSPA8	EGFR,HSPA8,HSPB1,MAPK3,PRKCE,SRC	18
PRKCE	HSPB1	BAX,HSPB1,PRKCE,SRC	18
PRKCE	IER3	APP,EGFR,IER3,PRKCE	18
PRKCE	IFNAR2	IFNAR2,PRKCE	18
PRKCE	IL4R	IL4R,PRKCE	18
PRKCE	IL6	IL6,PRKCE,SRC	18
PRKCE	IL7R	IL7R,PRKCE	18
PRKCE	LMOD1	LMOD1,PRKCE	18
PRKCE	MAP4K1	BRAF,EGFR,MAP4K1,PRKCE,RAF1,SLC3A1	18
PRKCE	MAPK1	MAPK1,PRKCE	18
PRKCE	MAPK3	BAX,HSPB1,MAPK3,PRKCE	18
PRKCE	MRC2	MRC2,PRKCE,RAF1	18
PRKCE	MYO1G	MYO1G,PRKCE	18
PRKCE	PLEKHM1	EGFR,PLEKHM1,PRKCE	18
PRKCE	PTPRT	ATP2A2,EGFR,PRKCE,PTPRT,SLC3A1	18
PRKCE	RAF1	EGFR,HSP90AA1,PRKCE,RAF1,SRC	18
PRKCE	RASGRP3	BAX,PRKCE,RASGRP3	18
PRKCE	SAMSN1	PRKCE,RAF1,SAMSN1,SRC	18
PRKCE	SELL	PRKCE,SELL	18
PRKCE	SLC3A1	EGFR,PRKCE,SLC3A1	18
PRKCE	SNCA	MAPK1,MAPK3,PRKCE,SNCA	18
PRKCE	SRC	APP,MAPK1,PRKCE,SRC	18
PRKCE	TNFSF13	APP,PRKCE,TNFSF13	18
PRKCE	ZBTB16	PRKCE,ZBTB16	18
HDAC1	APEX1	APEX1,HDAC1	18
HDAC1	BCL11B	BCL11B,HDAC1	18
HDAC1	BCL2	BCL2,HDAC1	18
HDAC1	BCL3	BCL3,HDAC1	18
HDAC1	BIRC2	BIRC2,HDAC1	18
HDAC1	CASP1	CASP1,HDAC1	18
HDAC1	CDCA7L	CDCA7L,HDAC1,HOXA9,MEIS1	18
HDAC1	CDK1	CDK1,CEBPB,HDAC1,PML,TP73	18
HDAC1	CDKN1B	CDKN1B,DIABLO,HDAC1	18
HDAC1	CEBPB	CEBPB,HDAC1	18
HDAC1	CTCF	CTCF,HDAC1	18
HDAC1	DAXX	DAXX,DNMT1,DNMT3A,EZH2,HDAC1,SPI1	18
HDAC1	DIABLO	CDKN1B,DIABLO,HDAC1	18

HDAC1	DNMT1	DNMT1,DNMT3A,EZH2,HDAC1,SPI1	18
HDAC1	DNMT3A	DNMT3A,EZH2,HDAC1	18
HDAC1	ELF4	CTCF,ELF4,HDAC1,PML	18
HDAC1	ELL	ELL,HDAC1	18
HDAC1	EVPL	EVPL,HDAC1	18
HDAC1	EZH2	EZH2,GATA1,HDAC1	18
HDAC1	FHIT	FHIT,HDAC1	18
HDAC1	FOXP3	FOXP3,HDAC1	18
HDAC1	GATA1	EZH2,GATA1,HDAC1	18
HDAC1	GATA2	GATA2,HDAC1,MYB	18
HDAC1	HOXA9	CDCA7L,HDAC1,HOXA9	18
HDAC1	IL7R	EZH2,HDAC1,IL7R	18
HDAC1	KLF4	HDAC1,KLF4	18
HDAC1	MAP2K1	HDAC1,MAP2K1	18
HDAC1	MAPK8	HDAC1,MAPK8	18
HDAC1	MEIS1	CDCA7L,HDAC1,HOXA9,MEIS1	18
HDAC1	MLLT1	HDAC1,MLLT1,NSD1	18
HDAC1	MYB	HDAC1,MYB	18
HDAC1	NEUROD1	HDAC1,NEUROD1	18
HDAC1	NFE2	HDAC1,NFE2	18
HDAC1	NSD1	HDAC1,MLLT1,NSD1	18
HDAC1	PARP1	HDAC1,PARP1	18
HDAC1	PML	HDAC1,PML,TP73	18
HDAC1	RAPGEF1	HDAC1,RAPGEF1	18
HDAC1	RFC1	EZH2,HDAC1,RFC1	18
HDAC1	SAMSN1	HDAC1,SAMSN1	18
HDAC1	SF3B1	HDAC1,SF3B1	18
HDAC1	SFPQ	CEBPB,HDAC1,SFPQ,TP73	18
HDAC1	SMC1A	HDAC1,SMC1A	18
HDAC1	SPI1	DAXX,DNMT1,DNMT3A,EZH2,HDAC1,SPI1	18
HDAC1	SYK	HDAC1,SYK	18
HDAC1	TOP2B	HDAC1,PARP1,TOP2B	18
HDAC1	TP73	CEBPB,HDAC1,PML,TP73	18
HDAC1	USP18	HDAC1,USP18	18
HDAC1	ZBTB16	HDAC1,ZBTB16	18
DISC1	ABL1	ABL1,DISC1,DNM2,EP300	18
DISC1	ACTBL2	ACTBL2,DISC1	18
DISC1	ARID1A	ARID1A,DISC1	18
DISC1	ATP2A2	ATP2A2,DISC1	18
DISC1	CD22	CD22,DISC1	18
DISC1	CGA	CGA,DISC1	18
DISC1	CTLA4	CTLA4,DISC1	18
DISC1	CTNNA1	CTNNA1,DISC1	18

DISC1	DCK	DCK,DISC1	18
DISC1	DCTN1	DCTN1,DISC1	18
DISC1	DLX4	ABL1,DISC1,DLX4	18
DISC1	DNM2	DISC1,DNM2	18
DISC1	DNTT	DISC1,DNTT	18
DISC1	EFHC1	DISC1,EFHC1	18
DISC1	EGR1	DCTN1,DISC1,EGR1	18
DISC1	EP300	DISC1,DNM2,EP300	18
DISC1	EWSR1	DISC1,EWSR1	18
DISC1	EZH2	DISC1,EP300,EZH2	18
DISC1	FBXW7	DISC1,FBXW7	18
DISC1	FN1	DISC1,FN1	18
DISC1	IKZF1	DISC1,IKZF1	18
DISC1	IL4R	DISC1,IL4R	18
DISC1	IL6	DISC1,IL6	18
DISC1	IL7R	DISC1,EZH2,IL7R	18
DISC1	IL9R	DISC1,IL4R,IL7R,IL9R	18
DISC1	IRF8	DISC1,IRF8	18
DISC1	LMOD1	DISC1,LMOD1,NPM1	18
DISC1	LRP1	DISC1,LRP1,POT1	18
DISC1	MKI67	DISC1,DNM2,EP300,MKI67	18
DISC1	MLLT3	DISC1,MLLT3	18
DISC1	MVP	DISC1,MVP	18
DISC1	NPM1	DISC1,NPM1	18
DISC1	PFDN4	DISC1,PFDN4	18
DISC1	POT1	DISC1,POT1	18
DISC1	RAD21	DISC1,EZH2,FBXW7,RAD21	18
DISC1	RAG2	DISC1,RAG2	18
DISC1	SUZ12	DISC1,SUZ12	18
DISC1	TET1	DISC1,FBXW7,TET1	18
DISC1	THBS1	DISC1,THBS1	18
DISC1	UIMC1	DCTN1,DISC1,POT1,UIMC1	18
DISC1	ZMYM2	DISC1,ZMYM2	18
DISC1	ZNF79	DISC1,IKZF1,ZNF79	18
RPS6KA2	ANGPT2	ANGPT2,RPS6KA2	18
RPS6KA2	BAD	BAD,MAPK3,RPS6KA2	18
RPS6KA2	BCOR	BCOR,CREB1,CREBBP,FOS,MAPK3,RPL34,RPS6KA2	18
RPS6KA2	BRAF	BRAF,MAPK3,RPS6KA2	18
RPS6KA2	CD2	CD2,CD22,CD58,RPS6KA2	18
RPS6KA2	CD22	CD22,RPS6KA2	18
RPS6KA2	CD58	CD2,CD58,RPS6KA2	18
RPS6KA2	CD70	CD70,RPS6KA2	18

RPS6KA2	CEACAM1	CEACAM1,RPS6KA2	18
RPS6KA2	CLPB	CLPB,RPS6KA2	18
RPS6KA2	CR2	CR2,RPS6KA2	18
RPS6KA2	CREB1	CREB1,RPS6KA2	18
RPS6KA2	CREBBP	CREBBP,RPS6KA2	18
RPS6KA2	CTLA4	CTLA4,GP1BA,RPS6KA2	18
RPS6KA2	DDX10	DDX10,RPS6KA2	18
RPS6KA2	DLX4	DLX4,RPS6KA2	18
RPS6KA2	FGFR2	FGFR2,NISCH,RPS6KA2	18
RPS6KA2	FOS	FOS,RPS6KA2	18
RPS6KA2	GP1BA	GP1BA,RPS6KA2	18
RPS6KA2	HMBS	HMBS,RPS6KA2	18
RPS6KA2	HSPA8	HSPA8,MAPK3,RPS6KA2	18
RPS6KA2	IER3	IER3,RPS6KA2	18
RPS6KA2	IFNAR2	IFNAR2,RPS6KA2	18
RPS6KA2	IL4R	IL4R,RPS6KA2	18
RPS6KA2	IL6	CREB1,IL6,RPS6KA2	18
RPS6KA2	IL7R	IL7R,RPS6KA2	18
RPS6KA2	MAP2K1	CREBBP,MAP2K1,RPS6KA2	18
RPS6KA2	MAP4K1	BRAF,MAP4K1,RPS6KA2	18
RPS6KA2	MAPK3	MAPK3,RPS6KA2	18
RPS6KA2	NISCH	CREBBP,NISCH,RPL34,RPS6KA2	18
RPS6KA2	RPL34	FOS,MAP2K1,RPL34,RPS6KA2	18
RPS6KA2	SELL	RPS6KA2,SELL	18
RPS6KA2	TNFSF13	RPS6KA2,TNFSF13	18

Sample 027

**Candidate AML genes**	**Known AML genes** (of which the module includes candidate AML genes)	**ALL the AML genes involved in current module**	**Sample-ID**
ESR1	ASXL1	ASXL1,ESR1	27
ESR1	BCL3	BCL3,ESR1,MAPK3,NFKB1,TBL1XR1	27
ESR1	BLNK	BLNK,ESR1,PIK3R1	27
ESR1	CAD	CAD,ESR1	27
ESR1	CAV1	CAV1,ESR1,NFKB1	27
ESR1	CCND1	CCND1,ESR1,MSH6	27
ESR1	CDH1	CDH1,CTNNB1,ESR1,JUP	27
ESR1	CDK1	CDK1,ESR1,RXRA	27
ESR1	CEBPB	CEBPB,ESR1,HDAC9	27
ESR1	CHD2	CHD2,ESR1,SMC1A	27
ESR1	CLTC	CLTC,ESR1	27
ESR1	CNTN2	CNTN2,ESR1	27

ESR1	COX10	COX10,ESR1	27
ESR1	CREBBP	CREBBP,ESR1,MSH6,PBX1	27
ESR1	CTNNB1	CDH1,CTNNB1,ESR1,JUP	27
ESR1	DAXX	DAXX,ESR1,FOS	27
ESR1	DCT	DCT,ESR1	27
ESR1	DDX3X	DDX3X,ESR1	27
ESR1	DIO3	CTNNB1,DIO3,ESR1	27
ESR1	ERBB2	ERBB2,ESR1	27
ESR1	EWSR1	ESR1,EWSR1,SF3B1	27
ESR1	FOS	ESR1,FOS	27
ESR1	GAPDH	ERBB2,ESR1,GAPDH,SRC	27
ESR1	HDAC9	ESR1,HDAC9	27
ESR1	HLA-G	ESR1,HLA-G	27
ESR1	HNF4A	ESR1,HNF4A	27
ESR1	HSPA4L	ESR1,HSPA4L	27
ESR1	HSPA8	ESR1,HSPA8	27
ESR1	HSPD1	ESR1,HSPD1	27
ESR1	JAK2	ERBB2,ESR1,JAK2,MDM2	27
ESR1	JUNB	ESR1,JUNB	27
ESR1	JUP	CDH1,CTNNB1,ESR1,JUP	27
ESR1	LCK	CDH1,CTNNB1,ESR1,LCK	27
ESR1	LMOD1	CTNNB1,ESR1,LMOD1,NPM1	27
ESR1	MAPK3	ESR1,MAPK3	27
ESR1	MAPKAPK2	ESR1,MAPKAPK2	27
ESR1	MDM2	ESR1,MDM2,SRC	27
ESR1	MED12	ESR1,MED12	27
ESR1	MSH2	CREBBP,ESR1,MSH2,MSH6	27
ESR1	MSH6	ESR1,MSH2,MSH6	27
ESR1	MVP	ESR1,MVP,MYC	27
ESR1	MYC	CDH1,ESR1,HSPA8,MYC	27
ESR1	NCOA6	ESR1,NCOA6,PPARGC1A,RXRA	27
ESR1	NFKB1	ESR1,NFKB1	27
ESR1	NPM1	ERBB2,ESR1,JAK2,MDM2,NPM1,PIK3R1	27
ESR1	NPPA	ESR1,HNF4A,HSPD1,NPPA	27
ESR1	NRAS	ESR1,NRAS,SRC	27
ESR1	PACSIN3	ESR1,HSPA8,HSPD1,PACSIN3	27
ESR1	PARP1	ESR1,HDAC9,PARP1	27
ESR1	PBX1	CREBBP,ESR1,MDM2,PBX1	27
ESR1	PGR	ESR1,PARP1,PGR	27
ESR1	PIK3CA	ESR1,PIK3CA,PIK3R1	27
ESR1	PIK3R1	CCND1,ESR1,PIK3R1,PPARGC1A,RXRA,SRC	27
ESR1	PPARGC1A	ESR1,HNF4A,NFKB1,PPARGC1A,RXRA	27
ESR1	PPP2R3A	CDK1,ESR1,PPP2R3A	27

ESR1	ROS1	CTNNB1,ESR1,ROS1	27
ESR1	RTKN	CDH1,ESR1,JUP,RTKN	27
ESR1	RXRA	ESR1,FOS,PARP1,RXRA	27
ESR1	SCYL1	ESR1,SCYL1	27
ESR1	SDC1	ERBB2,ESR1,SDC1	27
ESR1	SET	ESR1,SET	27
ESR1	SF3B1	ESR1,SF3B1	27
ESR1	SFPQ	ESR1,SF3B1,SFPQ	27
ESR1	SKP1	ESR1,SKP1	27
ESR1	SKP2	CREBBP,ESR1,SKP2	27
ESR1	SMC1A	CTNNB1,ESR1,MSH6,SMC1A	27
ESR1	SNRPE	ESR1,SF3B1,SNRPE	27
ESR1	SRC	ESR1,RXRA,SRC	27
ESR1	SRSF2	ESR1,PPARGC1A,SRSF2	27
ESR1	STAT5A	ESR1,LCK,STAT5A	27
ESR1	TBL1XR1	CTNNB1,ESR1,TBL1XR1	27
ESR1	TCF15	ESR1,MSH2,MSH6,TCF15	27
ESR1	TET1	CREBBP,ESR1,STAT5A,TET1	27
ESR1	ZMYM3	ESR1,ZMYM3	27
ESR1	ZNF79	ESR1,ZNF79	27
FYN	BTK	BTK,CAV1,FYN	27
FYN	C3	C3,FYN	27
FYN	C5	C3,C5,FYN	27
FYN	CASP9	CASP9,FYN,RASA1,RPL5	27
FYN	CAV1	CAV1,FYN	27
FYN	CBL	CBL,FYN,LCK	27
FYN	CD19	BTK,CBL,CD19,CD79A,CR2,FYN,SYK	27
FYN	CD2	CD2,CD5,CD58,FYN,LCK	27
FYN	CD44	CD44,FYN	27
FYN	CD5	CBL,CD2,CD5,CD79A,FYN,LCK,RASA1,ZAP70	27
FYN	CD58	CD2,CD5,CD58,FYN	27
FYN	CD79A	CD79A,FYN,LCK	27
FYN	CD86	CD86,CTLA4,FYN,LCK	27
FYN	CR2	BTK,CBL,CD19,CD79A,CR2,FYN	27
FYN	CSF1R	CAV1,CBL,CSF1R,FYN,KIT,RASA1	27
FYN	CSF2RB	CSF2RB,EPOR,FYN,JAK2,KDR,KIT,SYK	27
FYN	CSPG4	CSPG4,FYN	27
FYN	CTLA4	CD86,CTLA4,FYN,JAK2,LCK,PTPN11	27
FYN	DLX4	DLX4,FYN	27
FYN	DNM2	DNM2,FYN	27
FYN	DOK1	CAV1,DOK1,DOK2,FYN	27
FYN	DOK2	DOK1,DOK2,FYN,LCK,RASA1	27

FYN	EPOR	CSF2RB,EPOR,FYN,JAK2,KIT,SYK	27
FYN	FAS	CAV1,FAS,FYN,LCK	27
FYN	FCGR3A	BTK,FCGR3A,FYN,LCK,SYK,ZAP70	27
FYN	FLT1	FLT1,FYN,KDR	27
FYN	GAPDH	FYN,GAPDH,SYK	27
FYN	GUCY2D	FYN,GUCY2D	27
FYN	ICOS	FYN,ICOS,SYK	27
FYN	IL18R1	CD2,FYN,IL18R1,LCK	27
FYN	IL1B	FYN,IL1B	27
FYN	IL2RB	FYN,IL2RB,LCK,SYK	27
FYN	IL7R	CBL,FYN,IL7R,KIT,MS4A1,SOCS1	27
FYN	JAK2	FYN,JAK2	27
FYN	KDR	CAV1,FLT1,FYN,KDR	27
FYN	KIT	CAV1,CD44,EPOR,FYN,IL7R,KIT	27
FYN	LCK	FYN,LCK	27
FYN	MAGED2	FYN,MAGED2	27
FYN	MAP4K1	FYN,MAP4K1	27
FYN	MAT2A	FYN,LCK,MAT2A	27
FYN	MS4A1	FYN,IL7R,LCK,MS4A1	27
FYN	NCAM1	FYN,NCAM1,SDC1	27
FYN	PRDX2	FYN,PRDX2	27
FYN	PTPN11	DNM2,FYN,LCK,PTPN11	27
FYN	RAPGEF1	FYN,RAPGEF1	27
FYN	RASA1	FYN,RASA1	27
FYN	RPL5	FYN,RASA1,RPL5	27
FYN	SDC1	BTK,FYN,NCAM1,SDC1	27
FYN	SNCA	FYN,SNCA	27
FYN	SOCS1	FYN,LCK,SOCS1	27
FYN	SPN	FYN,SPN	27
FYN	SYK	FYN,SYK	27
FYN	THY1	FYN,LCK,RASA1,THY1	27
FYN	TNFRSF10B	FYN,TNFRSF10B	27
FYN	ZAP70	FCGR3A,FYN,LCK,RASA1,ZAP70	27
HDAC1	AFF1	AFF1,HDAC1	27
HDAC1	APEX1	APEX1,HDAC1	27
HDAC1	BCL11B	BCL11B,HDAC1	27
HDAC1	BCL2	BCL2,HDAC1	27
HDAC1	BCL3	BCL3,EP300,HDAC1,NFKB2,TBL1XR1	27
HDAC1	BCOR	BCOR,CTCF,HDAC1	27
HDAC1	CAD	CAD,HDAC1	27
HDAC1	CBFA2T3	CBFA2T3,HDAC1	27
HDAC1	CBFB	CBFB,EP300,GATA3,HDAC1,RUNX1T1	27
HDAC1	CDCA7L	CDCA7L,HDAC1,HOXA9,PBX1	27

HDAC1	CDKN1A	CDKN1A,HDAC1	27
HDAC1	CEBPB	CEBPB,HDAC1,TP53	27
HDAC1	CHFR	CHFR,HDAC1	27
HDAC1	CTCF	CTCF,HDAC1	27
HDAC1	DLD	DLD,HDAC1	27
HDAC1	DNMT1	CEBPB,DNMT1,HDAC1,TP53	27
HDAC1	DNMT3A	DNMT1,DNMT3A,EZH2,HDAC1	27
HDAC1	EFHC1	CDCA7L,EFHC1,HDAC1,HOXA9,PBX1	27
HDAC1	EGR1	EGR1,HDAC1,KLF4	27
HDAC1	ELF4	ELF4,HDAC1	27
HDAC1	ELL	ELL,EP300,HDAC1,TP53	27
HDAC1	EP300	EP300,EZH2,GATA1,GATA3,HDAC1,STAT3	27
HDAC1	ETV6	ETV6,HDAC1,TP53	27
HDAC1	EZH2	EP300,EZH2,GATA1,GATA3,HDAC1,STAT3	27
HDAC1	FOXP3	EP300,FOXP3,HDAC1	27
HDAC1	GATA1	EP300,EZH2,GATA1,GATA3,HDAC1,STAT3	27
HDAC1	GATA2	GATA1,GATA2,HDAC1,TAL1	27
HDAC1	GATA3	EP300,EZH2,GATA1,GATA3,HDAC1,STAT3	27
HDAC1	HOXA9	HDAC1,HOXA9,PBX1	27
HDAC1	IDH2	HDAC1,IDH2	27
HDAC1	IKZF1	HDAC1,IKZF1	27
HDAC1	KLF4	EGR1,EP300,HDAC1,KLF4,TP53	27
HDAC1	NFKB2	HDAC1,NFKB2	27
HDAC1	NR3C1	HDAC1,NR3C1,TP53	27
HDAC1	PBX1	CDCA7L,HDAC1,HOXA9,PBX1	27
HDAC1	PFDN4	HDAC1,PFDN4	27
HDAC1	PHF6	HDAC1,PHF6	27
HDAC1	PIK3CA	HDAC1,PIK3CA	27
HDAC1	RUNX1T1	HDAC1,RUNX1T1	27
HDAC1	SALL4	EZH2,HDAC1,SALL4	27
HDAC1	SAMSN1	HDAC1,SAMSN1	27
HDAC1	SPI1	DNMT1,DNMT3A,HDAC1,SPI1	27
HDAC1	STAT3	EP300,EZH2,GATA1,GATA3,HDAC1,STAT3	27
HDAC1	STAT5A	EP300,HDAC1,STAT5A	27
HDAC1	TAL1	EP300,GATA3,HDAC1,TAL1	27
HDAC1	TBL1XR1	HDAC1,TBL1XR1	27
HDAC1	TOP2A	HDAC1,TOP2A,TOP2B	27
HDAC1	TOP2B	HDAC1,TOP2A,TOP2B	27
HDAC1	TP53	HDAC1,TP53	27
HDAC1	TP73	HDAC1,TP73	27
HDAC1	VEGFA	CEBPB,EP300,HDAC1,TP73,VEGFA	27
HDAC1	ZBTB16	HDAC1,TP53,ZBTB16	27
HDAC1	ZMYM2	HDAC1,ZMYM2	27

HDAC1	ZMYM3	HDAC1,ZMYM3	27
STAT1	ATM	ATM,STAT1	27
STAT1	BCL3	BCL3,STAT1	27
STAT1	CASP3	CASP3,DCTN1,STAT1	27
STAT1	CCR1	CCR1,STAT1	27
STAT1	CCR5	CCR5,CXCR4,JAK2,STAT1	27
STAT1	CD40	CD40,STAT1	27
STAT1	CSF2RB	CSF2RB,JAK2,KDR,KIT,STAT1	27
STAT1	CXCR4	CXCR4,STAT1	27
STAT1	DCTN1	DCTN1,STAT1	27
STAT1	DLD	DLD,STAT1	27
STAT1	FLT1	FLT1,KDR,STAT1	27
STAT1	FOXP3	FOXP3,STAT1	27
STAT1	GFAP	GFAP,STAT1	27
STAT1	HSPA4L	HSPA4L,STAT1	27
STAT1	IFNAR2	IFNAR2,ISG15,STAT1	27
STAT1	IFNG	IFNG,JAK2,STAT1	27
STAT1	IL1B	IL1B,IRAK1,IRF4,IRF8,STAT1	27
STAT1	IL2RA	IL2RA,IL2RB,IRF4,STAT1,TBX21	27
STAT1	IL2RB	IL2RA,IL2RB,STAT1	27
STAT1	IL6ST	IL6ST,LIF,STAT1	27
STAT1	IRAK1	IRAK1,STAT1	27
STAT1	IRF4	IL2RA,IL2RB,IRF4,STAT1,TBX21	27
STAT1	IRF8	IL1B,IRF8,ISG15,PTPN11,STAT1	27
STAT1	ISG15	ISG15,STAT1	27
STAT1	JAK2	JAK2,STAT1	27
STAT1	KDR	FLT1,KDR,STAT1	27
STAT1	KIT	KIT,STAT1	27
STAT1	LIF	IL6ST,LIF,STAT1	27
STAT1	MPL	JAK2,MPL,STAT1	27
STAT1	NOS2	NOS2,STAT1	27
STAT1	PIK3CA	PIK3CA,STAT1	27
STAT1	PML	PML,RXRA,STAT1	27
STAT1	PTPN11	IRF8,PTPN11,STAT1	27
STAT1	RXRA	RXRA,STAT1	27
STAT1	STAT5A	STAT1,STAT5A	27
STAT1	TBX21	IL2RA,IRF4,STAT1,TBX21	27
STAT1	TLR7	STAT1,TLR7	27
STAT1	TNFRSF1A	STAT1,TNFRSF1A	27
STAT1	TP73	STAT1,TP73	27
STAT1	VDR	RXRA,STAT1,VDR	27
LMNA	ATM	ATM,LMNA	27
LMNA	BIRC3	BIRC3,LMNA	27

LMNA	CAPN2	CAPN2,FLT3,LMNA	27
LMNA	CASP1	CASP1,EP300,LMNA	27
LMNA	CCND1	CCND1,LMNA	27
LMNA	CDK1	CDK1,LMNA	27
LMNA	CEBPE	CEBPE,LMNA	27
LMNA	CRKL	CRKL,LMNA	27
LMNA	CTLA4	CTLA4,LMNA	27
LMNA	DDX3X	DDX3X,LMNA	27
LMNA	EP300	EP300,EZH2,GATA1,GATA3,LMNA,STAT3	27
LMNA	EZH2	EP300,EZH2,GATA1,GATA3,LMNA,STAT3	27
LMNA	F2	F2,LMNA	27
LMNA	F2RL1	F2RL1,LMNA	27
LMNA	FLT3	CAPN2,FLT3,LMNA	27
LMNA	GATA1	EP300,EZH2,GATA1,GATA3,LMNA,STAT3	27
LMNA	GATA3	EP300,EZH2,GATA1,GATA3,LMNA,STAT3	27
LMNA	IL23A	IL23A,LMNA,STAT3	27
LMNA	IRAK1	IRAK1,LMNA	27
LMNA	ISG15	ISG15,LMNA	27
LMNA	MKI67	LMNA,MKI67	27
LMNA	MRGPRX3	LMNA,MRGPRX3	27
LMNA	MS4A1	LMNA,MS4A1	27
LMNA	PHB2	LMNA,PHB2	27
LMNA	PSIP1	LMNA,PSIP1	27
LMNA	RB1	LMNA,RB1	27
LMNA	SCYL1	LMNA,SCYL1	27
LMNA	SLC35B2	LMNA,SLC35B2	27
LMNA	SMC1A	LMNA,SMC1A	27
LMNA	STAT3	EP300,EZH2,GATA1,GATA3,LMNA,STAT3	27
LMNA	STX4	LMNA,STX4	27
LMNA	VAMP1	LMNA,STX4,VAMP1	27
LMNA	WTAP	LMNA,WTAP	27
GRB2	ABL1	ABL1,GRB2	27
GRB2	BLNK	BLNK,GRB2,PDGFRB	27
GRB2	CASP2	CASP2,GRB2	27
GRB2	CD19	CD19,CD22,CR2,GRB2	27
GRB2	CD22	CD19,CD22,GRB2,PTPRC	27
GRB2	CD72	BLNK,CD72,GRB2,PTPRC	27
GRB2	CD86	CD86,GRB2,PTPRC	27
GRB2	CDKN1B	CDKN1B,GRB2	27
GRB2	CR2	CD19,CD22,CR2,GRB2	27
GRB2	CSF1R	CSF1R,GRB2,PTPRC	27
GRB2	CSF3	CSF3,GRB2	27
GRB2	CSPG4	CSPG4,GRB2	27

GRB2	DLX4	ABL1,DLX4,GRB2	27
GRB2	DNM2	DNM2,GRB2	27
GRB2	DOK1	ABL1,DOK1,GRB2	27
GRB2	EGF	EGF,GRB2	27
GRB2	FLT1	FLT1,GRB2	27
GRB2	GC	GC,GRB2	27
GRB2	GHR	GHR,GRB2,PTPRC	27
GRB2	HSPA4	GRB2,HSPA4	27
GRB2	IL2RB	GRB2,IL2RB	27
GRB2	MAP4K1	CDKN1B,GRB2,MAP4K1	27
GRB2	MME	GRB2,MME	27
GRB2	MYD88	GRB2,MYD88	27
GRB2	PDGFRB	GRB2,PDGFRB,PTPRC	27
GRB2	PTPRC	GRB2,PTPRC	27
GRB2	RORA	GRB2,RORA	27
GRB2	SLC8A1	GRB2,SLC8A1	27
GRB2	SYNJ1	GRB2,SYNJ1	27
GRB2	TP73	DNM2,GRB2,TP73	27
GRB2	WNK2	GRB2,WNK2	27
PRKCA	BAD	BAD,PRKCA,SRC	27
PRKCA	BTK	BTK,PRKCA	27
PRKCA	CBL	CBL,LCK,PRKCA	27
PRKCA	CD4	CD4,FCGR3A,LCK,PRKCA,PRKCQ,SELL	27
PRKCA	CXCR4	CXCR4,GNAI1,PRKCA	27
PRKCA	DCTN1	DCTN1,PRKCA	27
PRKCA	DHRS9	DHRS9,PRKCA	27
PRKCA	F2RL1	F2RL1,PRKCA	27
PRKCA	FAS	FAS,LCK,PRKCA	27
PRKCA	FCGR3A	BTK,CD4,FCGR3A,LCK,PRKCA,SYK	27
PRKCA	FOS	FOS,GNAI1,PRKCA	27
PRKCA	GAPDH	GAPDH,PRKCA,RAF1,SRC,SYK	27
PRKCA	GNAI1	FOS,GNAI1,PRKCA	27
PRKCA	LCK	LCK,PRKCA	27
PRKCA	MUSK	MUSK,PRKCA	27
PRKCA	PARP4	PARP4,PRKCA,TERT	27
PRKCA	PIM1	BAD,CD4,PIM1,PRKCA	27
PRKCA	PRKCQ	PRKCA,PRKCQ	27
PRKCA	PTPN11	LCK,PRKCA,PTPN11	27
PRKCA	RAF1	PRKCA,RAF1	27
PRKCA	RASGRP3	PRKCA,PRKCQ,RASGRP3	27
PRKCA	SELL	CD4,PRKCA,PRKCQ,SELL	27
PRKCA	SI	PRKCA,SI	27
PRKCA	SLC9A3R1	PRKCA,SLC9A3R1	27

PRKCA	SRC	PRKCA,SRC	27
PRKCA	SRF	PRKCA,SRF	27
PRKCA	SYK	PRKCA,SYK	27
PRKCA	TERT	PRKCA,TERT	27
PRKCA	TOP2A	PRKCA,TOP2A	27
PRKCA	VAMP2	PRKCA,VAMP2	27

Sample 168

**Candidate AML genes**	**Known AML genes** (of which the module includes candidate AML genes)	**ALL the AML genes involved in current module**	**Sample-ID**
FYN	ABL1	ABL1,DNM2,EP300,FYN,MKI67	168
FYN	BAX	BAX,BRAF,FYN	168
FYN	BCL2	BAX,BCL2,FYN,MAPK3	168
FYN	BRAF	BRAF,FYN	168
FYN	BTK	BTK,CBL,FYN	168
FYN	C5	C5,F2,FYN	168
FYN	CARD11	BTK,CARD11,CBL,FYN	168
FYN	CBL	CBL,CD2,CD4,CD79A,CD8A,FCGR3A,FYN,IFNAR1,RASA1	168
FYN	CD14	CD14,FYN	168
FYN	CD2	CBL,CD2,CD58,CD79A,CD8A,FCGR3A,FYN,IFNAR1,JAK2,RASA1	168
FYN	CD4	CBL,CD2,CD4,CD79A,CD8A,FCGR3A,FYN,IFNAR1,RASA1	168
FYN	CD5	CD2,CD4,CD5,CD58,CD79A,FYN,RASA1,ZAP70	168
FYN	CD58	CD2,CD4,CD5,CD58,FYN	168
FYN	CD79A	CBL,CD4,CD79A,FYN,IFNAR1,JAK2,RASA1	168
FYN	CD86	CD4,CD86,CTLA4,FYN	168
FYN	CD8A	CD4,CD79A,CD8A,FYN,IFNAR1,JAK2,RASA1	168
FYN	CDH1	CDH1,FYN,MAPK3	168
FYN	CDK5	CDK5,FYN	168
FYN	CR2	CD79A,CR2,FYN	168
FYN	CSF1R	CBL,CSF1R,FYN,RASA1	168
FYN	CSF2RB	CSF2RB,FYN,JAK2	168
FYN	CTLA4	CD86,CTLA4,FYN,JAK2	168
FYN	DLX4	ABL1,DLX4,FYN	168
FYN	DNM2	DNM2,EP300,FYN,MKI67	168
FYN	DOK1	ABL1,DOK1,FYN	168
FYN	EP300	DNM2,EP300,FYN,MKI67	168
FYN	F2	C5,F2,FYN	168
FYN	FCGR3A	CBL,CD79A,FCGR3A,FYN,IFNAR1,JAK2,RAS	168

		A1	
FYN	FGFR2	FGFR2,FYN	168
FYN	FLT1	FLT1,FYN,KDR,VEGFA	168
FYN	FLT3	ABL1,CBL,FLT3,FYN,RASA1	168
FYN	GUCY2D	FYN,GUCY2D,HSPB1	168
FYN	HSPB1	FYN,HSPB1	168
FYN	IFNAR1	CBL,CD2,CD4,CD8A,FCGR3A,FYN,IFNAR1,JAK2,RASA1	168
FYN	IL15	FYN,IL15,IL2RB,IL2RG,JAK2,RAF1	168
FYN	IL1B	FYN,IL1B	168
FYN	IL2RB	FYN,IL15,IL2RB,IL2RG,JAK2,RAF1	168
FYN	IL2RG	CDH1,FYN,IL2RG	168
FYN	IL5RA	CSF2RB,FYN,IL5RA,JAK2	168
FYN	IL7R	CBL,FYN,IL2RG,IL7R	168
FYN	ITGA4	ABL1,FYN,ITGA4,TFRC	168
FYN	JAK2	CBL,CD2,CD4,CD79A,CD8A,FCGR3A,FYN,IFNAR1,JAK2,RASA1	168
FYN	KDR	FLT1,FYN,KDR,VEGFA	168
FYN	LIFR	FYN,LIFR	168
FYN	MAP4K1	BRAF,FYN,MAP4K1,MAPK3,RAF1	168
FYN	MAPK3	CD4,FYN,MAPK3,RASA1	168
FYN	MKI67	ABL1,EP300,FYN,MKI67	168
FYN	MPL	ABL1,CBL,FYN,JAK2,MPL,RAF1	168
FYN	PKIA	FYN,PKIA	168
FYN	RAF1	BRAF,FYN,RAF1	168
FYN	RAPGEF1	ABL1,CBL,FYN,RAPGEF1	168
FYN	RASA1	CBL,CD2,CD4,CD79A,CD8A,FCGR3A,FYN,IFNAR1,RASA1	168
FYN	SPN	FYN,SPN	168
FYN	TFRC	ABL1,FYN,TFRC	168
FYN	THY1	FYN,THY1	168
FYN	VEGFA	FYN,VEGFA	168
FYN	ZAP70	FYN,ZAP70	168
GRB2	ABL1	ABL1,DNM2,EP300,GRB2,MKI67	168
GRB2	ASXL1	ASXL1,GRB2	168
GRB2	BAD	BAD,GRB2	168
GRB2	BCR	ABL1,BCR,GRB2	168
GRB2	BLNK	BLNK,ERBB4,GRB2,KIT,PDGFRB	168
GRB2	CA2	CA2,GRB2,KIT	168
GRB2	CASP2	CASP2,GRB2	168
GRB2	CCR5	CCR5,DNM2,GRB2	168
GRB2	CD19	CD19,CD22,GRB2,INPP5D	168
GRB2	CD22	CD19,CD22,GRB2	168

GRB2	CD72	BLNK,CD72,GRB2	168
GRB2	CD86	CD86,GRB2	168
GRB2	CR2	CD19,CD22,CR2,GRB2	168
GRB2	CSF1R	CSF1R,GRB2,KIT	168
GRB2	CSF3	CSF3,GRB2	168
GRB2	DLX4	ABL1,DLX4,GRB2	168
GRB2	DNM2	DNM2,EP300,GRB2,MKI67	168
GRB2	DOK1	ABL1,DOK1,GRB2,PTPN11	168
GRB2	EGR1	EGR1,GRB2	168
GRB2	EP300	DNM2,EP300,GRB2,MKI67	168
GRB2	EPO	EPO,EPOR,GRB2,KIT	168
GRB2	EPOR	EPO,EPOR,GRB2,KIT	168
GRB2	ERBB4	ABL1,ERBB4,GRB2	168
GRB2	EWSR1	EWSR1,GRB2	168
GRB2	FGFR2	FGFR2,GRB2	168
GRB2	FLT1	FLT1,GRB2	168
GRB2	FLT3	ABL1,FLT3,GRB2	168
GRB2	GHR	GHR,GRB2	168
GRB2	IL15	EGR1,GRB2,IL15,IL2RB	168
GRB2	IL2RB	GRB2,IL15,IL2RB	168
GRB2	IL5RA	BCR,EPOR,GRB2,IL5RA,KIT	168
GRB2	IL9R	ABL1,GRB2,IL9R	168
GRB2	INPP5D	CD22,GRB2,INPP5D	168
GRB2	KIT	BLNK,GRB2,KIT,PDGFRB	168
GRB2	LIFR	GRB2,LIFR	168
GRB2	MAP4K1	GRB2,MAP4K1	168
GRB2	MKI67	ABL1,EP300,GRB2,MKI67	168
GRB2	MPL	ABL1,GRB2,MPL	168
GRB2	MYH11	GRB2,MYH11	168
GRB2	NME2	ERBB4,GRB2,NME2	168
GRB2	PDGFRB	GRB2,KIT,PDGFRB	168
GRB2	PTPN11	DNM2,GRB2,PDGFRB,PTPN11	168
GRB2	RAPGEF1	ABL1,GRB2,RAPGEF1	168
GRB2	SLC8A1	GRB2,SLC8A1	168
GRB2	SOCS1	GRB2,SOCS1	168
GRB2	SYNJ1	GRB2,SYNJ1	168
GRB2	TF	GRB2,TF	168
GRB2	WNK2	GRB2,WNK2	168
GRB2	ZAP70	ERBB4,GRB2,ZAP70	168
LYN	ANGPT1	ANGPT1,ANGPT2,LYN,PTPRC,TEK	168
LYN	ANGPT2	ANGPT1,ANGPT2,LYN,TEK	168
LYN	BCL2	BCL2,LYN	168
LYN	CARD11	CARD11,LYN,PRKCQ	168

LYN	CASP9	CASP9,LYN	168
LYN	CD2	CD2,CD22,CD79A,CD8A,FCGR3A,LYN,PTPRC,SRC	168
LYN	CD22	CD22,LYN,PIK3R1,PTPRC	168
LYN	CD36	CD36,CD9,LYN,SRC	168
LYN	CD72	CD72,LYN,PTPRC	168
LYN	CD79A	CD22,CD79A,LYN,PTPRC,SRC	168
LYN	CD8A	CD22,CD79A,CD8A,LYN,PTPRC,SRC	168
LYN	CD9	CD36,CD9,LYN,SRC	168
LYN	CSF2RB	CSF2RB,LYN,PIK3R1,PTPRC	168
LYN	CSF3	CSF3,LYN	168
LYN	CTLA4	CTLA4,LYN,PIK3R1	168
LYN	DOK1	DOK1,DOK2,LYN,PIK3R1,SRC	168
LYN	DOK2	DOK2,LYN,SRC	168
LYN	ERBB4	ERBB4,LYN,SRC	168
LYN	EWSR1	EWSR1,LYN,SRC	168
LYN	FCGR3A	CD22,CD79A,FCGR3A,LYN,PTPRC,SRC	168
LYN	FGR	FGR,LYN	168
LYN	FLT3	FLT3,LYN,PIK3R1,SRC	168
LYN	IL1B	IL1B,LYN	168
LYN	IL2RB	IL2RB,LYN,PIK3R1	168
LYN	IL5RA	CSF2RB,IL5RA,LYN,PIK3R1	168
LYN	IL6	IL6,IL6ST,LYN	168
LYN	IL6ST	IL6,IL6ST,LIF,LYN	168
LYN	IL7R	IL7R,LYN,PIK3R1	168
LYN	INPP5D	CD22,DOK2,INPP5D,LYN,PIK3R1	168
LYN	ITGB2	ITGB2,LYN	168
LYN	LIF	IL6,IL6ST,LIF,LYN	168
LYN	MME	CD9,LYN,MME	168
LYN	MPL	LYN,MPL,SRC	168
LYN	NME2	ERBB4,LYN,NME2	168
LYN	PIK3R1	LYN,PIK3R1,SRC	168
LYN	PRKCQ	LYN,PRKCQ	168
LYN	PTPRC	CD22,CD79A,LYN,PTPRC,SRC	168
LYN	SRC	LYN,PIK3R1,SRC	168
LYN	TEK	ANGPT1,ANGPT2,LYN,PIK3R1,TEK	168
LYN	TNFSF13B	LYN,TNFSF13B	168
RELA	AATF	AATF,RELA	168
RELA	AFF1	AFF1,MLLT1,NSD1,RELA,VDR	168
RELA	ATM	ATM,RELA,SOCS1	168
RELA	BCL2L11	BCL2L11,DIABLO,RARA,RELA	168
RELA	BTK	BCL2L11,BTK,RELA	168
RELA	CASP1	CASP1,RELA	168

RELA	CASP8	CASP8,PARP1,RELA,SET	168
RELA	CDX2	CDX2,RELA	168
RELA	CEBPB	CEBPB,RELA,STAT6	168
RELA	CXCR4	CXCR4,RELA	168
RELA	CYP24A1	CYP24A1,RELA	168
RELA	DIABLO	DIABLO,RELA	168
RELA	ELF4	ELF4,RELA,SET	168
RELA	IL1B	IL1B,IRF8,RELA	168
RELA	IL7R	IL7R,RELA,SOCS1	168
RELA	IRF8	IL1B,IRF8,RELA	168
RELA	MLLT1	AFF1,MLLT1,NSD1,RELA,VDR	168
RELA	NCOA6	NCOA6,NR3C1,PPARGC1A,RELA,RXRA,VDR	168
RELA	NEUROD1	NEUROD1,RELA	168
RELA	NFKBIB	NFKBIB,RELA	168
RELA	NR3C1	BCL2L11,DIABLO,NR3C1,RELA	168
RELA	NR4A1	NR4A1,RELA,RXRA	168
RELA	NSD1	AFF1,MLLT1,NSD1,RELA	168
RELA	PARP1	PARP1,PBX1,RELA,RXRA,VDR	168
RELA	PBX1	PARP1,PBX1,RELA,RXRA	168
RELA	PGR	PGR,RELA,STAT5A	168
RELA	PIK3CA	PIK3CA,RELA	168
RELA	PPARGC1A	NR3C1,PPARGC1A,RELA,RXRA	168
RELA	PSMC3	PSMC3,RELA	168
RELA	RARA	BCL2L11,DIABLO,RARA,RELA	168
RELA	RPL5	CASP8,PARP1,RELA,RPL5,SET	168
RELA	RXRA	PARP1,PBX1,RELA,RXRA	168
RELA	SET	CASP8,PARP1,RELA,SET	168
RELA	SOCS1	RELA,SOCS1	168
RELA	STAT5A	CEBPB,NR3C1,RELA,STAT5A	168
RELA	STAT6	CEBPB,RELA,STAT6	168
RELA	TERT	RELA,TERT	168
RELA	VDR	RELA,RXRA,VDR	168
HDAC1	AFF1	AFF1,HDAC1,MLLT1	168
HDAC1	BCL11B	BCL11B,HDAC1,JUN	168
HDAC1	BCL3	BCL3,HDAC1	168
HDAC1	BCOR	BCOR,HDAC1,JUN	168
HDAC1	CBFB	CBFB,HDAC1,MLLT1,RARA	168
HDAC1	CDK1	CDK1,CREBBP,HDAC1,JUN,PML	168
HDAC1	CDX2	CDX2,CREBBP,HDAC1	168
HDAC1	CREB1	CREB1,HDAC1	168
HDAC1	CREBBP	CREB1,CREBBP,HDAC1,JUN	168
HDAC1	DNTT	DNTT,HDAC1	168
HDAC1	ELF4	ELF4,HDAC1,PML	168

HDAC1	EZH2	EZH2,GATA1,GATA3,HDAC1	168
HDAC1	FHIT	FHIT,HDAC1	168
HDAC1	FOS	CREBBP,FOS,HDAC1,JUN,PML,TP73	168
HDAC1	GATA1	EZH2,GATA1,GATA3,HDAC1	168
HDAC1	GATA3	EZH2,GATA1,GATA3,HDAC1	168
HDAC1	HDAC9	HDAC1,HDAC9,JUN	168
HDAC1	JUN	HDAC1,HDAC9,JUN	168
HDAC1	MAPK8	HDAC1,HDAC9,JUN,MAPK8	168
HDAC1	MEIS1	CREBBP,HDAC1,MEIS1,PBX1	168
HDAC1	MLLT1	AFF1,HDAC1,MLLT1	168
HDAC1	NFE2	HDAC1,NFE2,RARA	168
HDAC1	NR3C1	CREBBP,HDAC1,JUN,NR3C1	168
HDAC1	PBX1	HDAC1,PBX1	168
HDAC1	PFDN4	HDAC1,PFDN4	168
HDAC1	PML	CREBBP,HDAC1,JUN,PML,TP73	168
HDAC1	RARA	CREBBP,HDAC1,JUN,RARA	168
HDAC1	SALL4	EZH2,HDAC1,SALL4	168
HDAC1	SAMSN1	HDAC1,SAMSN1	168
HDAC1	TOP2B	HDAC1,TOP2B	168
HDAC1	TP73	CREBBP,HDAC1,JUN,PML,TP73	168

Sample 270

**Candidate AML genes**	**Known AML genes** (of which the module includes candidate AML genes)	**ALL the AML genes involved in current module**	**Sample-ID**
FYN	ANPEP	ANPEP,FYN	270
FYN	BCOR	BCOR,FYN	270
FYN	BTK	BTK,CD19,FYN	270
FYN	C5	C5,F2,FYN	270
FYN	CARD11	CARD11,FYN	270
FYN	CASP8	CASP8,FYN	270
FYN	CASP9	CASP9,FYN,MAPK3,RASA1	270
FYN	CD19	CD19,CD22,FYN	270
FYN	CD2	CD2,CD5,CD58,FYN,LCK	270
FYN	CD22	BTK,CD19,CD22,CD79A,FYN	270
FYN	CD5	CD2,CD5,CD58,CD72,CD79A,FYN,LCK,RASA 1	270
FYN	CD58	CD2,CD5,CD58,FYN,LCK	270
FYN	CD72	CD2,CD5,CD72,CD79A,FYN,LCK,RASA1	270
FYN	CD79A	CD19,CD79A,FYN	270
FYN	CD86	CD86,CTLA4,FYN,LCK	270
FYN	CDH1	CDH1,FYN	270

FYN	CRKL	CRKL,DNM2,FYN	270
FYN	CSF1R	CSF1R,FYN,RASA1	270
FYN	CTLA4	CD86,CTLA4,FYN,LCK	270
FYN	DLX4	DLX4,FYN	270
FYN	DNM2	DNM2,FYN	270
FYN	DOK1	CRKL,DOK1,FYN	270
FYN	DOK2	CRKL,DOK2,FYN	270
FYN	EDN1	EDN1,FYN	270
FYN	F2	C5,F2,FYN	270
FYN	FLT3	CRKL,FLT3,FYN,KITLG,RASA1	270
FYN	GUCY2D	FYN,GUCY2D	270
FYN	IL15	FYN,IL15,IL2RA,IL2RB,LCK	270
FYN	IL2RA	FYN,IL15,IL2RA,IL2RB,LCK	270
FYN	IL2RB	FYN,IL15,IL2RA,IL2RB	270
FYN	IL5RA	FYN,IL5RA	270
FYN	IL7R	FYN,IL2RB,IL7R	270
FYN	KITLG	CRKL,FLT3,FYN,IL7R,KITLG	270
FYN	LCK	CDH1,DNM2,FYN,LCK	270
FYN	LIFR	FYN,LIFR	270
FYN	MAPK3	FYN,MAPK3,RASA1	270
FYN	MBP	FYN,MBP	270
FYN	MPL	FYN,MPL	270
FYN	MRC2	CASP8,FYN,MRC2	270
FYN	NCAM1	FYN,NCAM1	270
FYN	PTPRT	FYN,PTPRT	270
FYN	RASA1	FYN,MAPK3,RASA1	270
FYN	SNCA	FYN,MAPK3,SNCA	270
FYN	SPN	FYN,SPN	270
FYN	TGOLN2	FYN,TGOLN2	270
FYN	THY1	FYN,LCK,THY1	270
FYN	TNFRSF10B	FYN,TNFRSF10B	270
FYN	VEGFA	FYN,VEGFA	270
ESR1	ABCC1	ABCC1,ESR1	270
ESR1	ACTBL2	ACTBL2,ESR1	270
ESR1	APEX1	APEX1,ESR1	270
ESR1	BCL3	BCL3,ESR1,TBL1XR1	270
ESR1	BLNK	BLNK,ESR1	270
ESR1	BRAF	BRAF,ESR1	270
ESR1	CDH1	CDH1,ESR1	270
ESR1	CDK1	CDK1,ESR1	270
ESR1	CNTN2	CNTN2,ESR1,NCAM1	270
ESR1	DNTT	DNTT,ESR1	270
ESR1	EDNRA	EDNRA,ESR1	270

ESR1	EPM2A	EPM2A,ESR1	270
ESR1	FBXW7	ESR1,FBXW7	270
ESR1	FUBP1	ESR1,FUBP1	270
ESR1	HLA-G	ESR1,HLA-G	270
ESR1	HSPA4L	ESR1,HSPA4L	270
ESR1	JAK1	ESR1,JAK1	270
ESR1	JAK2	ESR1,JAK1,JAK2,RET	270
ESR1	JUP	CDH1,ESR1,JUP	270
ESR1	LCK	CDH1,ESR1,LCK	270
ESR1	MAPKAPK2	ESR1,MAPKAPK2	270
ESR1	MERTK	ESR1,MERTK	270
ESR1	MPL	ESR1,JAK2,MPL	270
ESR1	MSH2	ESR1,MSH2	270
ESR1	MSH6	ESR1,MSH6	270
ESR1	MVP	ESR1,MVP	270
ESR1	NCAM1	CNTN2,ESR1,NCAM1	270
ESR1	NOS3	ESR1,NOS3	270
ESR1	NPPA	ESR1,NPPA	270
ESR1	PACSIN3	ESR1,PACSIN3	270
ESR1	PHB2	ESR1,PHB2	270
ESR1	PPP2R3A	ESR1,PPP2R3A	270
ESR1	PTPRT	ESR1,JAK1,MSH2,PTPRT	270
ESR1	RAG2	CDH1,ESR1,RAG2,SKP2	270
ESR1	RET	ESR1,RET	270
ESR1	RORA	ESR1,RORA	270
ESR1	ROS1	CDH1,ESR1,JAK2,ROS1	270
ESR1	RPL19	ESR1,RPL19	270
ESR1	SETBP1	ESR1,PPP2R3A,SETBP1	270
ESR1	SKP2	ESR1,JAK2,SKP2	270
ESR1	STAT6	ESR1,JAK1,STAT6	270
ESR1	TBL1XR1	ESR1,TBL1XR1	270
ESR1	TET1	ESR1,MSH2,MSH6,RORA,TET1	270
ESR1	TOP2A	ESR1,TOP2A	270
ESR1	XIAP	ESR1,XIAP	270
GRB2	ASXL1	ASXL1,GRB2	270
GRB2	BCR	BCR,CRKL,GRB2	270
GRB2	BLNK	BLNK,CD72,GRB2	270
GRB2	BTK	BTK,CD19,GRB2	270
GRB2	CD19	CD19,CD22,CR2,GRB2,PTPRC	270
GRB2	CD22	BTK,CD19,CD22,CR2,GRB2,PTPRC	270
GRB2	CD72	BLNK,CD72,GRB2	270
GRB2	CD86	CD86,GRB2,PTPRC	270
GRB2	CDKN1B	CDKN1B,GRB2	270

GRB2	CLPB	CLPB,GRB2	270
GRB2	CR2	CD19,CR2,GRB2	270
GRB2	CRKL	CRKL,DNM2,GRB2	270
GRB2	CSF1R	CSF1R,GRB2	270
GRB2	CSF2RB	CSF2RB,EPO,EPOR,GRB2,KITLG,PTPRC	270
GRB2	CSF3	CSF3,GRB2	270
GRB2	DLX4	DLX4,GRB2	270
GRB2	DNM2	DNM2,GRB2	270
GRB2	EGF	EGF,GRB2	270
GRB2	EPO	CSF2RB,EPO,EPOR,GRB2,KITLG,PTPRC	270
GRB2	EPOR	CSF2RB,EPOR,GRB2,KITLG,PTPRC	270
GRB2	FLT3	CRKL,FLT3,GRB2,KITLG	270
GRB2	GC	GC,GRB2,TF	270
GRB2	GFAP	GFAP,GRB2	270
GRB2	GHR	GHR,GRB2,PTPRC	270
GRB2	IL15	GRB2,IL15,IL2RA,IL2RB,IL2RG	270
GRB2	IL2RA	GRB2,IL15,IL2RA,IL2RB,IL2RG	270
GRB2	IL2RB	GRB2,IL15,IL2RA,IL2RB,IL2RG	270
GRB2	IL2RG	GRB2,IL2RB,IL2RG,MERTK	270
GRB2	IL5RA	BCR,CSF2RB,EPOR,GRB2,IL5RA	270
GRB2	ITGA4	DNM2,GRB2,ITGA4	270
GRB2	KDR	GRB2,KDR	270
GRB2	KITLG	CRKL,EPOR,FLT3,GRB2,KITLG	270
GRB2	LIFR	GRB2,LIFR	270
GRB2	MERTK	GRB2,IL2RG,MERTK	270
GRB2	NCOA6	GRB2,NCOA6	270
GRB2	NISCH	GRB2,NISCH	270
GRB2	PTPRC	CD86,GRB2,PTPRC	270
GRB2	ROS1	GRB2,ROS1	270
GRB2	SI	GRB2,SI	270
GRB2	TF	GRB2,TF	270
GRB2	WNK2	GRB2,WNK2	270
PRKACA	ABL1	ABL1,HRAS,MAPK1,PRKACA	270
PRKACA	ATP2A2	ATP2A2,CA2,CFTR,PRKACA	270
PRKACA	B3GAT1	B3GAT1,HRAS,PRKACA	270
PRKACA	BAD	BAD,PRKACA	270
PRKACA	BIRC3	BIRC3,GAPDH,PRKACA	270
PRKACA	BRAF	BRAF,KRAS,MAPK1,PRKACA	270
PRKACA	CA2	ATP2A2,CA2,PRKACA	270
PRKACA	CAD	CAD,PRKACA	270
PRKACA	CAV1	ABL1,CAV1,CFTR,MAPK1,PRKACA	270
PRKACA	CDK2	CDK2,HRAS,MAPK1,PIK3R1,PRKACA	270
PRKACA	CFTR	CFTR,PRKACA	270

PRKACA	CLTC	CFTR,CLTC,PRKACA	270
PRKACA	CRABP1	CRABP1,MAPK1,PRKACA	270
PRKACA	GAPDH	GAPDH,MAPK1,PRKACA	270
PRKACA	GFAP	GFAP,PRKACA	270
PRKACA	HIF1A	HIF1A,PRKACA	270
PRKACA	HNF4A	HNF4A,PRKACA	270
PRKACA	HRAS	CDK2,HRAS,MAPK1,PIK3R1,PRKACA	270
PRKACA	HSPA4	CFTR,HSPA4,PRKACA	270
PRKACA	HSPA8	HSPA8,PRKACA	270
PRKACA	IFNAR1	IFNAR1,MAPK1,PRKACA	270
PRKACA	ITGA4	ITGA4,PRKACA	270
PRKACA	KRAS	HRAS,KRAS,MAPK1,PRKACA	270
PRKACA	LRP1	LRP1,PRKACA	270
PRKACA	MAPK1	CDK2,HRAS,MAPK1,PIK3R1,PRKACA	270
PRKACA	MAT1A	CDK2,MAT1A,PRKACA,XPO1	270
PRKACA	MDM2	CFTR,MDM2,PRKACA	270
PRKACA	NR3C1	MAPK1,NR3C1,PRKACA	270
PRKACA	PCNA	CDK2,HRAS,MAPK1,PCNA,PIK3R1,PRKACA	270
PRKACA	PIK3R1	CDK2,HRAS,MAPK1,PIK3R1,PRKACA	270
PRKACA	PKIA	CDK2,HSPA4,IFNAR1,ITGA4,NR3C1,PIK3R1,PKIA,PRKACA,RAF1,XPO1	270
PRKACA	PTK2B	PRKACA,PTK2B	270
PRKACA	RAF1	CFTR,PRKACA,RAF1	270
PRKACA	RASGRP3	HRAS,PRKACA,RASGRP3	270
PRKACA	TNFRSF10B	PRKACA,TNFRSF10B	270
PRKACA	TYMS	KRAS,PRKACA,TYMS	270
PRKACA	XPO1	PRKACA,XPO1	270
HDAC1	AFF1	AFF1,HDAC1	270
HDAC1	APEX1	APEX1,HDAC1	270
HDAC1	BCL11B	BCL11B,HDAC1	270
HDAC1	BCL3	BCL3,HDAC1	270
HDAC1	CDCA7L	CDCA7L,HDAC1,HOXA9	270
HDAC1	CDK1	CDK1,EZH2,HDAC1	270
HDAC1	CDKN1B	CDKN1B,DIABLO,HDAC1,MAPK8	270
HDAC1	CHFR	CHFR,HDAC1	270
HDAC1	DIABLO	CDKN1B,DIABLO,HDAC1	270
HDAC1	DNMT1	DNMT1,HDAC1	270
HDAC1	DNTT	DNTT,HDAC1	270
HDAC1	EZH2	EZH2,GATA1,HDAC1	270
HDAC1	FBXW7	FBXW7,HDAC1	270
HDAC1	FLI1	FLI1,HDAC1,PML	270
HDAC1	FOXP3	FOXP3,HDAC1,HDAC9	270
HDAC1	GATA1	EZH2,GATA1,GATA3,HDAC1	270

HDAC1	GATA3	EZH2,GATA1,GATA3,HDAC1,MAPK14	270
HDAC1	HDAC9	HDAC1,HDAC9	270
HDAC1	HIF1A	HDAC1,HIF1A	270
HDAC1	HOXA9	CDCA7L,HDAC1,HOXA9	270
HDAC1	IDH2	HDAC1,IDH2	270
HDAC1	LYL1	HDAC1,LYL1	270
HDAC1	MAPK14	HDAC1,HDAC9,MAPK14	270
HDAC1	MAPK8	HDAC1,HDAC9,MAPK8	270
HDAC1	MEIS1	CDCA7L,HDAC1,HOXA9,MEIS1	270
HDAC1	NEUROD1	GATA3,HDAC1,NEUROD1	270
HDAC1	NR3C1	HDAC1,NR3C1	270
HDAC1	PFDN4	HDAC1,PFDN4	270
HDAC1	PHF6	HDAC1,PHF6	270
HDAC1	PML	HDAC1,PML	270
HDAC1	RUNX1T1	HDAC1,RUNX1T1	270
HDAC1	SALL4	EZH2,HDAC1,SALL4	270
HDAC1	SAMSN1	HDAC1,IDH2,SAMSN1,SYK	270
HDAC1	SPI1	HDAC1,MAPK8,SPI1	270
HDAC1	SYK	HDAC1,HDAC9,SYK	270
HDAC1	ZBTB16	HDAC1,HDAC9,ZBTB16	270
HDAC1	ZMYM3	HDAC1,ZMYM3	270
SMARCA4	ARID1A	ARID1A,ARID1B,SMARCA4	270
SMARCA4	ARID1B	ARID1A,ARID1B,SMARCA4	270
SMARCA4	CDCA7L	CDCA7L,HOXA9,PBX1,SMARCA4	270
SMARCA4	CDX2	CDX2,SMARCA4	270
SMARCA4	CEBPB	CEBPB,SMARCA4	270
SMARCA4	CHFR	CHFR,PARP1,SMARCA4	270
SMARCA4	CREB1	CREB1,SMARCA4	270
SMARCA4	EED	EED,SMARCA4,SUZ12	270
SMARCA4	FANCA	FANCA,SMARCA4	270
SMARCA4	FBXW7	FBXW7,SMARCA4	270
SMARCA4	FLI1	CEBPB,FLI1,PML,SMARCA4	270
SMARCA4	GATA1	GATA1,SMARCA4	270
SMARCA4	HIF1A	HIF1A,RUNX1,SMARCA4	270
SMARCA4	HOXA9	CDCA7L,HOXA9,PBX1,SMARCA4	270
SMARCA4	JUNB	JUNB,SMARCA4	270
SMARCA4	KLF4	KLF4,SMARCA4	270
SMARCA4	MAPK8	MAPK8,SMARCA4	270
SMARCA4	MEIS1	CDCA7L,CREB1,HOXA9,MEIS1,PBX1,SMARCA4	270
SMARCA4	NEUROD1	NEUROD1,SMARCA4	270
SMARCA4	NF1	NF1,SMARCA4	270
SMARCA4	NR3C1	CREB1,NR3C1,SMARCA4	270

SMARCA4	PARP1	PARP1,SMARCA4	270
SMARCA4	PBX1	NR3C1,PBX1,SMARCA4	270
SMARCA4	PML	PML,SMARCA4	270
SMARCA4	PPARG	PPARG,SMARCA4	270
SMARCA4	RUNX1	RUNX1,SMARCA4,ZBTB16	270
SMARCA4	RXRA	RXRA,SMARCA4	270
SMARCA4	SFPQ	RUNX1,SFPQ,SMARCA4	270
SMARCA4	STAT5A	CEBPB,SMARCA4,STAT5A	270
SMARCA4	SUZ12	EED,SMARCA4,SUZ12	270
SMARCA4	SYK	SMARCA4,SYK	270
SMARCA4	TERT	SMARCA4,TERT	270
SMARCA4	TOP2A	SMARCA4,TOP2A	270
SMARCA4	TOP2B	PARP1,SMARCA4,TOP2A,TOP2B	270
SMARCA4	ZBTB16	RXRA,SMARCA4,ZBTB16	270
SHC1	AMD1	AMD1,SHC1	270
SHC1	ANGPT1	ANGPT1,CSF1R,SHC1	270
SHC1	BCR	BCR,SHC1	270
SHC1	CD19	CD19,SHC1	270
SHC1	CLPB	CLPB,IGF1R,SHC1	270
SHC1	CSF1R	CSF1R,SHC1	270
SHC1	CTLA4	CTLA4,JAK2,PTPN11,SHC1	270
SHC1	DOK1	DOK1,SHC1	270
SHC1	DOK2	DOK2,SHC1	270
SHC1	EGF	EGF,SHC1	270
SHC1	EPO	EPO,JAK2,SHC1	270
SHC1	FCGR3A	FCGR3A,SHC1,ZAP70	270
SHC1	FGFR2	FGFR2,SHC1	270
SHC1	FLT3	FLT3,SHC1	270
SHC1	GHR	GHR,IGF1R,SHC1	270
SHC1	IGF1R	IGF1R,SHC1	270
SHC1	IL15	IL15,IL2RA,IL2RB,IL2RG,JAK2,SHC1	270
SHC1	IL2RA	IL15,IL2RA,IL2RB,IL2RG,JAK2,SHC1	270
SHC1	IL2RB	IL15,IL2RA,IL2RB,IL2RG,SHC1	270
SHC1	IL2RG	IL2RB,IL2RG,MERTK,SHC1	270
SHC1	IL4R	IL2RG,IL4R,SHC1	270
SHC1	JAK2	IGF1R,JAK2,SHC1	270
SHC1	KDR	KDR,SHC1	270
SHC1	MAT1A	MAT1A,MAT2A,SHC1	270
SHC1	MAT2A	MAT1A,MAT2A,SHC1	270
SHC1	MERTK	IL2RG,MERTK,SHC1	270
SHC1	MPL	IGF1R,JAK2,MPL,SHC1	270
SHC1	PTPN11	PTPN11,SHC1	270
SHC1	RAPGEF1	RAPGEF1,SHC1	270

SHC1	RPL5	RPL5,SHC1	270
SHC1	TBX21	SHC1,TBX21	270
SHC1	ZAP70	SHC1,ZAP70	270

Sample 472

**Candidate AML genes**	**Known AML genes** (of which the module includes candidate AML genes)	**ALL the AML genes involved in current module**	**Sample-ID**
ESR1	ACTBL2	ACTBL2,ESR1	472
ESR1	ANKRD49	ANKRD49,ESR1,XPO1	472
ESR1	ASXL1	ASXL1,ESR1,KMT2C	472
ESR1	ATP2A2	ATP2A2,ESR1	472
ESR1	BCL2L1	BCL2L1,ESR1,GNAI1,PTEN	472
ESR1	BCL3	BCL3,ESR1,NFKB1	472
ESR1	BRCA1	BRCA1,ESR1,MSH2	472
ESR1	CAD	CAD,ESR1,XPO1	472
ESR1	CAV1	CAV1,ESR1	472
ESR1	CCND1	BRCA1,CCND1,ESR1,MSH6,XPO1	472
ESR1	CDKN1A	CDKN1A,ESR1,MSH2	472
ESR1	CEBPB	CEBPB,ESR1,HDAC9,NFKB1	472
ESR1	CHD2	CHD2,ESR1,SMC1A	472
ESR1	CNTN2	CNTN2,ESR1,NCAM1,PTEN	472
ESR1	COX10	COX10,ESR1	472
ESR1	CTNNB1	CTNNB1,ESR1,SMC1A	472
ESR1	DAXX	DAXX,ESR1,XPO1	472
ESR1	DCT	DCT,ESR1	472
ESR1	DLX4	DLX4,ESR1	472
ESR1	DNTT	DNTT,ESR1	472
ESR1	ECT2L	ECT2L,ESR1,NFKB1	472
ESR1	ERBB4	ERBB4,ESR1	472
ESR1	EWSR1	ESR1,EWSR1,FN1,GAPDH	472
ESR1	FN1	ESR1,FN1	472
ESR1	GAPDH	ESR1,FN1,GAPDH,HSPD1,XPO1	472
ESR1	GNAI1	BCL2L1,ESR1,GNAI1	472
ESR1	HDAC9	ESR1,HDAC9	472
ESR1	HLA-G	ESR1,HLA-G	472
ESR1	HNF4A	ESR1,HNF4A,XPO1	472
ESR1	HPS4	ESR1,HPS4,RAN	472
ESR1	HSPA4	ESR1,HSPA4,HSPD1	472
ESR1	HSPA8	ESR1,HSPA8	472
ESR1	HSPD1	ESR1,FN1,HSPD1	472
ESR1	ICOS	ERBB4,ESR1,ICOS	472

ESR1	IGF1R	ESR1,IGF1R,NFKB1,NRAS	472
ESR1	JAK2	BRCA1,ESR1,HSPA8,IGF1R,JAK2	472
ESR1	JUP	CTNNB1,ESR1,JUP,MSH2	472
ESR1	KDM6A	ESR1,KDM6A,KMT2C,MAPK14	472
ESR1	KMT2C	ESR1,KDM6A,KMT2C,MAPK14	472
ESR1	LCK	ESR1,LCK,PIK3CA	472
ESR1	LRP1B	ESR1,HSPA8,LRP1B	472
ESR1	MAPK14	ESR1,MAPK14	472
ESR1	MED12	ESR1,MED12,XPO1	472
ESR1	MRC2	ESR1,HSPD1,MRC2	472
ESR1	MSH2	BRCA1,ESR1,MSH2,MSH6	472
ESR1	MSH6	BRCA1,ESR1,MSH2,MSH6,SMC1A	472
ESR1	MUC1	CEBPB,ERBB4,ESR1,MUC1	472
ESR1	MVP	BRCA1,ESR1,MVP,PARP4	472
ESR1	NCAM1	CNTN2,ESR1,NCAM1,SDC1	472
ESR1	NF1	ESR1,NF1	472
ESR1	NFKB1	ESR1,MSH2,NFKB1	472
ESR1	NME2	ERBB4,ESR1,NME2	472
ESR1	NOS3	CAV1,ESR1,NOS3	472
ESR1	NPPA	ESR1,HNF4A,NPPA	472
ESR1	NRAS	ESR1,IGF1R,NFKB1,NRAS	472
ESR1	PACSIN3	ESR1,HSPA8,PACSIN3,RPL10	472
ESR1	PARP1	ESR1,FN1,HDAC9,PARP1	472
ESR1	PARP4	BRCA1,CAV1,ESR1,MVP,PARP4	472
ESR1	PGR	BRCA1,ESR1,GNAI1,PARP1,PGR	472
ESR1	PICALM	ESR1,FN1,PICALM	472
ESR1	PIK3CA	ESR1,PIK3CA	472
ESR1	PPP2R3A	ESR1,PPP2R3A	472
ESR1	PTEN	CTNNB1,ERBB4,ESR1,GNAI1,PTEN	472
ESR1	PTPRT	ATP2A2,ERBB4,ESR1,PTPRT	472
ESR1	RAG2	BRCA1,ESR1,RAG2	472
ESR1	RAN	ESR1,RAN	472
ESR1	RARA	ESR1,FN1,RARA	472
ESR1	RPL10	ESR1,RPL10,RPL5	472
ESR1	RPL19	ESR1,RPL10,RPL19,RPL5	472
ESR1	RPL5	ESR1,FN1,RPL5,XPO1	472
ESR1	RTKN	ESR1,RTKN	472
ESR1	RXRA	ESR1,RARA,RXRA	472
ESR1	SDC1	ESR1,SDC1	472
ESR1	SET	ESR1,FN1,GAPDH,SET,XPO1	472
ESR1	SKP1	ESR1,SKP1	472
ESR1	SMC1A	BRCA1,ESR1,MSH2,MSH6,SMC1A	472
ESR1	SNRPE	ESR1,FN1,SNRPE	472

ESR1	SPN	CTNNB1,ESR1,MUC1,SPN	472
ESR1	SUZ12	ESR1,SUZ12	472
ESR1	TCF15	ESR1,MSH2,SUZ12,TCF15	472
ESR1	TF	ESR1,FN1,TF	472
ESR1	TFRC	CAV1,ESR1,TF,TFRC	472
ESR1	TG	ESR1,TG	472
ESR1	TLR7	ESR1,TLR7,XPO1	472
ESR1	TOP2A	ESR1,TOP2A	472
ESR1	TRRAP	ESR1,TRRAP	472
ESR1	XBP1	ESR1,XBP1	472
ESR1	XPO1	ERBB4,ESR1,XPO1	472
ESR1	ZBTB16	ESR1,HDAC9,ZBTB16	472
FYN	ABL1	ABL1,FYN,JAK2	472
FYN	ANPEP	ANPEP,FYN	472
FYN	BCR	BCR,CRKL,FYN	472
FYN	BTK	BTK,FYN,JAK1	472
FYN	C5	C5,FYN	472
FYN	CASP3	CASP3,FYN,RASA1	472
FYN	CD14	CD14,FYN,LCK	472
FYN	CD19	BTK,CD19,CD22,CD79A,CR2,FYN	472
FYN	CD2	CD2,CD58,FYN,LCK	472
FYN	CD22	CD19,CD22,CD79A,CR2,FYN	472
FYN	CD33	CD33,FYN,RASA1,ZAP70	472
FYN	CD58	CD2,CD58,FYN,LCK	472
FYN	CD79A	CD19,CD79A,FYN	472
FYN	CD86	CD86,CTLA4,FYN,LCK	472
FYN	CDK5	CDK5,FYN	472
FYN	CR2	CD19,CD22,CD79A,CR2,FYN	472
FYN	CRKL	CRKL,FYN,ZAP70	472
FYN	CSF1R	CSF1R,FYN,KIT,RASA1	472
FYN	CSF2RB	BCR,CSF2RB,FYN,JAK1,JAK2,KIT	472
FYN	CTLA4	CD86,CTLA4,FYN,JAK2,LCK,PTPN11	472
FYN	CTNNB1	CTNNB1,FYN	472
FYN	DNM2	DNM2,FYN	472
FYN	DOK1	ABL1,CRKL,DOK1,FYN,PTPN11	472
FYN	F2	C5,F2,FYN	472
FYN	FAS	FAS,FYN	472
FYN	FASLG	FASLG,FYN	472
FYN	FCGR1A	CRKL,FCGR1A,FYN,ZAP70	472
FYN	FLT1	CRKL,CTNNB1,FLT1,FYN,KDR,VEGFA	472
FYN	FLT3	CRKL,FLT3,FYN,RASA1	472
FYN	GAPDH	FYN,GAPDH	472
FYN	GUCY2D	FYN,GUCY2D	472

FYN	ICOS	CD19,CRKL,FYN,ICOS	472
FYN	IL15	FYN,IL15,IL2RA,IL2RB,IL7R	472
FYN	IL2RA	FYN,IL2RA,IL2RB	472
FYN	IL2RB	FYN,IL15,IL2RA,IL2RB	472
FYN	IL7	FYN,IL7,IL7R,SOCS1	472
FYN	IL7R	FYN,IL2RB,IL7,IL7R,KIT,MS4A1,SOCS1	472
FYN	JAK1	FYN,JAK1	472
FYN	JAK2	FYN,JAK2	472
FYN	KDR	FLT1,FYN,KDR,VEGFA	472
FYN	KIT	FYN,IL7R,KIT	472
FYN	KITLG	FLT3,FYN,IL7R,KIT,KITLG	472
FYN	LCK	CD86,FYN,LCK	472
FYN	MAP4K1	CASP3,FYN,MAP4K1	472
FYN	MAT2A	FYN,MAT2A	472
FYN	MBP	FYN,MBP	472
FYN	MS4A1	FYN,IL7,IL7R,KIT,LCK,MS4A1	472
FYN	NCAM1	FYN,NCAM1	472
FYN	PKIA	FYN,PKIA	472
FYN	PTPN11	CRKL,FYN,PTPN11	472
FYN	RAPGEF1	CRKL,FYN,RAPGEF1	472
FYN	RASA1	FYN,RASA1,ZAP70	472
FYN	RHOH	CRKL,FYN,RASA1,RHOH,ZAP70	472
FYN	SNCA	FYN,SNCA	472
FYN	SOCS1	FYN,SOCS1	472
FYN	SPN	CTNNB1,FYN,SPN	472
FYN	THY1	FYN,LCK,THY1	472
FYN	VEGFA	CTNNB1,FYN,VEGFA	472
FYN	ZAP70	CRKL,FYN,LCK,ZAP70	472
GRB2	ASXL1	ASXL1,GRB2	472
GRB2	BCR	BCR,CRKL,GRB2	472
GRB2	BLNK	BLNK,BTK,CD72,CRKL,GRB2	472
GRB2	BTK	BTK,GRB2,JAK1	472
GRB2	CASP2	CASP2,GRB2	472
GRB2	CCR5	CCR5,CD4,DNM2,GRB2,JAK2,LCK	472
GRB2	CD19	BTK,CD19,CR2,GRB2	472
GRB2	CD1A	CD1A,GRB2	472
GRB2	CD4	CD4,CD86,GRB2,LCK,PTPRC	472
GRB2	CD72	BLNK,CD4,CD72,GRB2,LCK,ZAP70	472
GRB2	CD86	CD4,CD86,GRB2,LCK,PTPRC	472
GRB2	CDKN1B	CDKN1B,GRB2,JAK2	472
GRB2	CR2	CD19,CR2,GRB2	472
GRB2	CRKL	CRKL,GRB2,ZAP70	472
GRB2	CSF1R	CSF1R,GRB2,KIT	472

GRB2	CSF3	CSF3,GRB2,JAK1,JAK2	472
GRB2	DLX4	DLX4,GRB2	472
GRB2	DNM2	DNM2,GRB2	472
GRB2	DOK1	CRKL,DOK1,DOK2,GRB2,INPP5D,PTPN11	472
GRB2	DOK2	CRKL,DOK2,GRB2,INPP5D,JAK1	472
GRB2	E2F1	E2F1,GRB2	472
GRB2	EGF	EGF,FGFR2,GRB2	472
GRB2	EGR1	EGR1,GRB2	472
GRB2	EPO	EPO,EPOR,GRB2,JAK2,KIT,KITLG,PTPRC	472
GRB2	EPOR	EPO,EPOR,GRB2,KIT,KITLG,PTPRC	472
GRB2	FCGR1A	CRKL,FCGR1A,GRB2,ZAP70	472
GRB2	FGFR2	FGFR2,GRB2	472
GRB2	FLT1	CRKL,FLT1,GRB2	472
GRB2	FLT3	CRKL,FLT3,GRB2	472
GRB2	GAPDH	GAPDH,GRB2	472
GRB2	GC	GC,GRB2,TF	472
GRB2	GHR	GHR,GRB2,JAK1,JAK2,PTPRC	472
GRB2	HSPA5	GRB2,HSPA5	472
GRB2	IL2RB	GRB2,IL2RB	472
GRB2	IL4	GRB2,IL2RB,IL4,IL9R	472
GRB2	IL5RA	GRB2,IL5RA,IL9R,JAK1,JAK2	472
GRB2	IL9R	GRB2,IL2RB,IL4,IL9R	472
GRB2	INPP5D	CRKL,DOK2,GRB2,INPP5D,JAK1	472
GRB2	JAK1	GRB2,INPP5D,JAK1	472
GRB2	JAK2	GRB2,JAK2	472
GRB2	KIT	EPOR,GRB2,KIT	472
GRB2	KITLG	EPOR,FLT3,GRB2,KIT,KITLG	472
GRB2	LCK	CD4,CD86,GRB2,LCK,PTPRC	472
GRB2	LIFR	GRB2,JAK1,LIFR	472
GRB2	MAP4K1	GRB2,MAP4K1	472
GRB2	MERTK	GRB2,HSPA5,MERTK	472
GRB2	MYH11	GRB2,MYH11	472
GRB2	PREP	GRB2,PREP	472
GRB2	PTPN11	CRKL,GRB2,PTPN11	472
GRB2	PTPRC	CD4,GRB2,JAK2,PTPRC	472
GRB2	PTPRT	GRB2,PTPRT	472
GRB2	RAPGEF1	CRKL,GRB2,RAPGEF1	472
GRB2	RHOH	CD4,CRKL,GRB2,RHOH,ZAP70	472
GRB2	SOCS1	GRB2,SOCS1	472
GRB2	SYNJ1	GRB2,SYNJ1	472
GRB2	TF	GRB2,TF	472
GRB2	WNK2	GRB2,WNK2	472
GRB2	ZAP70	CD4,CRKL,GRB2,LCK,ZAP70	472

HDAC1	AFF1	AFF1,HDAC1	472
HDAC1	BCL11B	BCL11B,HDAC1,SRSF2	472
HDAC1	BCL2	BCL2,HDAC1	472
HDAC1	BCL2L11	BCL2,BCL2L11,HDAC1,MAPK8	472
HDAC1	BCL3	BCL3,HDAC1,TBL1XR1	472
HDAC1	BCOR	BCOR,HDAC1	472
HDAC1	CHFR	CHFR,HDAC1	472
HDAC1	CTCF	CTCF,HDAC1	472
HDAC1	DIABLO	DIABLO,HDAC1,PFDN4	472
HDAC1	DNMT1	DNMT1,DNMT3A,EZH2,HDAC1,SPI1	472
HDAC1	DNMT3A	DNMT3A,EZH2,HDAC1,SPI1	472
HDAC1	DNTT	DNTT,HDAC1	472
HDAC1	EGR1	EGR1,HDAC1	472
HDAC1	EZH2	EZH2,HDAC1	472
HDAC1	FLI1	FLI1,HDAC1,TP73	472
HDAC1	FOXP3	FOXP3,HDAC1	472
HDAC1	GATA2	GATA2,HDAC1,TAL1	472
HDAC1	HIF1A	HDAC1,HIF1A,IDH2,PPARG,RUNX1	472
HDAC1	HOXA9	HDAC1,HOXA9,MEIS1,PBX1	472
HDAC1	IDH2	HDAC1,HIF1A,IDH2,PPARG	472
HDAC1	KLF4	EGR1,HDAC1,KLF4,PPARG	472
HDAC1	MAP2K1	HDAC1,MAP2K1	472
HDAC1	MAPK8	HDAC1,MAPK8	472
HDAC1	MDM2	HDAC1,HOXA9,MDM2,PBX1	472
HDAC1	MEIS1	HDAC1,HOXA9,MDM2,MEIS1,PBX1	472
HDAC1	PBX1	HDAC1,MDM2,MEIS1,PBX1	472
HDAC1	PFDN4	HDAC1,PFDN4	472
HDAC1	PHF6	HDAC1,PHF6	472
HDAC1	PIK3CA	HDAC1,PIK3CA	472
HDAC1	PPARG	HDAC1,HIF1A,IDH2,PPARG	472
HDAC1	RAF1	HDAC1,RAF1	472
HDAC1	RARA	HDAC1,RARA,RUNX1	472
HDAC1	RASGRP3	HDAC1,PIK3CA,RASGRP3	472
HDAC1	RUNX1	HDAC1,HIF1A,RARA,RUNX1,RUNX1T1	472
HDAC1	RUNX1T1	HDAC1,RUNX1,RUNX1T1,ZBTB16	472
HDAC1	SALL4	EZH2,HDAC1,SALL4	472
HDAC1	SAMSN1	HDAC1,SAMSN1	472
HDAC1	SFPQ	HDAC1,SFPQ,TP73	472
HDAC1	SPI1	DNMT1,DNMT3A,EZH2,HDAC1,SPI1	472
HDAC1	SRSF2	HDAC1,SRSF2	472
HDAC1	STAT5A	HDAC1,STAT5A	472
HDAC1	TAL1	GATA2,HDAC1,TAL1	472
HDAC1	TBL1XR1	HDAC1,TBL1XR1	472

HDAC1	TOP2A	HDAC1,TOP2A,TOP2B	472
HDAC1	TOP2B	HDAC1,TOP2A,TOP2B	472
HDAC1	TP73	HDAC1,SFPQ,TP73	472
HDAC1	ZBTB16	EZH2,HDAC1,ZBTB16	472
HDAC1	ZMYM2	HDAC1,SFPQ,ZMYM2	472
HDAC1	ZMYM3	HDAC1,ZMYM3	472
HDAC1	ZNF79	DNMT1,HDAC1,RUNX1,ZNF79	472
LYN	ANGPT1	ANGPT1,ANGPT2,LYN,PTPRC	472
LYN	ANGPT2	ANGPT1,ANGPT2,LYN	472
LYN	BLNK	BLNK,BTK,CD72,LYN	472
LYN	BTK	BTK,LYN	472
LYN	CASP9	CASP9,LYN	472
LYN	CD36	CD36,CD9,LYN	472
LYN	CD72	BLNK,CD72,LYN	472
LYN	CD9	CD36,CD9,LYN	472
LYN	CDK4	CDK4,LYN	472
LYN	CSF2RB	CSF2RB,IL5RA,LYN	472
LYN	CSF3	CSF3,LYN	472
LYN	CTLA4	CTLA4,LYN	472
LYN	DHRS9	DHRS9,LYN	472
LYN	DOK1	DOK1,DOK2,INPP5D,LYN	472
LYN	DOK2	DOK2,INPP5D,LYN	472
LYN	ETV6	ETV6,LYN	472
LYN	FGR	FGR,LYN	472
LYN	FLT3	FLT3,LYN	472
LYN	GP1BA	GP1BA,LYN	472
LYN	IL1B	IL1B,LYN,PDGFRB	472
LYN	IL5RA	CSF2RB,IL5RA,LYN	472
LYN	IL6ST	IL6ST,LYN	472
LYN	IL7R	IL7R,LYN,MS4A1	472
LYN	INPP5D	DOK2,INPP5D,LYN	472
LYN	ITGB2	ITGB2,LYN	472
LYN	KITLG	CD9,FLT3,IL7R,KITLG,LYN	472
LYN	MAP4K1	LYN,MAP4K1	472
LYN	MME	LYN,MME	472
LYN	MS4A1	IL7R,LYN,MS4A1	472
LYN	PACSIN3	LYN,PACSIN3	472
LYN	PDGFRB	IL1B,LYN,PDGFRB	472
LYN	POU2F2	LYN,POU2F2	472
LYN	PRKCQ	BTK,LYN,PRKCQ	472
LYN	PTPRC	LYN,PDGFRB,PTPRC	472
SHC1	AMD1	AMD1,SHC1	472
SHC1	BCR	BCR,SHC1	472

SHC1	CSF1R	CSF1R,SHC1,TEK	472
SHC1	CSF2RB	BCR,CSF2RB,SHC1	472
SHC1	DOK1	DOK1,INPP5D,PTPN11,SHC1	472
SHC1	EGF	EGF,FGFR2,SHC1	472
SHC1	EGR1	EGR1,SHC1	472
SHC1	FCGR1A	FCGR1A,SHC1,ZAP70	472
SHC1	FCGR3A	FCGR1A,FCGR3A,SHC1,ZAP70	472
SHC1	FGFR2	FGFR2,SHC1	472
SHC1	FLT1	FLT1,KDR,SHC1	472
SHC1	FLT3	FLT3,SHC1	472
SHC1	GHR	GHR,SHC1	472
SHC1	IL2RB	IL2RB,IL2RG,SHC1	472
SHC1	IL2RG	IL2RG,IL4,IL4R,IL9R,SHC1	472
SHC1	IL4	IL2RB,IL2RG,IL4,IL4R,IL9R,SHC1	472
SHC1	IL4R	IL2RG,IL4,IL4R,SHC1	472
SHC1	IL6ST	IL6ST,SHC1	472
SHC1	IL9R	IL2RB,IL2RG,IL4,IL9R,SHC1	472
SHC1	INPP5D	INPP5D,SHC1	472
SHC1	IRAK1	IRAK1,SHC1	472
SHC1	KDR	FLT1,KDR,SHC1	472
SHC1	MAPKAPK2	MAPKAPK2,SHC1	472
SHC1	MAT1A	MAT1A,SHC1	472
SHC1	MPL	MPL,SHC1	472
SHC1	MYH11	MYH11,SHC1	472
SHC1	PTPN11	PTPN11,SHC1	472
SHC1	RAPGEF1	RAPGEF1,SHC1	472
SHC1	RET	RET,SHC1	472
SHC1	TEK	CSF1R,SHC1,TEK	472
SHC1	ZAP70	SHC1,ZAP70	472
STAT1	BCL3	BCL3,STAT1	472
STAT1	CCR1	CCR1,STAT1	472
STAT1	CCR5	CCR5,CXCR4,EGFR,JAK2,LCK,STAT1	472
STAT1	CD40	CD40,STAT1	472
STAT1	CSF2RB	CSF2RB,JAK2,KIT,STAT1,SYK	472
STAT1	CXCR4	CXCR4,STAT1	472
STAT1	CYBB	CYBB,STAT1,TNFRSF1A	472
STAT1	E2F1	E2F1,STAT1	472
STAT1	EGFR	EGFR,STAT1	472
STAT1	FLT1	FLT1,KDR,STAT1	472
STAT1	FOXP3	FOXP3,STAT1	472
STAT1	GATA3	GATA3,MAPK14,STAT1,TBX21	472
STAT1	GFAP	GFAP,STAT1	472
STAT1	HSPA4L	HSPA4L,STAT1	472

STAT1	IFNAR2	IFNAR2,ISG15,STAT1	472
STAT1	IFNG	IFNG,JAK2,STAT1	472
STAT1	IL2RG	IL2RG,JAK2,KIT,STAT1	472
STAT1	IL6ST	EGFR,IL6ST,JAK2,STAT1	472
STAT1	IRF8	IRF8,ISG15,PTPN11,STAT1	472
STAT1	ISG15	ISG15,STAT1	472
STAT1	JAK2	EGFR,JAK2,NFKBIA,STAT1	472
STAT1	KDR	FLT1,KDR,STAT1	472
STAT1	KIT	KIT,STAT1	472
STAT1	LCK	LCK,PIK3CA,STAT1	472
STAT1	LIF	IL6ST,LIF,STAT1	472
STAT1	MAPK14	MAPK14,PML,STAT1	472
STAT1	NFKBIA	JAK2,NFKBIA,STAT1	472
STAT1	NOS2	NOS2,STAT1	472
STAT1	PDGFRB	PDGFRB,STAT1	472
STAT1	PIK3CA	PIK3CA,STAT1	472
STAT1	PML	PML,STAT1	472
STAT1	POU2F2	POU2F2,STAT1	472
STAT1	PSMC3	PSMC3,STAT1,XPO1	472
STAT1	PTPN11	PTPN11,STAT1	472
STAT1	STAT5A	STAT1,STAT5A	472
STAT1	SYK	STAT1,SYK	472
STAT1	TBX21	GATA3,MAPK14,STAT1,TBX21	472
STAT1	TLR7	STAT1,TLR7,XPO1	472
STAT1	TNFRSF1A	EGFR,STAT1,TNFRSF1A	472
STAT1	XPO1	PSMC3,STAT1,XPO1	472
XRCC5	APEX1	APEX1,XRCC5	472
XRCC5	BAX	BAX,XRCC5	472
XRCC5	BIRC3	BIRC3,MSH2,MSH6,XRCC5	472
XRCC5	BRCA1	BRCA1,MSH2,XRCC5	472
XRCC5	CDK2	CDK2,MSH2,XRCC5	472
XRCC5	CFTR	CFTR,XRCC5	472
XRCC5	CHEK2	BRCA1,CHEK2,MSH2,XRCC5	472
XRCC5	DLD	DLD,XRCC5	472
XRCC5	EED	EED,XRCC5	472
XRCC5	EP300	EP300,JUN,XRCC5	472
XRCC5	EWSR1	EWSR1,ITGA4,JUN,XRCC5	472
XRCC5	FLI1	BRCA1,EWSR1,FLI1,XRCC5	472
XRCC5	HSPA4	CFTR,HSPA4,JUN,XRCC5	472
XRCC5	HSPB1	EP300,HSPB1,XRCC5	472
XRCC5	ICAM1	CFTR,ICAM1,XRCC5	472
XRCC5	ISG15	ISG15,XRCC5	472
XRCC5	ITGA4	ITGA4,XRCC5	472

XRCC5	JUN	EP300,JUN,XRCC5	472
XRCC5	JUP	JUP,MSH2,XRCC5	472
XRCC5	MAPK8	JUN,MAPK8,XRCC5	472
XRCC5	MME	MME,PCNA,XRCC5	472
XRCC5	MSH2	BRCA1,CHEK2,JUN,MSH2,MSH6,XRCC5	472
XRCC5	MSH6	BRCA1,MSH2,MSH6,MYC,PCNA,XRCC5	472
XRCC5	MYC	JUN,MSH2,MYC,XRCC5	472
XRCC5	NFKB2	NFKB2,XRCC5	472
XRCC5	NOS2	HSPB1,JUN,NOS2,XRCC5	472
XRCC5	NR4A1	CHEK2,MAPK8,NR4A1,XRCC5	472
XRCC5	PARP1	JUN,MYC,PARP1,XRCC5	472
XRCC5	PCNA	BRCA1,EP300,PCNA,XRCC5	472
XRCC5	PTEN	PTEN,XRCC5	472
XRCC5	PTPRT	PTPRT,XRCC5	472
XRCC5	RFC1	BRCA1,MSH2,MSH6,MYC,PCNA,RFC1,XRCC5	472
XRCC5	SUZ12	SUZ12,XRCC5	472
XRCC5	TERT	MYC,TERT,XRCC5	472
XRCC5	TOP2A	EP300,MYC,TOP2A,TOP2B,XRCC5	472
XRCC5	TOP2B	EP300,JUN,MSH2,TOP2A,TOP2B,XRCC5	472

Sample 546

**Candidate AML genes**	**Known AML genes** (of which the module includes candidate AML genes)	**ALL the AML genes involved in current module**	**Sample-ID**
HDAC1	AFF1	AFF1,HDAC1	546
HDAC1	ANPEP	ANPEP,APEX1,HDAC1,JUN,TP53	546
HDAC1	APEX1	APEX1,HDAC1,JUN,PCNA,TP53	546
HDAC1	ATM	ATM,HDAC1,TP53	546
HDAC1	BCL11B	BCL11B,HDAC1	546
HDAC1	BCL3	BCL3,HDAC1,JUN,NFKB2,TBL1XR1	546
HDAC1	CCND1	CCND1,HDAC1,JUN,PCNA,RUNX1,TP53	546
HDAC1	CDCA7L	CDCA7L,HDAC1,HOXA9,PBX1	546
HDAC1	CDKN1A	CDKN1A,HDAC1,JUN	546
HDAC1	CEACAM5	CEACAM5,HDAC1	546
HDAC1	CEBPB	CEBPB,HDAC1,HDAC9,JUN,SYK,TP53	546
HDAC1	CHFR	CHFR,HDAC1,PCNA,PML	546
HDAC1	CR2	CR2,HDAC1	546
HDAC1	DAXX	ATM,DAXX,HDAC1,JUN,PML,TP53	546
HDAC1	DNMT1	DNMT1,HDAC1,PCNA	546
HDAC1	DNMT3A	DAXX,DNMT1,DNMT3A,HDAC1,SPI1	546
HDAC1	E2F1	ATM,E2F1,HDAC1,TP53	546

HDAC1	EGR1	CEBPB,EGR1,HDAC1,JUN,TP53	546
HDAC1	ELL	ELL,HDAC1,TP53	546
HDAC1	FOXP3	FOXP3,HDAC1	546
HDAC1	GATA2	GATA2,HDAC1,JUN,TAL1,ZBTB16	546
HDAC1	GATA3	GATA2,GATA3,HDAC1,RUNX1,TAL1	546
HDAC1	HBG2	HBG2,HDAC1	546
HDAC1	HDAC9	HDAC1,HDAC9,JUN,MAPK14	546
HDAC1	HOXA9	HDAC1,HOXA9,JUN,MEIS1,PBX1	546
HDAC1	HSPA8	HDAC1,HSPA8	546
HDAC1	HSPD1	HDAC1,HSPD1	546
HDAC1	IDH2	HDAC1,IDH2,JUN	546
HDAC1	JUN	CEBPB,HDAC1,HDAC9,JUN,SYK,TP53	546
HDAC1	KLF4	EGR1,HDAC1,KLF4,PPARG,TP53	546
HDAC1	MAPK14	ATM,HDAC1,JUN,MAPK14,TP53	546
HDAC1	MECOM	HDAC1,JUN,MECOM,TP53	546
HDAC1	MEIS1	CDCA7L,HDAC1,HOXA9,MEIS1,PBX1	546
HDAC1	MYB	CEBPB,GATA3,HDAC1,MYB,TAL1	546
HDAC1	NFKB2	HDAC1,HSPA8,NFKB2,NFKBIA	546
HDAC1	NFKBIA	HDAC1,HSPA8,JUN,NFKBIA	546
HDAC1	NR3C1	HDAC1,JUN,NR3C1,PBX1,TP53	546
HDAC1	PBX1	CDCA7L,HDAC1,HOXA9,PBX1	546
HDAC1	PCNA	HDAC1,JUN,PCNA	546
HDAC1	PFDN4	HDAC1,PFDN4	546
HDAC1	PHF6	HDAC1,JUN,PHF6	546
HDAC1	PKIA	CCND1,HDAC1,HSPA8,PKIA	546
HDAC1	PML	HDAC1,PML,RUNX1	546
HDAC1	PPARG	E2F1,HDAC1,JUN,PPARG,TP53	546
HDAC1	RAF1	HDAC1,RAF1	546
HDAC1	RARA	E2F1,HDAC1,HSPA8,JUN,PML,RARA,RUNX1,<br>ZBTB16	546
HDAC1	RUNX1	GATA3,HDAC1,JUN,RUNX1,RUNX1T1	546
HDAC1	RUNX1T1	HDAC1,RUNX1,RUNX1T1,SPI1,TP53	546
HDAC1	SALL4	CDKN1A,HDAC1,SALL4	546
HDAC1	SAMSN1	HDAC1,SAMSN1	546
HDAC1	SETD2	DNMT1,HDAC1,SETD2,TP53	546
HDAC1	SFPQ	HDAC1,JUN,SFPQ,ZMYM2	546
HDAC1	SPI1	CEBPB,GATA3,HDAC1,JUN,PML,SPI1	546
HDAC1	STAT5A	HDAC1,STAT5A	546
HDAC1	SYK	HDAC1,HDAC9,JUN,SYK,TP53	546
HDAC1	TAL1	GATA2,GATA3,HDAC1,TAL1	546
HDAC1	TBL1XR1	HDAC1,TBL1XR1	546
HDAC1	TOP2A	HDAC1,TOP2A,TOP2B,TP53	546
HDAC1	TOP2B	ATM,HDAC1,JUN,TOP2A,TOP2B	546

HDAC1	TP53	CEBPB,HDAC1,HDAC9,JUN,SYK,TP53	546
HDAC1	ZBTB16	HDAC1,HDAC9,JUN,SYK,TP53,ZBTB16	546
HDAC1	ZMYM2	HDAC1,SFPQ,ZMYM2	546
HDAC1	ZNF79	HDAC1,ZNF79	546
GRB2	ABL1	ABL1,CRKL,GRB2,JAK1	546
GRB2	ASXL1	ASXL1,GRB2	546
GRB2	BCR	ABL1,BCR,CRKL,GRB2,PIK3R1,PTPN11	546
GRB2	BLNK	BLNK,CD72,GRB2	546
GRB2	CCR5	CCR5,DNM2,GRB2,LCK	546
GRB2	CD22	CD22,GRB2	546
GRB2	CD72	BLNK,CD72,GRB2,ZAP70	546
GRB2	CD86	CD86,GRB2,LCK,PTPN11	546
GRB2	CDKN1B	CDKN1B,GRB2	546
GRB2	CGA	CGA,GRB2,HSPB1	546
GRB2	CRKL	CRKL,GRB2,PIK3R1	546
GRB2	CSF1R	CSF1R,GRB2	546
GRB2	CSF3	CSF3,GRB2,JAK1	546
GRB2	CXCR4	CXCR4,GRB2	546
GRB2	DLX4	ABL1,DLX4,GRB2	546
GRB2	DNM2	DNM2,GRB2,MKI67	546
GRB2	DOK1	ABL1,CRKL,DOK1,GRB2,PIK3R1,PTPN11	546
GRB2	EGF	EGF,GRB2	546
GRB2	EGR1	EGR1,GRB2	546
GRB2	EPOR	CD22,EPOR,GRB2,KIT,KITLG	546
GRB2	ETV6	ETV6,GRB2	546
GRB2	FCGR1A	CRKL,FCGR1A,GRB2,PIK3R1,ZAP70	546
GRB2	FGFR1	CRKL,FGFR1,GRB2,PIK3R1	546
GRB2	FGFR2	FGFR1,FGFR2,GRB2,PIK3R1	546
GRB2	FLT1	FLT1,GRB2	546
GRB2	FLT3	ABL1,FLT3,GRB2,KIT	546
GRB2	GC	GC,GRB2,TF	546
GRB2	GHR	GHR,GRB2,JAK1,PDGFRB	546
GRB2	HSPB1	GRB2,HSPB1,LCK	546
GRB2	IL2RB	GRB2,IL2RB,IL7R,IL9R	546
GRB2	IL5RA	GRB2,IL5RA,ILK,JAK1	546
GRB2	IL7R	GRB2,IL2RB,IL7R,IL9R	546
GRB2	IL9R	GRB2,IL2RB,IL7R,IL9R	546
GRB2	ILK	GRB2,ILK,LCK,ZAP70	546
GRB2	INPP5D	CRKL,GRB2,INPP5D,JAK1	546
GRB2	JAK1	ABL1,GRB2,JAK1,PIK3R1,PTPN11	546
GRB2	KIT	BLNK,GRB2,KIT,PDGFRB,PIK3R1	546
GRB2	KITLG	CSF1R,EPOR,FLT3,GRB2,KIT,KITLG	546
GRB2	LCK	GRB2,ILK,LCK,ZAP70	546

GRB2	LIF	GRB2,JAK1,LIF,LIFR,PIK3R1	546
GRB2	LIFR	GRB2,LIF,LIFR	546
GRB2	MAP4K1	GRB2,MAP4K1,PIK3R1	546
GRB2	MERTK	GRB2,MERTK	546
GRB2	MKI67	DNM2,GRB2,MKI67	546
GRB2	MPL	EPOR,GRB2,MPL,RET	546
GRB2	MYH11	GRB2,MYH11	546
GRB2	MYO1G	GRB2,MYO1G	546
GRB2	NOS2	GRB2,NOS2	546
GRB2	PDGFRB	BLNK,GRB2,KIT,PDGFRB,PIK3R1	546
GRB2	PIK3R1	GRB2,KIT,PDGFRB,PIK3R1	546
GRB2	PTPN11	CRKL,GRB2,JAK1,LCK,PIK3R1,PTPN11	546
GRB2	RAPGEF1	ABL1,CRKL,GRB2,PIK3R1,PTPN11,RAPGEF1	546
GRB2	RET	CRKL,GRB2,PIK3R1,RET	546
GRB2	RHOH	CRKL,GRB2,LCK,PIK3R1,RHOH,ZAP70	546
GRB2	SALL4	GRB2,SALL4	546
GRB2	SYNJ1	GRB2,SYNJ1	546
GRB2	TF	GRB2,TF	546
GRB2	TYMS	GRB2,HSPB1,TYMS	546
GRB2	VANGL1	GRB2,VANGL1	546
GRB2	WNK2	GRB2,WNK2	546
GRB2	ZAP70	FCGR1A,GRB2,ILK,LCK,ZAP70	546
FYN	BAD	BAD,FYN	546
FYN	BCL2L1	BCL2L1,FYN,PTPRC	546
FYN	BTK	BTK,FYN,JAK1	546
FYN	CASP3	CASP3,FYN	546
FYN	CASP8	BCL2L1,CASP8,FYN	546
FYN	CASP9	BCL2L1,CASP9,FYN,RASA1	546
FYN	CD14	CD14,FYN	546
FYN	CD19	CD19,FYN	546
FYN	CD22	BTK,CD19,CD22,FYN,PTPRC	546
FYN	CD5	CD5,CD58,FYN,PTPRC	546
FYN	CD58	CD5,CD58,DOK1,FYN,PTPRC	546
FYN	CD83	CD83,FYN	546
FYN	CD86	CD86,CTLA4,FYN,PTPN11,PTPRC	546
FYN	CLTC	CLTC,FYN,TFRC	546
FYN	CSF2RB	CSF2RB,FYN,JAK1	546
FYN	CTLA4	CD86,CTLA4,FYN,JAK1	546
FYN	CTNNB1	CTNNB1,FYN	546
FYN	DNM2	DNM2,FYN,MKI67	546
FYN	DOK1	DOK1,DOK2,FYN,PTPN11,RASA1	546
FYN	DOK2	DOK2,FYN,RASA1	546
FYN	EPM2A	DNM2,EPM2A,FYN,JAK1	546

FYN	FAS	FAS,FYN	546
FYN	FCGR1A	FCGR1A,FYN	546
FYN	FGFR2	FGFR2,FYN	546
FYN	FLT1	FLT1,FYN	546
FYN	GUCY2D	FYN,GUCY2D	546
FYN	ICAM1	CTNNB1,FYN,ICAM1,RPL5	546
FYN	IL18R1	CASP3,FYN,IL18R1	546
FYN	IL7	FYN,IL7,IL7R,JAK1,SOCS1	546
FYN	IL7R	FYN,ICAM1,IL7,IL7R	546
FYN	JAK1	FYN,JAK1,PTPN11	546
FYN	LIFR	FYN,LIFR	546
FYN	MAP4K1	CASP3,FYN,MAP4K1	546
FYN	MAPK8	FYN,MAPK8,PTPRC	546
FYN	MAT2A	FYN,MAT2A	546
FYN	MERTK	FYN,MERTK	546
FYN	MKI67	DNM2,FYN,MKI67	546
FYN	MPL	FYN,MPL,PTPRC	546
FYN	MS4A1	FYN,IL7R,MS4A1	546
FYN	PRDX2	FYN,PRDX2	546
FYN	PTPN11	FYN,JAK1,PTPN11	546
FYN	PTPRC	FYN,PTPRC,RASA1	546
FYN	RAPGEF1	FYN,PTPN11,RAPGEF1	546
FYN	RASA1	CASP3,FYN,RASA1,RPL5	546
FYN	RPL5	CASP3,FYN,RASA1,RPL5	546
FYN	SOCS1	FYN,SOCS1	546
FYN	TFRC	FYN,TFRC	546
FYN	TG	FYN,TG	546
FYN	TNFRSF10B	FYN,TNFRSF10B	546
SHC1	ABL1	ABL1,CRKL,SHC1	546
SHC1	AMD1	AMD1,HSPB1,SHC1	546
SHC1	BCR	ABL1,BCR,CRKL,PIK3R1,PTPN11,SHC1	546
SHC1	CRKL	CRKL,PIK3R1,SHC1	546
SHC1	CSF2RB	BCR,CSF2RB,KIT,PIK3R1,SHC1	546
SHC1	DOK1	ABL1,CRKL,DOK1,DOK2,PIK3R1,PTPN11,SHC1	546
SHC1	DOK2	ABL1,CRKL,DOK2,SHC1	546
SHC1	EGR1	EGR1,SHC1	546
SHC1	FCGR1A	CRKL,FCGR1A,PIK3R1,SHC1	546
SHC1	FCGR3A	FCGR1A,FCGR3A,SHC1	546
SHC1	FGFR1	CRKL,FGFR1,PIK3R1,SHC1	546
SHC1	FGFR2	FGFR1,FGFR2,PIK3R1,SHC1	546
SHC1	FLT1	FLT1,SHC1	546
SHC1	FLT3	ABL1,FLT3,KIT,SHC1	546

SHC1	GHR	GHR,PDGFRB,SHC1	546
SHC1	HSPB1	HSPB1,SHC1	546
SHC1	IGF1R	IGF1R,SHC1	546
SHC1	IL2RB	IL2RB,IL4R,IL7R,IL9R,SHC1	546
SHC1	IL4R	IL4R,SHC1	546
SHC1	IL7R	IL2RB,IL7R,IL9R,SHC1	546
SHC1	IL9R	IL2RB,IL4R,IL7R,IL9R,SHC1	546
SHC1	INPP5D	CRKL,DOK2,INPP5D,SHC1	546
SHC1	KIT	KIT,PDGFRB,PIK3R1,SHC1	546
SHC1	KITLG	FLT3,KIT,KITLG,SHC1	546
SHC1	MAPK14	MAPK14,SHC1	546
SHC1	MAPKAPK2	MAPK14,MAPKAPK2,SHC1	546
SHC1	MAT1A	MAT1A,SHC1	546
SHC1	MPL	FCGR3A,IGF1R,MPL,RET,SHC1	546
SHC1	MYH11	MYH11,SHC1	546
SHC1	NF1	NF1,SHC1	546
SHC1	PDGFRB	KIT,PDGFRB,PIK3R1,SHC1	546
SHC1	PIK3R1	KIT,PDGFRB,PIK3R1,SHC1	546
SHC1	PRDX2	PRDX2,SHC1	546
SHC1	PTPN11	CRKL,MAPK14,PIK3R1,PTPN11,SHC1,STAT5A	546
SHC1	RAPGEF1	ABL1,CRKL,PIK3R1,PTPN11,RAPGEF1,SHC1	546
SHC1	RB1	RB1,SHC1	546
SHC1	RET	CRKL,IGF1R,PIK3R1,RET,SHC1	546
SHC1	STAT5A	CRKL,PTPN11,SHC1,STAT5A	546
ESR1	BCL3	BCL3,ESR1	546
ESR1	BIRC5	BIRC5,ESR1	546
ESR1	CAV1	CAV1,CBL,CTNNB1,ESR1,KRAS	546
ESR1	CBL	CAV1,CBL,CTNNB1,ESR1,KRAS	546
ESR1	CHD2	CHD2,ESR1,EZH2	546
ESR1	CNTN2	CNTN2,ESR1,NCAM1	546
ESR1	CTLA4	CTLA4,ESR1	546
ESR1	CTNNB1	CTNNB1,ERBB4,ESR1	546
ESR1	ELF4	ELF4,ESR1	546
ESR1	ERBB4	ERBB4,ESR1	546
ESR1	EZH2	ESR1,EZH2,SUZ12	546
ESR1	GP1BA	ESR1,GP1BA,PIK3R1	546
ESR1	HBA2	ESR1,HBA2,HBB	546
ESR1	HBB	ESR1,HBA2,HBB	546
ESR1	IL5RA	ESR1,IL5RA,ILK	546
ESR1	ILK	ERBB4,ESR1,ILK	546
ESR1	JUP	CAV1,CTNNB1,ESR1,JUP,KRAS	546
ESR1	KIT	ESR1,KIT,PDGFRB,PIK3R1	546
ESR1	KMT2C	ESR1,EZH2,KMT2C	546

ESR1	KRAS	CAV1,CBL,CTNNB1,ESR1,KRAS	546
ESR1	MAP2K1	CAV1,CBL,ESR1,KRAS,MAP2K1	546
ESR1	MVP	ESR1,MVP	546
ESR1	NCAM1	CNTN2,ESR1,NCAM1	546
ESR1	NPPA	ESR1,NPPA	546
ESR1	PDGFRB	ESR1,KIT,PDGFRB,PIK3R1	546
ESR1	PIK3R1	ESR1,KIT,PDGFRB,PIK3R1	546
ESR1	PKIA	ESR1,PKIA	546
ESR1	PPP2R3A	ESR1,PPP2R3A	546
ESR1	PSMC3	ESR1,PSMC3	546
ESR1	PTHLH	ESR1,PTHLH	546
ESR1	PTPRT	ESR1,PTPRT	546
ESR1	ROS1	CTNNB1,ESR1,ROS1	546
ESR1	SAMSN1	ESR1,SAMSN1	546
ESR1	SUZ12	ESR1,EZH2,SUZ12	546
ESR1	TERT	ESR1,TERT	546
ESR1	TG	ESR1,TG	546
ESR1	TYMS	ESR1,TYMS	546
ESR1	ZNF79	ESR1,ZNF79	546
LYN	ANGPT1	ANGPT1,LYN	546
LYN	BLNK	BLNK,CD72,LYN	546
LYN	BTK	BTK,LYN	546
LYN	CASP2	CASP2,LYN	546
LYN	CASP9	CASP9,LYN	546
LYN	CD36	CD36,LYN	546
LYN	CD72	BLNK,CD72,LYN,PTPRC	546
LYN	CD86	CD86,LCK,LYN,PTPRC	546
LYN	CDK1	CDK1,LYN	546
LYN	CRKL	CRKL,LYN	546
LYN	CSF2RB	CSF2RB,IL5RA,LYN	546
LYN	CSF3	CSF3,LYN	546
LYN	CSPG4	CSPG4,LYN	546
LYN	DOK1	CRKL,DOK1,DOK2,LYN	546
LYN	DOK2	CRKL,DOK2,LYN	546
LYN	EPM2A	EPM2A,LYN	546
LYN	FCER2	FCER2,ITGB2,LYN	546
LYN	FGFR2	FGFR2,LYN	546
LYN	FGR	FGR,LYN	546
LYN	FHIT	FHIT,LYN	546
LYN	FLT3	FLT3,LYN	546
LYN	GP1BA	GP1BA,LCK,LYN	546
LYN	IL5RA	IL5RA,LYN	546
LYN	IL6R	IL6R,LYN	546

LYN	INPP5D	CRKL,DOK2,INPP5D,LYN	546
LYN	ITGA4	CD36,ITGA4,LYN	546
LYN	ITGB2	CDK1,FCER2,ITGB2,LYN,PTPRC	546
LYN	LCK	LCK,LYN	546
LYN	LIF	IL6R,LIF,LYN	546
LYN	MME	LYN,MME	546
LYN	MUC1	LYN,MUC1	546
LYN	PARP1	LYN,PARP1,RXRA	546
LYN	POU2F2	LYN,POU2F2,RXRA	546
LYN	PTPRC	LCK,LYN,PTPRC	546
LYN	RXRA	LYN,PARP1,RXRA	546
RELA	AATF	AATF,RELA	546
RELA	ACTBL2	ACTBL2,RELA	546
RELA	AFF1	AFF1,RELA,VDR	546
RELA	BCL3	BCL3,RELA	546
RELA	CCND2	CCND2,PSMC3,RELA	546
RELA	CDX2	CDX2,RELA	546
RELA	CHFR	CHFR,PARP1,RELA	546
RELA	CYP24A1	CYP24A1,RELA	546
RELA	EGF	EGF,RELA	546
RELA	ELF4	ELF4,NOTCH1,PPARGC1A,RELA	546
RELA	FOXP3	FOXP3,RELA	546
RELA	HLF	HLF,RELA	546
RELA	IL1B	IL1B,IRF4,IRF8,RELA	546
RELA	IRF4	IRF4,RELA,STAT6	546
RELA	IRF8	IL1B,IRF8,RELA	546
RELA	KLF4	KLF4,RELA	546
RELA	MAP4K1	MAP4K1,RELA	546
RELA	NCOA6	NCOA6,RELA	546
RELA	NFKBIB	NFKBIB,RELA	546
RELA	NOTCH1	NOTCH1,RELA	546
RELA	NR4A1	NR4A1,RELA	546
RELA	PARP1	PARP1,PBX1,RELA,RXRA,VDR	546
RELA	PBX1	PARP1,PBX1,RELA,RXRA	546
RELA	PGR	PARP1,PGR,RELA,STAT5A	546
RELA	PLEKHM1	PLEKHM1,RELA	546
RELA	PPARGC1A	PPARGC1A,RELA	546
RELA	PSMC3	PSMC3,RELA	546
RELA	RUNX1T1	RELA,RUNX1T1	546
RELA	RXRA	PARP1,PBX1,RELA,RXRA,VDR	546
RELA	STAT5A	RELA,STAT5A	546
RELA	STAT6	IRF4,RELA,STAT6	546
RELA	TF	RELA,TF	546

RELA	VDR	PARP1,PBX1,RELA,RXRA,VDR	546
ESR2	AFF1	AFF1,ESR2,MLLT3	546
ESR2	ATM	ATM,ESR2	546
ESR2	BAX	BAX,ESR2	546
ESR2	BCL11B	BCL11B,ESR2	546
ESR2	BIRC5	BIRC5,ESR2	546
ESR2	CCR1	CCR1,ESR2,STAT3	546
ESR2	CD19	CD19,ESR2	546
ESR2	CD8A	CD8A,ESR2	546
ESR2	CDCA7L	CDCA7L,ESR2,PBX1	546
ESR2	CHD2	CHD2,ESR2	546
ESR2	CNR2	CNR2,ESR2	546
ESR2	CTCF	CTCF,ESR2	546
ESR2	EDNRA	EDNRA,ESR2	546
ESR2	ELL	ELL,ESR2	546
ESR2	ETV6	ESR2,ETV6	546
ESR2	HBA2	ESR2,HBA2	546
ESR2	ICOS	ESR2,ICOS	546
ESR2	IL6	ESR2,IL6,STAT3	546
ESR2	KMT2C	ESR2,KMT2C	546
ESR2	MEIS1	CDCA7L,ESR2,MEIS1,PBX1	546
ESR2	MLLT3	ESR2,MLLT3	546
ESR2	MSH2	ATM,ESR2,MSH2,STAT3	546
ESR2	NELFCD	ESR2,NELFCD	546
ESR2	PBX1	CDCA7L,ESR2,PBX1	546
ESR2	PPARGC1A	ESR2,PPARGC1A	546
ESR2	ROS1	ESR2,ROS1	546
ESR2	S100A11	ESR2,S100A11,S100A8	546
ESR2	S100A8	ESR2,S100A8	546
ESR2	STAT3	ESR2,STAT3	546
ESR2	STAT5A	ESR2,STAT3,STAT5A	546
ESR2	TCF15	ESR2,TCF15	546
ESR2	WTAP	ESR2,WTAP	546
ITGB1	ANGPT1	ANGPT1,ANGPT2,CD9,ITGA5,ITGB1	546
ITGB1	ANGPT2	ANGPT1,ANGPT2,DOK1,ITGA5,ITGB1	546
ITGB1	CAV1	CAV1,CBL,ITGB1,KRAS	546
ITGB1	CBL	CAV1,CBL,ITGB1,KRAS	546
ITGB1	CD2	CD2,CD58,ITGB1	546
ITGB1	CD36	CD36,CD9,ITGB1	546
ITGB1	CD58	CD2,CD58,DOK1,ITGB1	546
ITGB1	CD9	CD36,CD9,ITGB1,KRAS	546
ITGB1	CNR1	CNR1,ITGB1	546
ITGB1	CSF2RB	CSF2RB,ITGB1,JAK2	546

ITGB1	CSPG4	CSPG4,ITGA5,ITGB1	546
ITGB1	CXCL10	CXCL10,ITGB1	546
ITGB1	CYP24A1	CYP24A1,ITGB1	546
ITGB1	DOK1	DOK1,ITGB1	546
ITGB1	FCER2	FCER2,ITGAX,ITGB1,ITGB2	546
ITGB1	HBEGF	CAV1,CD9,HBEGF,ITGA4,ITGB1	546
ITGB1	ITGA4	CD36,ITGA4,ITGB1	546
ITGB1	ITGA5	CBL,CD9,ITGA5,ITGB1,KRAS	546
ITGB1	ITGAX	FCER2,ITGAX,ITGB1,ITGB2,PTK2B	546
ITGB1	ITGB2	FCER2,ITGAX,ITGB1,ITGB2	546
ITGB1	JAK2	CAV1,ITGB1,JAK2,KRAS	546
ITGB1	JUP	CAV1,ITGB1,JUP,KRAS	546
ITGB1	KRAS	CAV1,CBL,ITGB1,KRAS	546
ITGB1	MAP2K1	CAV1,CBL,ITGB1,KRAS,MAP2K1	546
ITGB1	MPL	ITGB1,JAK2,MPL	546
ITGB1	PICALM	ITGB1,PICALM	546
ITGB1	PLXNA2	ITGA5,ITGB1,PLXNA2	546
ITGB1	PTK2B	ITGB1,PTK2B	546
ITGB1	SDC1	CD9,ITGB1,SDC1	546
ITGB1	SLC22A2	CD9,ITGB1,SLC22A2	546
ITGB1	TNFRSF1B	ITGB1,TNFRSF1B	546
ITGB1	TNFSF13B	ITGB1,TNFSF13B	546
TRAF6	BIRC2	BIRC2,TRAF6	546
TRAF6	BIRC3	BIRC3,NFKB1,RAN,TRAF6	546
TRAF6	CARD11	CARD11,TRAF6	546
TRAF6	CD40	BIRC2,CD40,PIK3R1,TRAF6	546
TRAF6	CSF2RB	CSF2RB,KIT,PIK3R1,TRAF6	546
TRAF6	DCT	DCT,TRAF6	546
TRAF6	EPO	EPO,TRAF6	546
TRAF6	GUCY2D	GUCY2D,PIK3R1,TRAF6	546
TRAF6	IRAK1	IRAK1,MYD88,TRAF6	546
TRAF6	IRF8	IRF8,NFKB1,PTPN11,TRAF6	546
TRAF6	KIT	KIT,PDGFRB,PIK3R1,TRAF6	546
TRAF6	LTA	BIRC2,LTA,TRAF6	546
TRAF6	MAT1A	MAT1A,MAT2A,TRAF6	546
TRAF6	MAT2A	MAT1A,MAT2A,TRAF6	546
TRAF6	MBP	IRAK1,MBP,TRAF6	546
TRAF6	MPL	MPL,TRAF6	546
TRAF6	MVP	MVP,PARP4,TRAF6	546
TRAF6	MYD88	IRAK1,MYD88,TRAF6	546
TRAF6	MYO1G	MYO1G,NFKB1,TFRC,TRAF6	546
TRAF6	NFKB1	NFKB1,RAN,TRAF6	546
TRAF6	PARP4	MVP,PARP4,TRAF6	546

TRAF6	PDGFRB	KIT,PDGFRB,PIK3R1,TRAF6	546
TRAF6	PIK3R1	KIT,PDGFRB,PIK3R1,TRAF6	546
TRAF6	PTPN11	PIK3R1,PTPN11,TRAF6	546
TRAF6	RAN	NFKB1,RAN,TRAF6	546
TRAF6	RASGRP3	RASGRP3,TRAF6	546
TRAF6	SIGLEC9	BIRC2,MYD88,SIGLEC9,TRAF6	546
TRAF6	ST13	IRAK1,ST13,TRAF6	546
TRAF6	TFRC	TFRC,TRAF6	546
TRAF6	WBP1L	RAN,TRAF6,WBP1L	546
TRAF6	WNT5A	TRAF6,WNT5A	546
PRKCA	ACTBL2	ACTBL2,PRKCA	546
PRKCA	BCL2L1	BCL2L1,MAPK1,PRKCA	546
PRKCA	CAD	CAD,PRKCA	546
PRKCA	CD4	CD4,PRKCA,PRKCQ	546
PRKCA	CD58	CD4,CD58,PRKCA	546
PRKCA	CEACAM1	CEACAM1,EGFR,PRKCA	546
PRKCA	EGFR	EGFR,PRKCA,SRC	546
PRKCA	F2RL1	F2RL1,GNAS,PRKCA	546
PRKCA	FCGR3A	CD4,FCGR3A,PRKCA	546
PRKCA	GC	GC,PRKCA	546
PRKCA	GNAS	F2RL1,GNAS,PRKCA	546
PRKCA	HRAS	HRAS,MAPK1,MAPK3,PRKCA	546
PRKCA	HSPB1	BCL2L1,HSPB1,PRKCA	546
PRKCA	LMOD1	LMOD1,PRKCA	546
PRKCA	MAPK1	HRAS,MAPK1,MAPK3,PRKCA	546
PRKCA	MAPK3	HRAS,MAPK1,MAPK3,PRKCA	546
PRKCA	MAPK8	MAPK1,MAPK8,PRKCA	546
PRKCA	MBP	HRAS,MAPK1,MAPK3,MBP,PRKCA,RAF1	546
PRKCA	MRC2	MRC2,PRKCA,RAF1	546
PRKCA	MUSK	MAPK1,MUSK,PRKCA	546
PRKCA	PIK3CA	PIK3CA,PRKCA,SRC	546
PRKCA	PIM1	EGFR,PIM1,PRKCA	546
PRKCA	PRKCQ	PRKCA,PRKCQ	546
PRKCA	RAF1	PRKCA,RAF1	546
PRKCA	SI	PRKCA,SI	546
PRKCA	SLC22A2	PRKCA,SLC22A2	546
PRKCA	SLC8A1	PRKCA,SLC8A1	546
PRKCA	SRC	PRKCA,SRC	546
PRKCA	STX4	HRAS,PRKCA,STX4,VAMP2	546
PRKCA	VAMP2	HRAS,PRKCA,STX4,VAMP2	546

Sample 629

**Candidate AML genes**	**Known AML genes** (of which the module includes candidate AML genes)	**ALL the AML genes involved in current module**	**Sample-ID**
ESR1	ANKRD49	ANKRD49,ESR1	629
ESR1	BCL3	BCL3,ESR1	629
ESR1	CAD	CAD,ESR1	629
ESR1	CHD2	CHD2,ESR1	629
ESR1	CNTN2	CNTN2,ESR1,NCAM1	629
ESR1	DCT	DCT,ESR1	629
ESR1	DNTT	DNTT,ESR1	629
ESR1	ECT2L	ECT2L,ESR1	629
ESR1	EGF	EGF,ESR1	629
ESR1	HIF1A	ESR1,HIF1A	629
ESR1	HNF4A	ESR1,HNF4A,NR3C1,PGR,RORA	629
ESR1	HSPA8	ESR1,HSPA8	629
ESR1	LPL	ESR1,HSPA8,LPL	629
ESR1	MPL	ESR1,MPL	629
ESR1	MVP	ESR1,MVP,TP53	629
ESR1	NCAM1	CNTN2,ESR1,NCAM1	629
ESR1	NPPA	ESR1,HNF4A,NPPA	629
ESR1	NR3C1	ESR1,HNF4A,NR3C1,PGR,RORA	629
ESR1	PACSIN3	ESR1,HSPA8,PACSIN3	629
ESR1	PGR	ESR1,HNF4A,NR3C1,PGR,RORA	629
ESR1	PIK3CA	ESR1,PIK3CA	629
ESR1	RAG2	ESR1,RAG2,TP53	629
ESR1	RORA	ESR1,HIF1A,HNF4A,NR3C1,PGR,RORA	629
ESR1	RTKN	ESR1,RTKN	629
ESR1	RXRA	ESR1,RXRA,XIAP	629
ESR1	SDC1	ESR1,SDC1	629
ESR1	SET	ESR1,SET	629
ESR1	STAT5A	ESR1,STAT5A	629
ESR1	TF	ESR1,TF	629
ESR1	TP53	ESR1,TP53	629
ESR1	XIAP	ESR1,RXRA,XIAP	629
ESR1	ZNF79	ESR1,ZNF79	629
FLNA	ABL1	ABL1,BRCA1,DOK2,FLNA,HSP90AA1,PIK3R1	629
FLNA	AZGP1	AZGP1,FLNA	629
FLNA	BIRC3	BIRC3,FLNA	629
FLNA	BRCA1	BRCA1,FLNA,HSP90AA1,PIK3R1	629
FLNA	CAV1	CAV1,FLNA	629
FLNA	CD36	CD36,FLNA	629
FLNA	CD44	CD44,FLNA,ITGA4	629
FLNA	CEACAM1	CEACAM1,FLNA,SRC	629

FLNA	CHD2	CHD2,FLNA	629
FLNA	CLTC	CLTC,FLNA	629
FLNA	DOK2	DOK2,FLNA,SRC	629
FLNA	ETNK1	ETNK1,FLNA	629
FLNA	GGH	FLNA,GGH	629
FLNA	GNAI1	FLNA,GNAI1	629
FLNA	HSP90AA1	FLNA,HSP90AA1	629
FLNA	HSPA8	FLNA,HSPA8	629
FLNA	ILK	FLNA,ILK	629
FLNA	ITGA4	FLNA,ITGA4	629
FLNA	KRAS	FLNA,KRAS	629
FLNA	MAPK14	FLNA,MAPK14	629
FLNA	MYH11	FLNA,MYH11	629
FLNA	PFDN4	FLNA,PFDN4	629
FLNA	PIK3R1	FLNA,PIK3R1	629
FLNA	PKIA	FLNA,HSP90AA1,PKIA	629
FLNA	PPARG	FLNA,PPARG	629
FLNA	PTEN	FLNA,HSP90AA1,PIK3R1,PTEN	629
FLNA	PTK2B	FLNA,HSP90AA1,PTK2B	629
FLNA	RASA1	FLNA,RASA1	629
FLNA	SELL	FLNA,SELL	629
FLNA	SRC	FLNA,PIK3R1,SRC	629
FLNA	TP53	FLNA,TP53	629
FLNA	WTAP	FLNA,WTAP	629
FYN	ANPEP	ANPEP,CD9,FYN	629
FYN	BTK	BTK,CD19,CD22,CD79A,CD9,CR2,FYN,SYK	629
FYN	CARD11	CARD11,FYN,PRKCQ,ZAP70	629
FYN	CD19	CD19,CD22,CD79A,CD9,CR2,FYN,SYK	629
FYN	CD2	CD2,CD5,CD58,FYN	629
FYN	CD22	CD19,CD22,CD79A,CD9,CR2,FYN,SYK	629
FYN	CD5	CD2,CD5,CD58,CD72,CD79A,FYN,ZAP70	629
FYN	CD58	CD2,CD5,CD58,FYN	629
FYN	CD72	CD2,CD5,CD72,CD79A,FYN,SYK,ZAP70	629
FYN	CD79A	CD19,CD22,CD79A,CD9,CR2,FYN	629
FYN	CD86	CD86,CTLA4,FYN	629
FYN	CD9	BTK,CD19,CD22,CD79A,CD9,CR2,FYN,SYK	629
FYN	CR2	CD19,CD22,CD9,CR2,FYN,SYK	629
FYN	CSF1R	CSF1R,FYN,SRC	629
FYN	CSF2RB	CSF2RB,FYN,SRC,SYK	629
FYN	CTLA4	CD86,CTLA4,FYN	629
FYN	DLX4	DLX4,FYN,SRC	629
FYN	DOK1	DOK1,FYN,SRC	629
FYN	FLT1	FLT1,FYN	629

FYN	GUCY2D	FYN,GUCY2D	629
FYN	IL2RB	FYN,IL2RB,IL7R	629
FYN	IL6ST	FYN,IL6ST,LIFR	629
FYN	IL7R	FYN,IL2RB,IL7R,MS4A1	629
FYN	LIFR	FYN,IL6ST,LIFR	629
FYN	MAP4K1	FYN,MAP4K1	629
FYN	MS4A1	FYN,IL7R,MS4A1	629
FYN	PKIA	FYN,PKIA	629
FYN	PRKCQ	BTK,FYN,PRKCQ	629
FYN	RAPGEF1	FYN,RAPGEF1,SRC	629
FYN	RHOH	CARD11,FYN,RHOH,ZAP70	629
FYN	SPN	FYN,SPN	629
FYN	SRC	FYN,SRC	629
FYN	SYK	BTK,CD19,CD22,CD79A,CD9,CR2,FYN,SYK	629
FYN	THY1	FYN,THY1	629
FYN	TNFRSF10B	FYN,TNFRSF10B	629
FYN	ZAP70	FYN,ZAP70	629
GRB2	ANGPT1	ANGPT1,GRB2	629
GRB2	ASXL1	ASXL1,GRB2,STAT3	629
GRB2	BAD	BAD,GRB2	629
GRB2	BCR	BCR,CBL,GRB2,JAK2	629
GRB2	BLNK	BLNK,BTK,CBL,CD72,GRB2,SYK	629
GRB2	BTK	BTK,CBL,CD19,CD22,CD9,GRB2,SYK	629
GRB2	CASP2	CASP2,GRB2	629
GRB2	CBL	CBL,CD19,GRB2,JAK2,STAT3	629
GRB2	CCR5	CCR5,CD4,GRB2,LCK,STAT3	629
GRB2	CD19	CBL,CD19,CD22,CD9,GRB2,SYK	629
GRB2	CD1A	CD1A,GRB2	629
GRB2	CD22	CBL,CD19,CD22,CD9,GRB2,PTPRC,SYK	629
GRB2	CD36	CD36,CD9,GRB2	629
GRB2	CD4	CD4,CD86,GRB2,LCK,PIK3CA,PTPRC	629
GRB2	CD72	BLNK,CD4,CD72,GRB2,LCK,SYK,ZAP70	629
GRB2	CD86	CD4,CD86,GRB2,LCK,PIK3CA,PTPRC	629
GRB2	CD9	BTK,CBL,CD19,CD22,CD9,GRB2,SYK	629
GRB2	CDKN1B	CDKN1B,GRB2,JAK2	629
GRB2	CSF1R	CBL,CSF1R,GRB2,KIT,SOCS1,SRC	629
GRB2	CSF2RB	CSF2RB,EPO,EPOR,GRB2,JAK2,KIT,KITLG,PTPRC,SRC,SYK	629
GRB2	CSF3	CSF3,GRB2,JAK1,JAK2,STAT3,SYK	629
GRB2	DLX4	DLX4,GRB2,SRC	629
GRB2	DOK1	DOK1,GRB2,INPP5D,SRC	629
GRB2	E2F1	E2F1,GRB2	629
GRB2	EGF	EGF,FGFR2,GAPDH,GRB2	629

GRB2	EPO	CBL,CSF2RB,EPO,EPOR,GRB2,JAK2,KIT,KITLG,PTPRC,SRC,SYK	629
GRB2	EPOR	EPO,EPOR,GRB2,PTPRC,SRC	629
GRB2	ETV6	ETV6,GRB2,SOCS1	629
GRB2	FASLG	FASLG,GRB2,SRC	629
GRB2	FGFR2	FGFR2,GRB2,STAT3	629
GRB2	FLT1	CBL,FLT1,GRB2,STAT3	629
GRB2	FLT3	CASP2,CBL,FLT3,GRB2,KITLG	629
GRB2	GAPDH	GAPDH,GRB2	629
GRB2	GC	CBL,GC,GRB2,TF	629
GRB2	GFAP	GFAP,GRB2	629
GRB2	ICOS	GAPDH,GRB2,ICOS,KIT,SOCS1	629
GRB2	IL15	GRB2,IL15,IL2RB,IL2RG	629
GRB2	IL2RB	GRB2,IL15,IL2RB,IL2RG,STAT3	629
GRB2	IL2RG	GRB2,IL2RB,IL2RG,JAK2	629
GRB2	IL5RA	GRB2,IL5RA,JAK2,PTPN11,RAPGEF1	629
GRB2	IL6ST	GRB2,IL6ST,JAK2,LIFR,STAT3	629
GRB2	ILK	GRB2,ILK,LCK,ZAP70	629
GRB2	INPP5D	CBL,GRB2,INPP5D,JAK1	629
GRB2	JAK1	GRB2,INPP5D,JAK1,JAK2,STAT3	629
GRB2	JAK2	CDKN1B,GRB2,JAK2,STAT3	629
GRB2	KIT	CSF2RB,EPO,EPOR,GRB2,INPP5D,KIT,KITLG,PTPRC,SRC,SYK,ZAP70	629
GRB2	KITLG	BCR,CSF2RB,EPOR,FLT3,GRB2,KIT,KITLG,PTPN11,PTPRC,SOCS1	629
GRB2	LCK	CD4,CD86,FASLG,GRB2,LCK,PIK3CA,PTPRC,ZAP70	629
GRB2	LIFR	GRB2,IL6ST,LIFR	629
GRB2	MAP4K1	GRB2,MAP4K1	629
GRB2	MAPK14	GRB2,MAPK14,ZAP70	629
GRB2	MUC1	GRB2,MUC1,SRC	629
GRB2	MUSK	GRB2,MUSK	629
GRB2	MYH11	GRB2,MYH11	629
GRB2	NISCH	GRB2,NISCH	629
GRB2	NOS2	GRB2,NOS2	629
GRB2	PIK3CA	GRB2,PIK3CA	629
GRB2	PPARG	GRB2,PPARG	629
GRB2	PTPN11	DOK1,E2F1,GRB2,ILK,PTPN11,SOCS1,STAT3	629
GRB2	PTPRC	CD4,CD86,GRB2,LCK,PIK3CA,PTPRC	629
GRB2	RAPGEF1	CBL,GRB2,RAPGEF1,SRC	629
GRB2	SLC8A1	GRB2,SLC8A1	629
GRB2	SOCS1	GRB2,SOCS1	629
GRB2	SRC	GRB2,INPP5D,SRC	629

GRB2	STAT3	GRB2,JAK2,STAT3	629
GRB2	SYK	BTK,CBL,CD19,CD22,CD9,GRB2,SYK	629
GRB2	SYNJ1	GRB2,SYNJ1	629
GRB2	TF	GRB2,TF	629
GRB2	ZAP70	GRB2,ZAP70	629
HDAC1	AFF1	AFF1,HDAC1	629
HDAC1	ANKRD49	ANKRD49,EZH2,HDAC1	629
HDAC1	APEX1	APEX1,HDAC1	629
HDAC1	BCL11B	BCL11B,HDAC1	629
HDAC1	BCL3	BCL3,HDAC1,TBL1XR1	629
HDAC1	CBFB	CBFB,HDAC1	629
HDAC1	CDK1	CDK1,EZH2,HDAC1	629
HDAC1	CHFR	CHFR,HDAC1	629
HDAC1	CTCF	CTCF,HDAC1	629
HDAC1	DNMT1	DNMT1,HDAC1	629
HDAC1	DNTT	DNTT,HDAC1	629
HDAC1	EZH2	CDK1,EZH2,HDAC1,SF3B1	629
HDAC1	FBXW7	FBXW7,HDAC1,ZMYM2	629
HDAC1	FOXP3	FOXP3,HDAC1	629
HDAC1	HDAC9	HDAC1,HDAC9	629
HDAC1	HOXA9	HDAC1,HOXA9,MEIS1	629
HDAC1	KLF4	HDAC1,KLF4	629
HDAC1	MECOM	HDAC1,MECOM	629
HDAC1	MEIS1	HDAC1,HOXA9,MEIS1	629
HDAC1	PFDN4	HDAC1,PFDN4	629
HDAC1	PHF6	HDAC1,PHF6	629
HDAC1	PPARG	HDAC1,PPARG	629
HDAC1	RUNX1T1	HDAC1,RUNX1T1	629
HDAC1	SALL4	HDAC1,SALL4	629
HDAC1	SAMSN1	HDAC1,SAMSN1	629
HDAC1	SETD2	DNMT1,FBXW7,HDAC1,SETD2	629
HDAC1	SF3B1	HDAC1,SF3B1	629
HDAC1	SLC3A1	HDAC1,SLC3A1	629
HDAC1	SRSF2	HDAC1,SRSF2	629
HDAC1	TAL1	HDAC1,TAL1	629
HDAC1	TBL1XR1	HDAC1,TBL1XR1	629
HDAC1	UIMC1	HDAC1,UIMC1	629
HDAC1	ZMYM2	CTCF,HDAC1,ZMYM2	629
HDAC1	ZNF79	HDAC1,MECOM,ZNF79	629
LYN	ANGPT1	ANGPT1,ANGPT2,LYN	629
LYN	ANGPT2	ANGPT1,ANGPT2,LYN	629
LYN	BLNK	BLNK,BTK,CD72,CRKL,LYN,SYK	629
LYN	BTK	BTK,CD19,CD22,CD9,LYN,SYK	629

LYN	CASP9	CASP9,LYN	629
LYN	CD19	CD19,CD22,CD9,LYN,SYK	629
LYN	CD22	CD19,CD22,CD9,LYN,SYK	629
LYN	CD36	CD36,CD9,LYN	629
LYN	CD72	BLNK,CD72,LYN,SYK	629
LYN	CD9	BTK,CD19,CD22,CD9,LYN,SYK	629
LYN	CRKL	CRKL,DOK1,LYN,SRC	629
LYN	CSF2RB	CSF2RB,KITLG,LYN,SRC,SYK	629
LYN	CSF3	CSF3,LYN,SYK	629
LYN	CTLA4	CTLA4,LYN	629
LYN	DOK1	CRKL,DOK1,INPP5D,LYN,SRC	629
LYN	FGR	FGR,INPP5D,LYN,SRC	629
LYN	GP1BA	GP1BA,ITGAM,LYN,SRC,SYK	629
LYN	IL1B	IL1B,LYN,PDGFRB,SRC	629
LYN	IL5RA	IL5RA,LYN	629
LYN	IL6	IL6,LYN	629
LYN	IL7R	IL7R,LYN,MS4A1	629
LYN	INPP5D	CRKL,INPP5D,LYN	629
LYN	ITGAM	GP1BA,ITGAM,LYN,SRC,SYK	629
LYN	ITGAX	ITGAX,LYN,SYK	629
LYN	KITLG	CSF2RB,KITLG,LYN	629
LYN	LIF	IL6,LIF,LYN	629
LYN	LPL	LPL,LYN	629
LYN	MS4A1	IL7R,LYN,MS4A1	629
LYN	PDGFRB	IL1B,LYN,PDGFRB,SRC	629
LYN	POU2F2	LYN,POU2F2	629
LYN	PRKCQ	BTK,LYN,PRKCQ	629
LYN	SRC	INPP5D,LYN,SRC	629
LYN	SYK	BTK,CD19,CD22,CD9,LYN,SYK	629
SHC1	AMD1	AMD1,SHC1	629
SHC1	BCR	BCR,JAK2,RB1,SHC1	629
SHC1	CLPB	CLPB,SHC1	629
SHC1	CSF1R	CSF1R,FGFR1,SHC1	629
SHC1	DOK1	DOK1,DOK2,SHC1	629
SHC1	DOK2	DOK2,SHC1	629
SHC1	FCGR3A	FCGR3A,SHC1	629
SHC1	FGFR1	FGFR1,SHC1	629
SHC1	FGFR2	FGFR1,FGFR2,SHC1	629
SHC1	FLT1	FLT1,SHC1	629
SHC1	FLT3	FLT3,SHC1	629
SHC1	GHR	GHR,SHC1	629
SHC1	IL15	IL15,IL2RB,IL2RG,IL4,SHC1	629
SHC1	IL2RB	IL15,IL2RB,IL2RG,IL4,IL4R,SHC1	629

SHC1	IL2RG	IL2RB,IL2RG,IL4,IL4R,JAK2,SHC1	629
SHC1	IL4	IL2RB,IL2RG,IL4,IL4R,SHC1	629
SHC1	IL4R	IL2RG,IL4,IL4R,JAK2,SHC1	629
SHC1	JAK2	JAK2,SHC1	629
SHC1	LRP1	LRP1,SHC1	629
SHC1	MAPKAPK2	MAPKAPK2,SHC1	629
SHC1	MAT1A	MAT1A,MAT2A,SHC1	629
SHC1	MAT2A	MAT1A,MAT2A,SHC1	629
SHC1	MME	MME,SHC1	629
SHC1	MPL	JAK2,MPL,SHC1	629
SHC1	MYH11	MYH11,SHC1	629
SHC1	NF1	NF1,SHC1	629
SHC1	PTPN11	DOK1,PTPN11,SHC1	629
SHC1	RAPGEF1	RAPGEF1,SHC1	629
SHC1	RB1	RB1,SHC1	629
SHC1	TET1	SHC1,TET1	629
STAT1	AKT1	AKT1,STAT1	629
STAT1	ATM	ATM,STAT1	629
STAT1	CASP3	AKT1,CASP3,STAT1	629
STAT1	CCR1	CCR1,STAT1	629
STAT1	CCR5	CCR5,CXCR4,LCK,STAT1	629
STAT1	CD40	CD40,STAT1	629
STAT1	CXCR4	CXCR4,STAT1	629
STAT1	FLT1	FLT1,KDR,STAT1	629
STAT1	FOS	FOS,STAT1	629
STAT1	FOXP3	FOXP3,STAT1	629
STAT1	GFAP	AKT1,GFAP,STAT1	629
STAT1	HPS4	HPS4,PIK3CA,STAT1	629
STAT1	HSPA4L	HSPA4L,STAT1	629
STAT1	IFNAR2	IFNAR2,STAT1	629
STAT1	IFNG	FOS,IFNG,JAK2,PTPN11,STAT1	629
STAT1	IL1B	AKT1,IL1B,IRAK1,PDGFRB,STAT1	629
STAT1	IL2RA	IL2RA,STAT1,TBX21	629
STAT1	IL2RG	IL2RG,JAK2,STAT1	629
STAT1	IRAK1	AKT1,IL1B,IRAK1,STAT1	629
STAT1	IRF8	IL1B,IRF8,PTPN11,STAT1	629
STAT1	JAK1	JAK1,JAK2,STAT1	629
STAT1	JAK2	JAK2,NFKBIA,STAT1	629
STAT1	KDR	KDR,STAT1	629
STAT1	KIT	KIT,STAT1	629
STAT1	LCK	LCK,PIK3CA,STAT1	629
STAT1	MYC	MYC,STAT1	629
STAT1	NFKBIA	AKT1,NFKBIA,STAT1,XPO1	629

STAT1	PDGFRB	AKT1,IL1B,IRAK1,PDGFRB,STAT1	629
STAT1	PIK3CA	AKT1,PIK3CA,STAT1	629
STAT1	PSMC3	PSMC3,STAT1	629
STAT1	PTPN11	AKT1,PSMC3,PTPN11,STAT1	629
STAT1	STAT5A	STAT1,STAT5A	629
STAT1	TBX21	IL2RA,STAT1,TBX21	629
STAT1	TP73	STAT1,TP73,XPO1	629
STAT1	XPO1	STAT1,XPO1	629

Sample 773

**Candidate AML genes**	**Known AML genes** (of which the module includes candidate AML genes)	**ALL the AML genes involved in current module**	**Sample-ID**
ESR1	AKT1	AKT1,ESR1	773
ESR1	ANKRD49	ANKRD49,ESR1,EZH2	773
ESR1	BCR	BCR,ESR1,PTPN11	773
ESR1	BIRC5	BIRC5,ESR1,XIAP	773
ESR1	BLNK	BLNK,ESR1,KIT,PDGFRB	773
ESR1	CAD	CAD,ESR1,XPO1	773
ESR1	CASP3	CASP3,CTNNB1,ESR1	773
ESR1	CBL	CBL,ESR1	773
ESR1	CCR5	CCR5,ESR1	773
ESR1	CHD2	CHD2,ESR1	773
ESR1	CNTN2	CNTN2,ESR1,NCAM1	773
ESR1	COX10	COX10,ESR1	773
ESR1	CTNNB1	CTNNB1,ESR1	773
ESR1	DIABLO	DIABLO,ESR1	773
ESR1	DLX4	DLX4,ESR1,NFKB1	773
ESR1	EDNRA	EDNRA,ESR1	773
ESR1	ETV6	ESR1,ETV6	773
ESR1	EZH2	ESR1,EZH2,RARA	773
ESR1	GGH	ESR1,GGH	773
ESR1	HBA2	ESR1,HBA2,HBB	773
ESR1	HBB	ESR1,HBA2,HBB	773
ESR1	KIT	ESR1,KIT,PDGFRB	773
ESR1	MAPKAPK2	CTNNB1,ESR1,MAPKAPK2	773
ESR1	MVP	ESR1,MVP	773
ESR1	NCAM1	CNTN2,ESR1,NCAM1	773
ESR1	NFKB1	ESR1,NFKB1	773
ESR1	NME2	ESR1,NME2,VIM	773
ESR1	NPM1	ESR1,NPM1	773
ESR1	NPPA	ESR1,NPPA	773

ESR1	PARP4	ESR1,MVP,PARP4	773
ESR1	PDGFRB	ESR1,KIT,PDGFRB	773
ESR1	PPP2R3A	ESR1,PPP2R3A	773
ESR1	PTPN11	ESR1,PTPN11	773
ESR1	RAG2	ESR1,RAG2	773
ESR1	RARA	ESR1,RARA	773
ESR1	RBBP8	ESR1,RBBP8	773
ESR1	ROS1	ESR1,ROS1	773
ESR1	SDC1	ESR1,NCAM1,SDC1	773
ESR1	TCF15	ESR1,EZH2,TCF15	773
ESR1	VIM	AKT1,CTNNB1,ESR1,VIM	773
ESR1	XIAP	CTNNB1,ESR1,XIAP	773
ESR1	XPO1	ESR1,XPO1	773
FAF1	AKT1	AKT1,FAF1	773
FAF1	CASP8	CASP8,CDKN1A,FAF1,GAPDH,HSPA5	773
FAF1	CD40	CD40,FAF1,HSPA4,HSPA8	773
FAF1	CDKN1A	AKT1,CDKN1A,FAF1,GAPDH,SET	773
FAF1	CREBBP	CREBBP,FAF1,RUNX1	773
FAF1	CTNNB1	CASP8,CTNNB1,FAF1	773
FAF1	FAS	FAF1,FAS	773
FAF1	GAPDH	AKT1,CDKN1A,CREBBP,CTNNB1,FAF1,FAS,GAPDH,SET	773
FAF1	GFAP	FAF1,GFAP	773
FAF1	HSP90AA1	FAF1,HSP90AA1,RPL5	773
FAF1	HSPA4	AKT1,CDKN1A,CREBBP,FAF1,FAS,HSP90AA1,HSPA4,SET	773
FAF1	HSPA4L	FAF1,HSPA4L,HSPA5,HSPA8	773
FAF1	HSPA5	AKT1,CASP8,CDKN1A,CREBBP,FAF1,GAPDH,HSPA5,SET	773
FAF1	HSPA8	AKT1,CDKN1A,FAF1,FAS,GAPDH,HSP90AA1,HSPA8,SET	773
FAF1	HSPD1	AKT1,CDKN1A,CTNNB1,FAF1,FAS,GAPDH,HSPD1,NR4A1,SET	773
FAF1	LMOD1	FAF1,HSP90AA1,LMOD1,NPM1	773
FAF1	MRC2	FAF1,HSPD1,MRC2	773
FAF1	NPM1	CDKN1A,FAF1,NPM1,RPL5	773
FAF1	NR3C2	FAF1,HSP90AA1,NR3C2	773
FAF1	NR4A1	AKT1,FAF1,NR4A1	773
FAF1	PARP1	AKT1,CASP8,CDKN1A,FAF1,FAS,GAPDH,HSP90AA1,PARP1,SET	773
FAF1	PHB2	AKT1,CDKN1A,CREBBP,FAF1,FAS,HSP90AA1,PHB2	773
FAF1	POU2F2	FAF1,GAPDH,POU2F2	773

FAF1	PTHLH	CDKN1A,FAF1,PTHLH	773
FAF1	RPL19	FAF1,RPL19,RPL5	773
FAF1	RPL5	AKT1,CASP8,CDKN1A,CTNNB1,FAF1,FAS,GAPDH,HSP90AA1,RPL5,SET	773
FAF1	RUNX1	FAF1,RUNX1	773
FAF1	SET	FAF1,GAPDH,SET	773
FAF1	SETBP1	CDKN1A,CTNNB1,FAF1,SET,SETBP1	773
FAF1	SKP1	FAF1,HSPD1,SKP1	773
FAF1	SLC22A2	FAF1,SLC22A2	773
FAF1	ST13	FAF1,ST13	773
FAF1	TNFSF10	CASP8,FAF1,TNFSF10	773
FAF1	XIAP	CASP8,CTNNB1,FAF1,XIAP	773
FYN	ANPEP	ANPEP,FYN	773
FYN	BAD	BAD,FYN,MAPK3,SRC	773
FYN	BAX	BAX,FYN,MAPK3,SRC	773
FYN	BCR	BCR,FYN,MAPK1,MAPK3,SRC	773
FYN	BRAF	BRAF,FYN,MAPK3,SRC	773
FYN	BTK	BTK,FYN,PML,RAF1	773
FYN	C5	C5,FYN	773
FYN	CD14	CD14,FYN,PICALM	773
FYN	CD4	CD4,CD79A,EPOR,FCGR3A,FYN,IFNAR1,RASA1,SRC	773
FYN	CD5	CD4,CD5,CD79A,FYN	773
FYN	CD72	CD5,CD72,FYN	773
FYN	CD79A	CD4,CD79A,FCGR3A,FYN,IFNAR1,RASA1,SRC	773
FYN	CR2	CD79A,CR2,FYN	773
FYN	CSF2RB	BCR,CSF2RB,EPOR,FYN	773
FYN	DNM2	DNM2,FYN,MKI67	773
FYN	DOK1	DOK1,FYN,SRC	773
FYN	EPOR	CD4,CD79A,EPOR,FCGR3A,FYN,RASA1,SRC	773
FYN	FCGR3A	CD79A,EPOR,FCGR3A,FYN,RASA1,SRC	773
FYN	FGFR2	FGFR2,FYN	773
FYN	FLT1	FLT1,FYN	773
FYN	GHR	FYN,GHR,SRC	773
FYN	GUCY2D	FYN,GUCY2D	773
FYN	HBEGF	CD4,FYN,HBEGF	773
FYN	IFNAR1	CD4,CD79A,FCGR3A,FYN,IFNAR1,RASA1,SRC	773
FYN	IL2RB	FYN,IL2RB	773
FYN	IL7	FYN,IL7,MS4A1	773
FYN	JAK3	FYN,JAK3,PML,RAF1	773
FYN	MAP4K1	BRAF,FYN,MAP4K1,RAF1	773

FYN	MAPK1	BAX,FYN,MAPK1,MAPK3,SRC	773
FYN	MAPK3	BAX,FYN,MAPK3,SRC	773
FYN	MAT1A	FYN,MAT1A	773
FYN	MKI67	DNM2,FYN,MKI67	773
FYN	MS4A1	FYN,MS4A1	773
FYN	PICALM	DNM2,FYN,PICALM	773
FYN	PKIA	BAD,FYN,PKIA	773
FYN	PML	FYN,PML,RAF1	773
FYN	POU2F2	FYN,POU2F2	773
FYN	PRDX2	BAX,FYN,PRDX2	773
FYN	RAF1	BAX,FYN,MAPK3,RAF1,SRC	773
FYN	RAPGEF1	FYN,RAPGEF1,SRC	773
FYN	RASA1	FYN,RASA1	773
FYN	RHOH	CD4,FYN,RASA1,RHOH	773
FYN	SNCA	BAX,FYN,MAPK3,SNCA,SRC	773
FYN	SOCS1	FYN,SOCS1	773
FYN	SRC	BAX,FYN,MAPK3,SRC	773
FYN	TFRC	FYN,TFRC	773
FYN	VANGL1	FYN,VANGL1	773
GRB2	ABCB1	ABCB1,GRB2	773
GRB2	BCR	BCR,GRB2	773
GRB2	BLNK	BLNK,GRB2,KIT,PDGFRB	773
GRB2	BTK	BTK,GRB2,JAK1,PML	773
GRB2	CA2	CA2,GRB2	773
GRB2	CBL	CBL,GRB2	773
GRB2	CCR5	CCR5,DNM2,GRB2,JAK2	773
GRB2	CD2	CD2,FGFR2,GRB2,MKI67,PTPRC	773
GRB2	CD72	BLNK,CD72,GRB2	773
GRB2	CD86	CD86,GRB2,PTPRC	773
GRB2	CDKN1B	CDKN1B,GRB2,JAK2,XPO1	773
GRB2	CR2	CBL,CR2,GRB2	773
GRB2	CSF3	CSF3,GRB2,JAK1,JAK2	773
GRB2	DLX4	DLX4,GRB2	773
GRB2	DNM2	DNM2,GRB2,MKI67	773
GRB2	DOK1	DOK1,DOK2,GRB2	773
GRB2	DOK2	DOK2,GRB2	773
GRB2	EFHC1	EFHC1,GRB2	773
GRB2	EPO	EPO,GRB2,JAK2,KIT,KITLG,PTPRC	773
GRB2	FASLG	FASLG,GRB2	773
GRB2	FGFR2	CBL,FGFR2,GRB2,PTK2B	773
GRB2	FLT1	CBL,FLT1,GRB2	773
GRB2	FLT3	CBL,FLT3,GRB2	773
GRB2	GC	GC,GRB2,MKI67	773

GRB2	IL15	GRB2,IL15,IL2RB,IL2RG,IL4R,IL9R,JAK2	773
GRB2	IL2RB	GRB2,IL2RB,IL2RG,IL4R,IL9R,JAK1,JAK2	773
GRB2	IL2RG	GRB2,IL2RB,IL2RG,IL4R,IL9R,JAK1	773
GRB2	IL4R	GRB2,IL15,IL2RB,IL2RG,IL4R,IL9R,JAK2	773
GRB2	IL9R	GRB2,IL15,IL2RB,IL2RG,IL4R,IL9R,JAK2	773
GRB2	ILK	GRB2,ILK	773
GRB2	ITGA4	GRB2,ITGA4,MYH11	773
GRB2	JAK1	GRB2,JAK1,PML	773
GRB2	JAK2	CBL,CDKN1B,GRB2,JAK1,JAK2,PTPRC	773
GRB2	JAK3	GRB2,JAK1,JAK3,PML	773
GRB2	KIT	GRB2,KIT,PDGFRB	773
GRB2	KITLG	EPO,FLT3,GRB2,KIT,KITLG	773
GRB2	MAP4K1	GRB2,MAP4K1	773
GRB2	MKI67	DNM2,GRB2,MKI67	773
GRB2	MUSK	GRB2,MUSK	773
GRB2	MYH11	GRB2,ITGA4,MYH11	773
GRB2	PDGFRB	GRB2,KIT,PDGFRB,PTPRC	773
GRB2	PICALM	DNM2,GRB2,PICALM	773
GRB2	PML	GRB2,JAK1,PML	773
GRB2	PTK2B	GRB2,PTK2B	773
GRB2	PTPRC	CBL,GRB2,PTPRC	773
GRB2	RAPGEF1	GRB2,RAPGEF1	773
GRB2	SLC22A2	GRB2,SLC22A2	773
GRB2	SLC8A1	GRB2,SLC8A1	773
GRB2	SYNJ1	GRB2,SYNJ1	773
GRB2	TFRC	GRB2,ITGA4,TFRC	773
GRB2	WNK2	GRB2,WNK2	773
GRB2	XPO1	GRB2,XPO1	773
HDAC1	AFF1	AFF1,HDAC1	773
HDAC1	BCL11B	BCL11B,HDAC1	773
HDAC1	BCL3	BCL3,HDAC1	773
HDAC1	CEBPB	CEBPB,GATA3,HDAC1,IRF4,SPI1	773
HDAC1	CHFR	CHFR,HDAC1	773
HDAC1	CTCF	CTCF,HDAC1	773
HDAC1	DNMT1	DNMT1,DNMT3A,HDAC1,SPI1	773
HDAC1	DNMT3A	CEBPB,DNMT3A,GATA3,HDAC1,IRF4,SPI1	773
HDAC1	E2F1	CEBPB,E2F1,HDAC1,TP73	773
HDAC1	FOXP3	FOXP3,HDAC1	773
HDAC1	GATA2	GATA2,HDAC1,TAL1	773
HDAC1	GATA3	CEBPB,DNMT3A,GATA3,HDAC1,IRF4,SPI1	773
HDAC1	GGH	GGH,HDAC1	773
HDAC1	HDAC9	CEBPB,HDAC1,HDAC9,ZBTB16	773
HDAC1	HOXA9	HDAC1,HOXA9	773

HDAC1	IRF4	CEBPB,DNMT3A,GATA3,HDAC1,IRF4,SPI1	773
HDAC1	IRF8	CEBPB,GATA3,HDAC1,IRF4,IRF8,SPI1	773
HDAC1	KLF4	HDAC1,KLF4	773
HDAC1	MMP9	HDAC1,MMP9	773
HDAC1	PIK3CA	HDAC1,PIK3CA	773
HDAC1	RUNX1T1	HDAC1,RUNX1T1	773
HDAC1	SAMSN1	HDAC1,SAMSN1	773
HDAC1	SPI1	CEBPB,DNMT3A,HDAC1,SPI1	773
HDAC1	STAT5A	CEBPB,HDAC1,STAT5A	773
HDAC1	TAL1	HDAC1,TAL1	773
HDAC1	TBL1XR1	HDAC1,TBL1XR1	773
HDAC1	TOP2A	CTCF,HDAC1,TOP2A,TOP2B	773
HDAC1	TOP2B	HDAC1,TOP2B	773
HDAC1	TP73	CEBPB,E2F1,HDAC1,TP73	773
HDAC1	WTAP	HDAC1,WTAP	773
HDAC1	XBP1	HDAC1,XBP1	773
HDAC1	ZBTB16	HDAC1,HDAC9,ZBTB16	773
HDAC1	ZMYM2	CTCF,HDAC1,ZMYM2	773
LYN	ANGPT2	ANGPT2,LYN	773
LYN	CASP2	CASP2,LYN	773
LYN	CASP9	CASP9,LYN	773
LYN	CBL	CBL,CRKL,LYN	773
LYN	CD36	CD36,LYN	773
LYN	CD72	CD72,LYN	773
LYN	CDK1	CDK1,LYN	773
LYN	CRKL	CBL,CRKL,DOK1,INPP5D,LYN	773
LYN	CSF2RB	CSF2RB,JAK2,LYN	773
LYN	CSF3	CSF3,JAK2,LYN	773
LYN	CTLA4	CTLA4,LYN,XPO1	773
LYN	DOK1	CRKL,DOK1,DOK2,INPP5D,LYN	773
LYN	DOK2	CRKL,DOK2,INPP5D,LYN	773
LYN	FASLG	FASLG,LYN	773
LYN	FGFR2	CBL,FGFR2,LYN,PTK2B	773
LYN	FGR	FGR,LYN	773
LYN	FLT3	CBL,CRKL,FLT3,LYN	773
LYN	GP1BA	GP1BA,LYN	773
LYN	IL1B	IL1B,LYN	773
LYN	IL5RA	CSF2RB,IL5RA,LYN	773
LYN	IL6ST	IL6ST,JAK2,LYN	773
LYN	IL7	CBL,IL7,LYN	773
LYN	INPP5D	CRKL,INPP5D,LYN	773
LYN	ITGAX	ITGAX,ITGB2,LYN,PTK2B	773
LYN	ITGB2	ITGAX,ITGB2,LYN	773

LYN	JAK2	CBL,JAK2,LYN	773
LYN	MME	LYN,MME	773
LYN	POU2F2	LYN,POU2F2	773
LYN	PTK2B	LYN,PTK2B	773
LYN	XPO1	CRKL,LYN,XPO1	773
RELA	AFF1	AFF1,RELA	773
RELA	BCL3	BCL3,RELA	773
RELA	CASP8	CASP8,GAPDH,HSPA5,RELA	773
RELA	CCND2	CCND2,PIK3CA,RELA	773
RELA	CDX2	CDX2,RELA	773
RELA	CHFR	CHFR,PARP1,RELA	773
RELA	CXCR3	CXCR3,RELA	773
RELA	CXCR4	CXCR4,HSPA8,RELA	773
RELA	CYP24A1	CYP24A1,RELA	773
RELA	EGF	EGF,NR4A1,RELA	773
RELA	ELF4	ELF4,HSPA5,RELA,VDR	773
RELA	FOXP3	FOXP3,RELA	773
RELA	GAPDH	GAPDH,RELA	773
RELA	HSPA5	CASP8,GAPDH,HSPA5,RELA	773
RELA	HSPA8	GAPDH,HSPA8,RELA	773
RELA	HSPD1	GAPDH,HSPD1,NR4A1,RELA	773
RELA	IL6	IL6,RELA	773
RELA	IRF8	IRF8,RELA	773
RELA	KDM6A	KDM6A,RELA,RXRA	773
RELA	KDR	KDR,RELA	773
RELA	KLF4	KDM6A,KLF4,RELA	773
RELA	LIFR	LIFR,RELA,RXRA	773
RELA	LYL1	LYL1,RELA	773
RELA	MAP4K1	MAP4K1,RELA	773
RELA	NFKBIB	NFKBIB,RELA	773
RELA	NR4A1	NR4A1,RELA	773
RELA	NSD1	AFF1,NSD1,RELA	773
RELA	PARP1	CASP8,GAPDH,PARP1,RELA	773
RELA	PBX1	PARP1,PBX1,RELA,RXRA	773
RELA	PGR	PARP1,PGR,RELA,STAT5A	773
RELA	PIK3CA	PIK3CA,RELA	773
RELA	PPARGC1A	PPARGC1A,RELA,STAT6,VDR	773
RELA	RPL5	CASP8,GAPDH,RELA,RPL5	773
RELA	RXRA	PARP1,PBX1,RELA,RXRA	773
RELA	STAT5A	RELA,STAT5A	773
RELA	STAT6	RELA,STAT6	773
RELA	TERT	CCND2,RELA,TERT	773
RELA	TF	HSPD1,RELA,TF	773

RELA	TP73	RELA,RPL5,TP73	773
RELA	VDR	RELA,STAT6,VDR	773
RELA	WTAP	RELA,WTAP	773
SHC1	AMD1	AMD1,SHC1	773
SHC1	BLNK	BLNK,CRKL,KIT,PDGFRB,SHC1	773
SHC1	CD2	CD2,FGFR2,SHC1	773
SHC1	CD38	CD38,SHC1	773
SHC1	CLPB	CLPB,CRKL,PTPN11,SHC1	773
SHC1	CRKL	CRKL,SHC1	773
SHC1	CSF1R	CSF1R,SHC1,TEK	773
SHC1	CSF2RB	CSF2RB,JAK2,KIT,SHC1	773
SHC1	FCGR3A	CD2,FCGR3A,PDGFRB,SHC1	773
SHC1	FGFR2	FGFR2,SHC1	773
SHC1	FLT1	CRKL,FLT1,SHC1	773
SHC1	FLT3	CRKL,FLT3,SHC1	773
SHC1	ICAM1	ICAM1,IL15,IL2RB,IL2RG,IL4,IL9R,SHC1	773
SHC1	IL15	ICAM1,IL15,IL2RB,IL2RG,IL4,IL4R,IL9R,JAK2,SHC1	773
SHC1	IL2	ICAM1,IL2,IL2RG,IL4,IL9R,SHC1	773
SHC1	IL2RB	ICAM1,IL2RB,IL2RG,IL4,IL4R,IL9R,JAK2,SHC1	773
SHC1	IL2RG	ICAM1,IL2RB,IL2RG,IL4,IL4R,IL9R,SHC1	773
SHC1	IL4	ICAM1,IL2RB,IL2RG,IL4,IL9R,SHC1	773
SHC1	IL4R	ICAM1,IL15,IL2RB,IL2RG,IL4,IL4R,IL9R,JAK2,SHC1	773
SHC1	IL5RA	CSF2RB,ICAM1,IL5RA,ILK,SHC1	773
SHC1	IL9R	ICAM1,IL15,IL2RB,IL2RG,IL4,IL4R,IL9R,JAK2,SHC1	773
SHC1	ILK	ILK,SHC1	773
SHC1	JAK2	JAK2,SHC1	773
SHC1	KIT	KIT,PDGFRB,SHC1	773
SHC1	KITLG	AMD1,CRKL,FLT3,KIT,KITLG,SHC1	773
SHC1	LIFR	LIFR,SHC1	773
SHC1	LRP1	LRP1,SHC1	773
SHC1	MAT1A	MAT1A,SHC1	773
SHC1	MPL	JAK2,MPL,SHC1	773
SHC1	PDGFRB	CRKL,KIT,PDGFRB,SHC1	773
SHC1	PRDX2	PRDX2,SHC1	773
SHC1	PTPN11	CRKL,PTPN11,SHC1	773
SHC1	RAPGEF1	CRKL,RAPGEF1,SHC1	773
SHC1	RB1	RB1,SHC1	773
SHC1	STAT5A	CRKL,PTPN11,SHC1,STAT5A	773
SHC1	TEK	PDGFRB,SHC1,TEK	773

STAT1	AKT1	AKT1,NFKBIA,STAT1	773
STAT1	ATM	ATM,STAT1	773
STAT1	BCL3	BCL3,FOS,STAT1	773
STAT1	BTG1	BTG1,STAT1	773
STAT1	BTK	BTK,JAK1,PML,STAT1,STAT3	773
STAT1	CASP3	CASP3,NFKBIA,STAT1	773
STAT1	CCR1	CCR1,STAT1,STAT3	773
STAT1	CCR5	CCR5,CXCR4,JAK2,LCK,STAT1,STAT3	773
STAT1	CD40	CD40,IL4R,STAT1	773
STAT1	CSF2RB	AKT1,CSF2RB,JAK2,STAT1,STAT3	773
STAT1	CXCR3	CXCR3,STAT1,STAT3	773
STAT1	CXCR4	CXCR4,STAT1,STAT3	773
STAT1	EGF	EGF,STAT1,STAT3	773
STAT1	FLT1	FLT1,STAT1,STAT3	773
STAT1	FOS	FOS,STAT1,STAT3	773
STAT1	FOXP3	FOXP3,STAT1,STAT3	773
STAT1	HSPA4L	HSPA4L,STAT1	773
STAT1	IFNA1	IFNA1,IFNA2,IFNAR2,ISG15,STAT1	773
STAT1	IFNA2	IFNA1,IFNA2,IFNAR2,ISG15,STAT1	773
STAT1	IFNAR2	IFNA1,IFNA2,IFNAR2,ISG15,STAT1	773
STAT1	IFNG	FOS,IFNG,JAK1,JAK2,PTPN11,STAT1	773
STAT1	IL15	IL15,IL2RB,IL2RG,IL4R,IL9R,JAK2,STAT1	773
STAT1	IL1B	AKT1,IL1B,PDGFRB,STAT1	773
STAT1	IL2RA	IL2RA,IL2RB,STAT1,STAT3,TBX21	773
STAT1	IL2RB	IL2RB,IL2RG,IL4R,IL9R,JAK1,JAK2,STAT1,STAT3	773
STAT1	IL2RG	IL2RB,IL2RG,IL4R,IL9R,JAK1,STAT1	773
STAT1	IL4R	IL15,IL2RB,IL2RG,IL4R,IL9R,JAK2,STAT1	773
STAT1	IL9R	IL15,IL2RB,IL2RG,IL4R,IL9R,JAK2,STAT1	773
STAT1	IRF8	FOS,IRF8,STAT1	773
STAT1	ISG15	IFNA1,IFNA2,ISG15,STAT1	773
STAT1	JAK1	JAK1,PML,STAT1,STAT3	773
STAT1	JAK2	JAK1,JAK2,NFKBIA,STAT1,STAT3	773
STAT1	JAK3	JAK1,JAK3,PML,STAT1,STAT3	773
STAT1	KITLG	KITLG,STAT1	773
STAT1	LCK	LCK,NFKBIA,STAT1,STAT3	773
STAT1	LIF	FOS,LIF,STAT1,STAT3	773
STAT1	NFKBIA	CASP3,NFKBIA,STAT1	773
STAT1	NOS2	NOS2,STAT1	773
STAT1	PDGFRB	PDGFRB,STAT1,STAT3	773
STAT1	PIK3CA	PIK3CA,PTPN11,STAT1	773
STAT1	PML	JAK1,PML,STAT1,STAT3	773
STAT1	PPARG	PPARG,PSMC3,STAT1	773

STAT1	PSMC3	PSMC3,STAT1	773
STAT1	PTPN11	JAK1,PTPN11,STAT1	773
STAT1	STAT3	JAK1,STAT1,STAT3	773
STAT1	STAT5A	PTPN11,STAT1,STAT3,STAT5A	773
STAT1	TBX21	IL2RA,IL2RB,STAT1,STAT3,TBX21	773
STAT1	VDR	NFKBIA,STAT1,VDR	773
TRAF6	AKT1	AKT1,TRAF6	773
TRAF6	BIRC2	BIRC2,CASP8,TRAF6	773
TRAF6	BIRC3	BIRC3,EED,JUN,RAD21,TRAF6	773
TRAF6	BLNK	BLNK,KIT,PDGFRB,TRAF6	773
TRAF6	CARD11	AKT1,BIRC2,CARD11,TNFRSF1A,TRAF6	773
TRAF6	CASP8	CASP8,TRAF6	773
TRAF6	CCR5	CCR5,JAK2,STAT3,TRAF6	773
TRAF6	CD40	BIRC2,CD40,TRAF6	773
TRAF6	CD44	CD44,STAT3,TRAF6	773
TRAF6	CD72	BLNK,CD72,STAT3,TRAF6	773
TRAF6	CNR2	CCR5,CNR2,TRAF6	773
TRAF6	CSF2RB	AKT1,CSF2RB,JAK2,KIT,STAT3,TRAF6	773
TRAF6	CYP24A1	CYP24A1,TRAF6	773
TRAF6	EED	EED,JUN,RAD21,TRAF6	773
TRAF6	EGR1	EGR1,STAT3,TRAF6	773
TRAF6	HMBS	HMBS,JUN,TRAF6	773
TRAF6	HSPB1	HSPB1,JUN,TNFRSF1A,TRAF6	773
TRAF6	IL18R1	IL18R1,STAT3,TRAF6	773
TRAF6	IL1B	AKT1,IL1B,IRAK1,PDGFRB,TRAF6	773
TRAF6	IL2	IL2,TRAF6	773
TRAF6	IL6ST	CD40,IL6ST,JAK2,STAT3,TRAF6	773
TRAF6	IRAK1	IRAK1,MYD88,TRAF6	773
TRAF6	IRF8	IRF8,JUN,TRAF6	773
TRAF6	JAK2	JAK2,STAT3,TRAF6	773
TRAF6	JUN	HSPB1,JUN,TNFRSF1A,TRAF6	773
TRAF6	KIT	KIT,PDGFRB,TRAF6	773
TRAF6	LTA	BIRC2,LTA,TNFRSF1A,TNFRSF1B,TRAF6	773
TRAF6	MAP2K1	EED,JUN,MAP2K1,RAD21,TRAF6	773
TRAF6	MAT1A	MAT1A,MAT2A,TRAF6	773
TRAF6	MAT2A	MAT2A,TRAF6	773
TRAF6	MBP	MBP,TRAF6	773
TRAF6	MVP	MVP,TRAF6	773
TRAF6	MYD88	IRAK1,MYD88,TRAF6	773
TRAF6	NF1	AKT1,NF1,TRAF6	773
TRAF6	PARP4	MVP,PARP4,TRAF6	773
TRAF6	PDGFRB	KIT,PDGFRB,STAT3,TRAF6	773
TRAF6	PIK3CA	PIK3CA,PTPN11,TRAF6	773

TRAF6	PRDX2	HSPB1,PRDX2,TRAF6	773
TRAF6	PTPN11	PTPN11,TRAF6	773
TRAF6	RAD21	JUN,RAD21,TRAF6	773
TRAF6	SET	JUN,SET,TRAF6	773
TRAF6	SIGLEC5	CASP8,SIGLEC5,TRAF6	773
TRAF6	ST13	IRAK1,JUN,ST13,TRAF6	773
TRAF6	STAT3	STAT3,TRAF6	773
TRAF6	TFRC	TFRC,TRAF6	773
TRAF6	TNFRSF1A	HSPB1,JUN,TNFRSF1A,TRAF6	773
TRAF6	TNFRSF1B	BIRC2,CD40,TNFRSF1A,TNFRSF1B,TRAF6	773
TRAF6	WBP1L	TNFRSF1A,TRAF6,WBP1L	773

Supplementary Table 6. Patient-specific candidate AML genes

Sample 018

Sample-specific Candidate AML genes	Number of specific interactions	In which module of known AML gene
PPA2	1	pos_gene,GPX4
WLS	3	pos_gene,C5,KMT2C,SLC35B2
UBR2	0	pos_gene:None
WNK1	1	pos_gene,WNK2
CSTF3	0	pos_gene:None
LRIG1	0	pos_gene:None
ADCY1	2	pos_gene,F2RL1,GNAS
NETO2	2	pos_gene,EGF,LSR
SLC39A8	2	pos_gene,PTCH1,SLC35B2
GET4	0	pos_gene:None
TAF7	0	pos_gene:None
ENC1	0	pos_gene:None
ZDHHC9	2	pos_gene,HBEGF,IFNA2
LIPH	0	pos_gene:None
NDUFA3	5	pos_gene,CD72,EGF,LSR,NRAS,SLC3A1
RANBP3	0	pos_gene:None
NR5A1	0	pos_gene:None
RASSF1	0	pos_gene:None
PPP1R15A	2	pos_gene,BAX,BRAF
BRD2	0	pos_gene:None
NOP2	0	pos_gene:None
BABAM1	0	pos_gene:None
MAS1	0	pos_gene:None
FBXL7	7	pos_gene,BIRC5,SKP1
EHD4	0	pos_gene:None
NCAPG2	0	pos_gene:None
SLC16A2	1	pos_gene,CD53
ATP1B3	0	pos_gene:None
SEC22A	1	pos_gene,CNR1
ABLIM2	0	pos_gene:None
CAMSAP3	0	pos_gene:None
PPP5C	1	pos_gene,AHCY
DLX5	0	pos_gene:None
SLC25A19	0	pos_gene:None
EHBP1	0	pos_gene:None
ERAP1	1	pos_gene,IL6
HOXD13	1	pos_gene,TLX3

IL17RD	0	pos_gene:None
COG5	39	pos_gene,CD274,IL23A,STX4
SCN1B	1	pos_gene,CXCR1
SYNM	0	pos_gene:None
LYST	1	pos_gene,NPM1
ITGA6	39	pos_gene,ANPEP,CD36,CD9,SLC22A2
RGS16	0	pos_gene:None
PDZD2	1	pos_gene,RAD21
RPS6KA1	4	pos_gene,BCL2L11,CAV1,NR4A1,TCF15
PAX3	1	pos_gene,HDAC9
COLGALT2	0	pos_gene:None
RALA	2	pos_gene,MYO1G,PTK2B
THEM4	19	pos_gene,FPGS
UBQLN2	4	pos_gene,CEACAM5,CEACAM8,F3
RGS10	0	pos_gene:None
JAKMIP2	0	pos_gene:None
MAP4K4	0	pos_gene:None
COL20A1	2	pos_gene,IL12A,MMP9
HSPH1	0	pos_gene:None
MYH7B	0	pos_gene:None
PEX19	1	pos_gene,MUC1
MAP7	0	pos_gene:None
CROCC	0	pos_gene:None
STXBP2	0	pos_gene:None
RGS5	0	pos_gene:None
CUL4A	4	pos_gene,NFKB2,NFKBIA,SIGLEC9,SOCS1
GJB7	1	pos_gene,SDC1
RNF8	0	pos_gene:None
KCNQ4	0	pos_gene:None
SEMA3A	0	pos_gene:None
GABARAPL1	0	pos_gene:None
PCDHGC3	0	pos_gene:None
CC2D2A	0	pos_gene:None
PCK2	1	pos_gene,RASGRP3
RECQL4	0	pos_gene:None
COMMD1	62	pos_gene,CBFB,NFKB2,NFKBIA,RAD21,SOCS1
KALRN	0	pos_gene:None
PPFIA1	0	pos_gene:None
DRD3	0	pos_gene:None
NDUFAF2	0	pos_gene:None
CLEC11A	1	pos_gene,AMD1
CAMLG	0	pos_gene:None
KPNA1	3	pos_gene,FHIT,MYB,TERT

TPR	2	pos_gene,BIRC2,RAD21
DNAJB6	1	pos_gene,WNK2
GSS	2	pos_gene,DNM2,SAMSN1
RPN2	2	pos_gene,IL2,SET
AHCYL1	1	pos_gene,TKT
SUSD4	1	pos_gene,MAGED2
TRAK2	0	pos_gene:None
TP53BP2	3	pos_gene,BCL2,HDAC9,SAMSN1
ABCC4	2	pos_gene,DHRS9,NOS3
S1PR2	0	pos_gene:None
NCS1	0	pos_gene:None
IL17RC	4	pos_gene,ABCG2,IL2,IL4R,USP18
RARG	1	pos_gene,CEACAM8
PTPRH	0	pos_gene:None
MXI1	0	pos_gene:None
MUC4	1	pos_gene,DHRS9
NAP1L4	0	pos_gene:None
CDH6	0	pos_gene:None
RGS1	0	pos_gene:None
STIM2	0	pos_gene:None
CRBN	2	pos_gene,EWSR1,SALL4
SWAP70	0	pos_gene:None
ANKS1A	0	pos_gene:None
ELOVL5	3	pos_gene,CD33,CEACAM3,PREP
DNM1L	2	pos_gene,AFF1,DCK
TMEM192	2	pos_gene,EPM2A,USP18
PDP1	0	pos_gene:None
GPR161	1	pos_gene,CD53
CDH10	0	pos_gene:None
AIFM1	1	pos_gene,CPOX
SPTLC1	0	pos_gene:None
CCHCR1	0	pos_gene:None
UMPS	2	pos_gene,DNM2,HMBS
TTC3	0	pos_gene:None
NQO2	0	pos_gene:None
NCF2	21	pos_gene,CYBB
GNAT3	3	pos_gene,CXCR4,DIO3,PGR
RNF123	5	pos_gene,CDK5,CXCR2,MAPK1,PARP4,TLR4
ST7	15	pos_gene,ANPEP,ITGA5,PLEKHM1,PREB,PREP,THY1,VANGL1
G6PD	1	pos_gene,AFF1
LGALS8	0	pos_gene:None
MYH6	0	pos_gene:None
RPL7	1	pos_gene,NFKBIB

AIM2	5	pos_gene,BCL11B,CD6,CTCF,POT1,WT1
PRMT2	0	pos_gene:None
SLC7A5	2	pos_gene,PSMC3,RB1
GBP2	0	pos_gene:None
JPH1	1	pos_gene,IL9R
PLS3	0	pos_gene:None
SNRPB	2	pos_gene,ERBB4,PTEN
CREB3	3	pos_gene,JUNB,LRSAM1,MS4A1
GPAA1	0	pos_gene:None
PLEKHF2	2	pos_gene,ERBB4,PTEN
TRAK1	0	pos_gene:None
MTF2	1	pos_gene,EED
TAP2	0	pos_gene:None
DHCR24	0	pos_gene:None
TTBK1	3	pos_gene,CTNNB1,ERBB4,PTEN
DPYSL3	0	pos_gene:None
IARS2	0	pos_gene:None
MAPKAPK3	0	pos_gene:None
S1PR1	2	pos_gene,CD69
EPB42	1	pos_gene,SLC8A1
AFF3	4	pos_gene,MLLT3
ARHGEF11	0	pos_gene:None
OSMR	3	pos_gene,IL6ST,LIF,LIFR
ZFP36	2	pos_gene,RUNX1,TERT
NUP205	1	pos_gene,NR3C1
NEK9	0	pos_gene:None
EXOC5	1	pos_gene,PTK2B
HSPA9	0	pos_gene:None
SERBP1	0	pos_gene:None
EBF1	11	pos_gene,PAX5
CDK7	0	pos_gene:None
CCSAP	0	pos_gene:None
PTGES3	4	pos_gene,DAXX,DNMT1,NR3C2,SPI1
NAGPA	1	pos_gene,CD4
STT3B	1	pos_gene,COX10
B3GAT3	0	pos_gene:None
TMEM33	0	pos_gene:None
PCDHGB4	0	pos_gene:None
NR5A2	1	pos_gene,NSD1
CLCN3	0	pos_gene:None
PCBD1	0	pos_gene:None
SLC2A12	0	pos_gene:None
DGKZ	0	pos_gene:None

GLUL	1	pos_gene,NFKBIB
CLCA1	3	pos_gene,CSPG4,LGALS4,MYO1G
DEK	0	pos_gene:None
NEK2	1	pos_gene,BIRC2
MILR1	3	pos_gene,CD83,FAT3,IL2RG
SRP14	0	pos_gene:None
KIDINS220	1	pos_gene,FAT4
WDR6	1	pos_gene,KDR
ERLIN1	0	pos_gene:None
TK1	1	pos_gene,SLC22A2
HNRNPR	2	pos_gene,BCL11B,CHEK2
CDA	1	pos_gene,DNM2
RAB3C	0	pos_gene:None
ZFPL1	1	pos_gene,VAMP1
SYT6	0	pos_gene:None
ATP2B1	1	pos_gene,NR3C1
COPB2	2	pos_gene,PPA1,RASGRP3
P4HA1	1	pos_gene,CLPB
RGS3	0	pos_gene:None
DNAJC10	4	pos_gene,ANXA5,AZGP1,HSPA4L,LRP1B
COPG2	0	pos_gene:None
SLC25A24	1	pos_gene,CD74
TRIM23	2	pos_gene,CASP9,CRKL
STK38	0	pos_gene:None
PCDHA10	1	pos_gene,TCF15
DOCK4	1	pos_gene,NPM1
NUP54	1	pos_gene,NME1
SRSF11	0	pos_gene:None
UROD	0	pos_gene:None
HCRTR1	1	pos_gene,CNR2
BCHE	2	pos_gene,FN1,MUSK
ATP2A3	0	pos_gene:None
WDR26	2	pos_gene,GH1,LMOD1
COPA	3	pos_gene,CD59,HLA-G,PTPRC
UBAC1	0	pos_gene:None
ARF5	1	pos_gene,AFF1
JKAMP	0	pos_gene:None
SLC25A13	1	pos_gene,IRAK1
GRK6	0	pos_gene:None
DPP6	0	pos_gene:None
PYGL	1	pos_gene,DNM2
MTA3	1	pos_gene,RAD21
PRDX6	2	pos_gene,CYBB,PRDX2

C3orf52	23	pos_gene,CD33,CD9,MRGPRX3,PTCH1,SLC35B2
TULP1	0	pos_gene:None
TMTC3	3	pos_gene,CD72,EGF,LSR
DHX36	1	pos_gene,FUBP1
RHO	0	pos_gene:None
IMPDH2	0	pos_gene:None
ACSL3	0	pos_gene:None
YBX3	0	pos_gene:None
SCN2A	1	pos_gene,TMEM37
MEP1B	8	pos_gene,CD40LG,ERBB2,HSPD1,JAK2,JAK3,PRKCQ,RASA1,STAT3
TPCN2	2	pos_gene,CD83,CXCR1
ACADVL	0	pos_gene:None
LBR	0	pos_gene:None
RAB35	0	pos_gene:None
FOXP4	1	pos_gene,TOP2A
MYO1B	0	pos_gene:None
GPR25	1	pos_gene,CD52
GYPB	0	pos_gene:None
MAB21L2	2	pos_gene,CDH1,JUP
ARL4A	1	pos_gene,BIRC5
LAP3	0	pos_gene:None
RABEP1	1	pos_gene,RBBP8
ABCE1	1	pos_gene,DLD
PKN3	0	pos_gene:None
FAT1	0	pos_gene:None
EIF4G3	1	pos_gene,PSMC3
GCDH	0	pos_gene:None
CHD7	0	pos_gene:None
XPO6	0	pos_gene:None
CTDP1	0	pos_gene:None
SORD	0	pos_gene:None
WIPI2	0	pos_gene:None
PLCB2	3	pos_gene,JAK2,JAK3,PRKCQ
FBXO45	1	pos_gene,KITLG
TRIP11	0	pos_gene:None
SLC6A3	0	pos_gene:None
CAP1	1	pos_gene,ACTBL2
RGS4	0	pos_gene:None
PPP2R5A	0	pos_gene:None
GART	1	pos_gene,AFF1
ANKRD46	1	pos_gene,PLXNA2
HNF4G	0	pos_gene:None
ABCB10	1	pos_gene,NRAS

SLC4A7	4	pos_gene,ATP2A2,CA2,NOS3,SLC9A3R1
NRP2	4	pos_gene,CNTN2,FAT3,FAT4,FLT1
SPECC1L	0	pos_gene:None
GOLGA7	2	pos_gene,MLLT10,MTX1
CCAR2	1	pos_gene,ELL
ADCY3	0	pos_gene:None
IL31RA	4	pos_gene,IL6ST,LIF,LIFR
EIF4A1	0	pos_gene:None
PIK3C2B	0	pos_gene:None
USP19	0	pos_gene:None
TMTC4	2	pos_gene,CD72,EGF
EMC2	1	pos_gene,NME2
SPATA18	0	pos_gene:None
HMG20A	1	pos_gene,CEBPE
PCDH20	5	pos_gene,ABCG2,CD83,FCGR1A,IL2RG,TNFRSF1A
HSF1	0	pos_gene:None
IPO11	1	pos_gene,PRDX2
VGLL3	0	pos_gene:None
TRIM11	0	pos_gene:None
BAG5	0	pos_gene:None
RBM3	0	pos_gene:None
TNS3	2	pos_gene,BCL2L11,CAV1
LPCAT1	1	pos_gene,SI
BAG6	2	pos_gene,E2F1,IL18
LPXN	3	pos_gene,ILK,PICALM,SERPINB2
CSDE1	0	pos_gene:None
TIMM50	1	pos_gene,MAT2A
GCC2	0	pos_gene:None
SOCS6	0	pos_gene:None
COG2	2	pos_gene,CD274,STX4
SEC62	4	pos_gene,CREB1,MAPK14,SRF,XBP1
CDH15	1	pos_gene,F2R
MATK	1	pos_gene,CD9
CEACAM21	1	pos_gene,PIM1
USO1	0	pos_gene:None
DAB1	1	pos_gene,ABCB1
ATP2B4	3	pos_gene,CD22,GP1BA,SI
RASD1	1	pos_gene,CXCR2
WDFY3	0	pos_gene:None
CTPS2	0	pos_gene:None
RPAP2	0	pos_gene:None
ELMO3	0	pos_gene:None
NR1D2	1	pos_gene,CTCF

AGTR1	1	pos_gene,F2R
SNCAIP	0	pos_gene:None
MARK1	0	pos_gene:None
RGS17	10	pos_gene,RBBP8
SSTR2	0	pos_gene:None
HSD11B1	4	pos_gene,CD9,MAP4K1,PTPRT,SLC3A1
NRG2	0	pos_gene:None
ANKRA2	0	pos_gene:None
FKBP4	0	pos_gene:None
TMEM237	2	pos_gene,MRGPRX3,SLC35B2
MTCH1	0	pos_gene:None
POGZ	1	pos_gene,LYL1
EEF1B2	0	pos_gene:None
KIF3C	0	pos_gene:None
DEGS1	1	pos_gene,NRAS
CENPF	0	pos_gene:None
GAD1	0	pos_gene:None
ADRA2A	0	pos_gene:None
RGS14	0	pos_gene:None
ABCA3	4	pos_gene,CD72,EGF,LSR,NRAS
TMEM205	2	pos_gene,CD72,LSR
LEMD3	3	pos_gene,CD72,EGF,LSR
MAPK8IP3	0	pos_gene:None
S1PR5	1	pos_gene,F2R
ATIC	1	pos_gene,AHCY
LMBR1L	2	pos_gene,COX10,PRRT2
CD244	3	pos_gene,CD2,IL18R1,PTPRC
CAPRIN1	0	pos_gene:None
KCNS3	2	pos_gene,PREP,VANGL2
SLC17A2	2	pos_gene,IL4R,TNFRSF17
CD55	21	pos_gene,C2,CD14,CR2
KDM4A	0	pos_gene:None
ATP4A	19	pos_gene,BCL2L1,CCR5,CXCR4,MRC1
SLC35A3	1	pos_gene,PTCH1
SRSF7	0	pos_gene:None
POLDIP2	0	pos_gene:None
GDPD5	1	pos_gene,TNFRSF17
DSE	0	pos_gene:None
IPO7	1	pos_gene,ILK
MED10	0	pos_gene:None
ARPC5	0	pos_gene:None
RCC2	0	pos_gene:None
PTGS2	0	pos_gene:None

S1PR4	1	pos_gene,F2R
BAG1	0	pos_gene:None
PCDHA3	2	pos_gene,IL4R,IL9R
LTV1	0	pos_gene:None
TH	4	pos_gene,CRABP1,ERCC8
STK24	2	pos_gene,CD274,STX4
GMPPA	0	pos_gene:None
BAG3	2	pos_gene,BID,LIF
P4HB	2	pos_gene,GPX4,PREP
SPRED1	4	pos_gene,CEACAM5,MLLT10,PAX5,STAG2
DTL	1	pos_gene,RUNX1T1
RGS20	4	pos_gene,BCL2L1,CCR5,CXCR2,CXCR4
MOV10	1	pos_gene,CBFB
INTS7	0	pos_gene:None
ATF6B	4	pos_gene,CREB1,MAPK14,SRF,XBP1
RAC2	2	pos_gene,CRKL,CYBB
ALPP	0	pos_gene:None
UBXN2A	0	pos_gene:None
PLOD2	2	pos_gene,DNTT,TNFSF13
RBM10	2	pos_gene,EWSR1,SDC1
UBXN2B	0	pos_gene:None
TSHB	6	pos_gene,CGA
TCOF1	2	pos_gene,KDM6A,KMT2C
CDCP1	1	pos_gene,CP
AARS2	0	pos_gene:None
NAT10	2	pos_gene,AATF,RPL34
EDEM3	1	pos_gene,HSPA4L
PCDHGB1	1	pos_gene,PLXNA2
APLP2	0	pos_gene:None
HSD17B12	1	pos_gene,ABCC1
LAPTM4B	0	pos_gene:None
AIMP1	0	pos_gene:None
CERS2	0	pos_gene:None
FARP1	1	pos_gene,TYMS
KLF11	1	pos_gene,RBBP8
GYG1	2	pos_gene,CEACAM6,DNM2
RXFP1	1	pos_gene,PKIA
KCTD3	1	pos_gene,NF1
ARHGAP10	0	pos_gene:None
SMG7	6	pos_gene,ARID1B,FAT4,MLLT6,TET1
RNF19A	0	pos_gene:None
ALDH3A2	2	pos_gene,NME1,TGOLN2
NUP210	0	pos_gene:None

EPB41L2	21	pos_gene,BIRC3,CCND2,MLF1,MLLT1,ZMYM3
DYRK2	0	pos_gene:None
UBR3	3	pos_gene,LGALS4,MYO1G,PLXNA2
FSTL1	13	pos_gene,AMD1,IL12A,WNT5A
PIK3C2A	0	pos_gene:None
GALNT11	10	pos_gene,DHRS9
LIN7C	0	pos_gene:None
SGCB	1	pos_gene,CXCR1
SRSF3	0	pos_gene:None
MTMR6	0	pos_gene:None
JAGN1	1	pos_gene,NRAS
NBEAL2	1	pos_gene,DAZL
CTSD	1	pos_gene,TG
SNX21	1	pos_gene,AMD1
CHD1L	0	pos_gene:None
USP4	1	pos_gene,IRF8
PARK7	2	pos_gene,MLLT6,TRAP1
CDYL	4	pos_gene:EGFR,CREBBP,RAD21,EZH2

Sample 027

Sample-specific Candidate AML genes	Number of specific interactions	In which module of known AML gene
SORBS3	2	pos_gene,CAPN2,FLT3
HGF	24	pos_gene,GP1BA,IL7,IL7R,ITGA5
RGMB	1	pos_gene,CASP3
TUBGCP3	1	pos_gene,S100A8
PCGF1	4	pos_gene,MLLT1,MLLT3,PAX5,SUZ12
SUFU	1	pos_gene,BCL2
PSMD3	3	pos_gene,CCND2,MLF1,PSMD9
RAB1A	1	pos_gene,BRAF
KIF22	0	pos_gene:None
BRPF3	1	pos_gene,HOXA9
RPL9	2	pos_gene,CTNNA1,RPL34
MECP2	2	pos_gene,NSD1,VEGFA
KHDRBS1	0	pos_gene:None
ANKRD1	0	pos_gene:None
SOX9	0	pos_gene:None
ECSIT	22	pos_gene,CYP24A1,ECT2L,FAS,NOS3
CRTC2	0	pos_gene:None
AP1M1	5	pos_gene,CD86,CTLA4,FLI1,SELPLG,VANGL2
FBXO7	8	pos_gene,ASXL1,ASXL2,BID,CCNY,GFAP,KRAS,NFKB1,SLC39A2
KLHL20	0	pos_gene:None

POLE3	0	pos_gene:None
NIPBL	1	pos_gene,MLLT3
STAM2	2	pos_gene,JAK2,JAK3
EIF3L	0	pos_gene:None
FOXC2	4	pos_gene,BCL11B,BCOR,RARA,SALL4
APOA1	22	pos_gene,APP,CD40LG,CP,HBA2,XPO1
FOXA2	2	pos_gene,BCOR,CTCF
POLR1C	4	pos_gene,CNTN2,MSH6,NFKBIB
REV1	1	pos_gene,MSH2
FBXO25	0	pos_gene:None
GAS7	0	pos_gene:None
COL4A1	4	pos_gene,FAT3,FCGR3A,SNCA,THBS1
PHF1	0	pos_gene:None
LIG3	0	pos_gene:None
PNKP	0	pos_gene:None
TREX1	0	pos_gene:None
FOXK2	2	pos_gene,KDM6A,KMT2C
CCDC88A	0	pos_gene:None
RAB8A	3	pos_gene,CD86,CTLA4
FOXD3	1	pos_gene,BTG1
WASF2	1	pos_gene,BTK
TNKS1BP1	1	pos_gene,UIMC1

Sample 168

**Sample-specific Candidate AML genes**	**Number of specific interactions**	**In which module of known AML gene**
TPGS2	4	pos_gene,ACTBL2,CNOT3,HSD3B1,SCYL1
CCNE1	6	pos_gene,CDK4,CDK6,CKS1B,RB1,SF3B1,TERT
TACC1	0	pos_gene:None
CPSF3	1	pos_gene,ANKRD49
SCLT1	0	pos_gene:None
DCAF7	1	pos_gene,SETBP1
MYL12A	0	pos_gene:None
BDNF	1	pos_gene,NCAM1
RPL37A	0	pos_gene:None
NAP1L1	1	pos_gene,MAGED2
HNF1B	1	pos_gene,HNF1A
TNNI2	1	pos_gene,TLX3
SYT17	1	pos_gene,PNMA3
KCNMB2	0	pos_gene:None
MYL9	0	pos_gene:None

TDRD7	0	pos_gene:None
BMP7	1	pos_gene,BMP6
WIPF1	0	pos_gene:None
SYCE1	0	pos_gene:None
VAMP8	0	pos_gene:None
SKA1	0	pos_gene:None
TXK	0	pos_gene:None
MZT2B	0	pos_gene:None
TUBG2	0	pos_gene:None
SETD7	19	pos_gene,KLF4
CD28	23	pos_gene,CBL,CD2,CD4,CD79A,CD86,CTLA4,FCGR3A,IFNAR1,JAK2,LCK,RASA1
AQR	1	pos_gene,SETD2
RBM45	0	pos_gene:None
SNX1	1	pos_gene,EPM2A
PRMT6	1	pos_gene,HSP90AA1
MED6	0	pos_gene:None
LEPR	0	pos_gene:None
MZT1	0	pos_gene:None
POLK	0	pos_gene:None
POC1A	2	pos_gene,CNOT3,SCYL1
RBL2	1	pos_gene,E2F1
THOC2	1	pos_gene,ZMYM3
UBTF	1	pos_gene,ZMYM2
ACTL6B	3	pos_gene,ARID1A,ARID1B,NEUROD1
NRBF2	1	pos_gene,DCT
TXNDC12	1	pos_gene,FPGS
TSG101	2	pos_gene,AATF,MUSK
CSTF2	0	pos_gene:None
SKA3	0	pos_gene:None
CCNC	1	pos_gene,CKS1B
SDCBP	6	pos_gene,CD5,CD6,SDC1
GNB3	1	pos_gene,GNAI1
PBRM1	4	pos_gene,ARID1A,ARID1B,CDX2,NEUROD1
TCF7	1	pos_gene,FHIT
SS18L1	0	pos_gene:None
STAT4	53	pos_gene,IL2RA,IL2RB,IRF4,TBX21
MEN1	4	pos_gene,FANCA,MYB,PSIP1,WT1
LCA5	1	pos_gene,PRAME
CD79B	3	pos_gene,CD19,CR2,PTPRC
EXTL3	1	pos_gene,VANGL2
ITK	0	pos_gene:None
PRLR	20	pos_gene,GH1,GHR

FOXM1	0	pos_gene:None
LRRCC1	0	pos_gene:None
MED11	0	pos_gene:None
CFHR1	2	pos_gene,C3
MKNK1	1	pos_gene,JAK1
CCNB1	1	pos_gene,RB1
RAB3IP	1	pos_gene,NF1
SH2B2	0	pos_gene:None
TEKT1	2	pos_gene,EFHC1,IKZF1
CC2D1A	0	pos_gene:None
STX7	1	pos_gene,CD70
LIMD1	0	pos_gene:None
SMARCC2	6	pos_gene,ARID1A,ARID1B,BCL3,FLI1,NEUROD1,TBL1XR1
KNSTRN	0	pos_gene:None
MMS22L	0	pos_gene:None
CYCS	1	pos_gene,PRDX2
LMNB2	0	pos_gene:None
EIF4EBP1	0	pos_gene:None
TLE4	0	pos_gene:None
SLAIN2	2	pos_gene,EED,SUZ12
QRFPR	0	pos_gene:None
LIMS1	2	pos_gene,ILK,TKT
SIN3B	1	pos_gene,CDCA7L
SMARCD2	4	pos_gene,ARID1A,ARID1B,FLI1,NEUROD1
NPAT	0	pos_gene:None
EPN3	3	pos_gene,CASP2,ITGA4,TFRC
MED21	1	pos_gene,CKS1B
TOM1L1	0	pos_gene:None
FAM83D	0	pos_gene:None

Sample 270

Sample-specific Candidate AML genes	Number of specific interactions	In which module of known AML gene
LINGO2	0	pos_gene:None
AIP	1	pos_gene,PREP
FAM110A	0	pos_gene:None
GAS8	2	pos_gene,BRCA1,CBFA2T3
KRT9	0	pos_gene:None
CASR	0	pos_gene:None
ADORA2A	2	pos_gene,CNR1,PRKCQ
SEC24C	0	pos_gene:None
PIK3C3	1	pos_gene,USP18

COX5B	1	pos_gene,CASP2
OPA3	0	pos_gene:None
BCKDHA	2	pos_gene,DLD,PPM1K
CACNA1H	1	pos_gene,CAV1
ARC	0	pos_gene:None
IL16	0	pos_gene:None
RELB	6	pos_gene,BCL3,BIRC3,CLTC,IL1B,IRF4,NFKB2
JMJD1C	0	pos_gene:None
HMOX1	2	pos_gene,LSR,RNF130
DYNLT1	2	pos_gene,CASP8,TCF15
DYNLT3	0	pos_gene:None
IL17A	6	pos_gene,IL2
ATXN7L3	4	pos_gene,BRCA2,MED12,TCF3,TRRAP
CCP110	0	pos_gene:None
DKK3	0	pos_gene:None
RTN4IP1	0	pos_gene:None
CIAO1	2	pos_gene,PCNA,WT1
C1D	0	pos_gene:None
HEY2	6	pos_gene,ETV6,FLI1,MAPK8,SYK,TP73,ZBTB16
DNAJC5	7	pos_gene,CD2,EDN1,PPARG,STX4,SYNJ1,VAMP1,VAMP2
EIF2S3	4	pos_gene,CASP9,DIO3,MAPK3,RASA1
CEP95	0	pos_gene:None
DET1	0	pos_gene:None
DYNLRB1	1	pos_gene,ELL
AP4M1	0	pos_gene:None
ROCK2	1	pos_gene,MBP
RBFOX2	0	pos_gene:None
DVL1	1	pos_gene,F2R
DDX42	2	pos_gene,CASP8,HSPA4
TUBB4A	4	pos_gene,LRP1,NRAS,PLEKHM1,TNFRSF1A
GSPT2	1	pos_gene,EGFR
HAUS1	0	pos_gene:None
IGF2BP3	0	pos_gene:None
GNAZ	3	pos_gene,BIRC2,FOS,HSP90AA1
HAUS5	0	pos_gene:None
TAF13	1	pos_gene,CTCF

Sample 472

Sample-specific Candidate AML genes	Number of specific interactions	In which module of known AML gene
SV2A	0	pos_gene:None
CLTA	3	pos_gene,ITGA4,PICALM,SYNJ1

CRTAC1	2	pos_gene,ANXA5,AZGP1
MYRIP	0	pos_gene:None
HCRTR2	0	pos_gene:None
ITGA3	2	pos_gene,CD38,CSPG4
TRADD	0	pos_gene:None
SMOC2	1	pos_gene,CD40LG
SPEN	2	pos_gene,ICOS,RBBP8
LAMA3	1	pos_gene,GH1
NDUFS5	1	pos_gene,PHB2
NAV2	8	pos_gene,CDH1,MKI67
ANKRD2	0	pos_gene:None
MEAF6	1	pos_gene,TRRAP
EID3	0	pos_gene:None
MED12L	14	pos_gene,CKS1B
TGFBR3	0	pos_gene:None
C1QTNF9	1	pos_gene,IL12A
PCSK2	0	pos_gene:None
ZFYVE27	1	pos_gene,SLC39A2
MSH5	0	pos_gene:None
OLFM3	0	pos_gene:None
MYH14	0	pos_gene:None
KCNC2	0	pos_gene:None
SUMF1	0	pos_gene:None
CYP1B1	0	pos_gene:None
NDRG1	0	pos_gene:None
SUDS3	0	pos_gene:None
ADAMTS2	0	pos_gene:None
JAM2	0	pos_gene:None
COL7A1	2	pos_gene,CSF1R,GH1
VWF	19	pos_gene,GP1BA,IL7,PDGFB,THBS1,TNFSF10,TNFSF13
PELI1	18	pos_gene,IRAK1,MYD88,TLR7
ITIH2	1	pos_gene,LPL
GLB1L2	0	pos_gene:None
PKN2	0	pos_gene:None
EYA2	1	pos_gene,MRC2
PKP3	1	pos_gene,CTNNA1
ZFPM2	20	pos_gene,EZH2,GATA1
ADAM12	5	pos_gene,EGF,PACSIN3
COLEC12	0	pos_gene:None
KCNJ2	0	pos_gene:None
MYO1E	0	pos_gene:None
TONSL	0	pos_gene:None
C8B	5	pos_gene,C5,CD59

MYO15A	0	pos_gene:None
ORC1	1	pos_gene,PHF6
COCH	0	pos_gene:None
GABRA2	0	pos_gene:None
EYS	1	pos_gene,EGFR
LMX1B	2	pos_gene,MDM2,PBX1
GPC6	7	pos_gene,DLX4
SPAG16	0	pos_gene:None
UHRF2	4	pos_gene,CHD2,DNMT1,DNMT3A,SPI1
SPATA13	0	pos_gene:None
NBR1	0	pos_gene:None
SEMA5A	0	pos_gene:None
CLIP1	0	pos_gene:None
COL2A1	6	pos_gene,ANXA5,FAT3,FGFR2,MBP,PDGFB
TLN2	1	pos_gene,ITGB2
NAALADL2	0	pos_gene:None
CDH3	0	pos_gene:None
RIT2	0	pos_gene:None
SCN4B	0	pos_gene:None
GABRG1	0	pos_gene:None
GABRA6	0	pos_gene:None
GNG10	13	pos_gene,CCR1,GNAI1
FGG	22	pos_gene,F2,ICAM1,ITGAM
C8A	4	pos_gene,C5,CD59
PLCB4	0	pos_gene:None
HDGF	0	pos_gene:None
KIRREL3	0	pos_gene:None
TEAD4	0	pos_gene:None
GABRG3	0	pos_gene:None
HCFC1	3	pos_gene:HNF4A,PPARGC1A,JUN

Sample 546

**Sample-specific Candidate AML genes**	**Number of specific interactions**	**In which module of known AML gene**
EMILIN2	0	pos_gene:None
ZNF638	0	pos_gene:None
FLT4	2	pos_gene,GNAS,KDR
HR	1	pos_gene,TBL1XR1
HOXC8	1	pos_gene,ERBB4
STARD7	0	pos_gene:None
NEK7	47	pos_gene,ACTBL2,CNOT3,DHRS9,HSD3B1,SCYL1
DNMT3B	1	pos_gene,DNMT3A

F11R	4	pos_gene,CD69,CD79A,MS4A3,TYMS
DIAPH1	0	pos_gene:None
LOXL2	1	pos_gene,GH1
RPL23	3	pos_gene,DIO3,RASA1,RPL5
STOM	3	pos_gene,MRGPRX3,RTKN,TNFRSF10B
VPS52	1	pos_gene,WTAP
U2SURP	0	pos_gene:None
INCA1	0	pos_gene:None
TOMM40	6	pos_gene,BAX,COX10,DLD,HSPB1,PHB2,RASGRP3
THRAP3	0	pos_gene:None
PXDN	0	pos_gene:None
IQGAP2	0	pos_gene:None
CYP2S1	0	pos_gene:None
MRRF	0	pos_gene:None
CALCOCO2	1	pos_gene,C3
TSC22D1	1	pos_gene,MAGED2
RANBP9	1	pos_gene,HMBS
MAPK11	0	pos_gene:None
STC2	1	pos_gene,GH1
SDHC	0	pos_gene:None
PA2G4	0	pos_gene:None
NTNG2	0	pos_gene:None
RAB11FIP5	1	pos_gene,CXCR2
HSPA1B	0	pos_gene:None
DRD1	0	pos_gene:None
ACACA	0	pos_gene:None
ACAD9	0	pos_gene:None
KLHL1	0	pos_gene:None
SORL1	8	pos_gene,IL6,IL6R,IL6ST,LIFR,NCAM1,RHOH,SDC1
TRIP6	2	pos_gene,HOXA9,SET
ITGA2	2	pos_gene,CD53,CD9
OGG1	1	pos_gene,ERCC8
MTCH2	0	pos_gene:None
AGAP1	0	pos_gene:None
ATP6V0A4	1	pos_gene,TNFRSF1B
PTBP1	24	pos_gene,DNTT,HMBS,HSD3B1,PKIA,RORA,TYMS,ZMYM2
PEBP1	1	pos_gene,ICOS
PROS1	11	pos_gene,CP,CTSG,F2,F2RL1,F3,F5,THBD
TAF15	2	pos_gene,FLI1,HBG2
DCUN1D1	2	pos_gene,EDN1,HMBS
COLEC11	1	pos_gene,C2
SDC4	7	pos_gene,CD36,CD40LG,IL7,IL7R,NCAM1,TNFSF13,VEGFA
SRSF10	1	pos_gene,TYMS

RPRD2	17	pos_gene,HSPA8,XPO1
THOC5	1	pos_gene,MYO1G
TNNT2	1	pos_gene,NOS3
ADRA1B	0	pos_gene:None
CPE	0	pos_gene:None
KCNH2	1	pos_gene,RHOH
NTN4	2	pos_gene,RASA1,RPL5
MICU2	0	pos_gene:None
VTI1A	0	pos_gene:None
EOGT	5	pos_gene,APP
KMT2E	0	pos_gene:None

Sample 629

Sample-specific Candidate AML genes	Number of specific interactions	In which module of known AML gene
LGALS3BP	6	pos_gene,CD14,MRC2,PDGFB
RPL11	6	pos_gene,HBG2,NFKBIB,RPL10,RPL19,RPL5,TP73
METTL14	0	pos_gene:None
BCAP31	3	pos_gene,CASP1,RET,VAMP1
RPL7A	1	pos_gene,HBG2
KLHL2	0	pos_gene:None
TCF20	0	pos_gene:None
OTC	0	pos_gene:None
KCNA1	0	pos_gene:None
NXF1	1	pos_gene,AATF
EDA	5	pos_gene,IL2RG,IL9R,LSR,PIK3R1
UNC93B1	8	pos_gene,ERVW-1,GGH,MAGED2,PRRT2,SIGLEC9,TLR7
GIT1	0	pos_gene:None
MRPS15	0	pos_gene:None
NCSTN	0	pos_gene:None
OXCT1	1	pos_gene,DCK
USP10	1	pos_gene,TBX21
MICU1	1	pos_gene,FBXW7
NDUFA8	0	pos_gene:None
SNIP1	0	pos_gene:None
GTF3C4	0	pos_gene:None
IFT20	0	pos_gene:None
MAP2K2	6	pos_gene,CASP9,CD70,CDH1,MAP4K1,SERPINB2,TKT
BECN1	1	pos_gene,MAPKAPK2
ANTXR2	0	pos_gene:None
RPS4X	0	pos_gene:None
LDB3	1	pos_gene,ERBB4

FGB	5	pos_gene,ICAM1,MAP2K1,NME2,PTPN11,WNK2
SMCHD1	1	pos_gene,APEX1
KTN1	1	pos_gene,CD68
PCBP1	3	pos_gene,BIRC3,CHEK2,PRDX2
CKMT1A	0	pos_gene:None
CDC6	0	pos_gene:None
IL20RA	0	pos_gene:None
GTF3C3	2	pos_gene,EZH2,SMC1A
GTF3C5	0	pos_gene:None
CPT1A	0	pos_gene:None
WDR77	0	pos_gene:None
PKNOX2	1	pos_gene,CD1A
TANK	7	pos_gene,BIRC2,CASP8,CD40,CNOT3,HSPA5,MYD88,PFDN4
MED20	0	pos_gene:None
PTPN3	3	pos_gene,ERVW-1,VANGL1,VANGL2
NFIB	0	pos_gene:None
CALML5	0	pos_gene:None
ATPAF1	1	pos_gene,DIABLO
LAMTOR2	0	pos_gene:None
NUP155	0	pos_gene:None
PPP1R9B	1	pos_gene,GHR
RAB14	1	pos_gene,NISCH
PRPF19	0	pos_gene:None
RPL31	5	pos_gene,CRKL,RPL10,RPL19,RPL34,RPL5
TRIM54	8	pos_gene,CDKN1A,HBA2,HBB,HBG2,PCNA,TGOLN2,USP18
ACO2	3	pos_gene,IDH1,IDH2,PRDX2

Sample 773

**Sample-specific Candidate AML genes**	**Number of specific interactions**	**In which module of known AML gene**
CRX	1	pos_gene,TLX3
ESRRB	0	pos_gene:None
RRBP1	0	pos_gene:None
PITX2	0	pos_gene:None
USP2	0	pos_gene:None
FOSB	0	pos_gene:None
ARHGAP24	0	pos_gene:None
SPARC	5	pos_gene,CD36,CTSG,ELANE,FAT3,THBS1
GORASP1	0	pos_gene:None
ITGA8	0	pos_gene:None
CLDN19	2	pos_gene,C2,CD68
AGK	1	pos_gene,SI

UCHL1	5	pos_gene,CDK5,MPL,NCAM1,TNFRSF1A,WBP1L
IMMP1L	2	pos_gene,MPL,MRC2
PPP1R9A	1	pos_gene,DIO3
GPRASP1	0	pos_gene:None
CUL4B	3	pos_gene,CBFB,EGFR,ERCC8
PINK1	2	pos_gene,CASP1,MPL
VPS18	0	pos_gene:None
ELP2	0	pos_gene:None
SLC9A3	1	pos_gene,TG
MGST3	3	pos_gene,CEACAM3,F3,MRGPRX3
UCHL5	4	pos_gene,HBA2,MLLT6,RAN,SET
RABGEF1	0	pos_gene:None
PRMT5	0	pos_gene:None
TRIM32	0	pos_gene:None
SOCS3	1	pos_gene,CTLA4
GJB1	5	pos_gene,CD53,MAML2,MRGPRX3,MUC1,RASGRP3
PRKAB1	5	pos_gene,HSPB1,JUN,MAP4K1,ST13,TNFRSF1A
SPTBN4	0	pos_gene:None
TLK2	3	pos_gene,HLF,IL23A
RAP1GAP	1	pos_gene,RASGRP3
PES1	0	pos_gene:None
RALY	3	pos_gene,CD6,FOXP3,PHF6
ADD3	0	pos_gene:None
TACC2	0	pos_gene:None
COX5A	1	pos_gene,SLC8A1
THSD7A	3	pos_gene,CD33
MYOF	0	pos_gene:None
DNALI1	1	pos_gene,HPS4
TFDP2	0	pos_gene:None
KATNAL1	0	pos_gene:None
SYNJ2BP	1	pos_gene,CD14
APPBP2	0	pos_gene:None
NUP153	8	pos_gene,BIRC3,CDK4,EED,MAP2K1,MLLT6,RAD21,RAN,WTAP
PAX6	3	pos_gene,CDX2,HOXA9,PAX5
