# Supplementary material for: CGMega: explainable graph neural network framework with attention mechanisms for cancer gene module dissection
Source: Nat Commun. 2024 Jul 17;15:5997. doi: 10.1038/s41467-024-50426-6 (PMC11252405; doi:10.1038/s41467-024-50426-6)
Supplement: Supplementary file 3 — Reporting Summary [file 41467_2024_50426_MOESM3_ESM.pdf]

nature portfolio

Corresponding author(s): Xiaochen Bo

Last updated by author(s): Feb 14, 2024

Reporting Summary

Nature Portfolio wishes to improve the reproducibility of the work that we publish. This form provides structure for consistency and transparency in reporting. For further information on Nature Portfolio policies, see our Editorial Policies and the Editorial Policy Checklist.

Statistics

For all statistical analyses, confirm that the following items are present in the figure legend, table legend, main text, or Methods section.

n/a	Confirmed	
☐	☒	The exact sample size (n) for each experimental group/condition, given as a discrete number and unit of measurement
☐	☒	A statement on whether measurements were taken from distinct samples or whether the same sample was measured repeatedly
☐	☒	The statistical test(s) used AND whether they are one- or two-sided<br>*Only common tests should be described solely by name; describe more complex techniques in the Methods section.*
☒	☐	A description of all covariates tested
☒	☐	A description of any assumptions or corrections, such as tests of normality and adjustment for multiple comparisons
☐	☒	A full description of the statistical parameters including central tendency (e.g. means) or other basic estimates (e.g. regression coefficient) AND variation (e.g. standard deviation) or associated estimates of uncertainty (e.g. confidence intervals)
☐	☒	For null hypothesis testing, the test statistic (e.g. F, t, r) with confidence intervals, effect sizes, degrees of freedom and P value noted<br>*Give P values as exact values whenever suitable.*
☒	☐	For Bayesian analysis, information on the choice of priors and Markov chain Monte Carlo settings
☒	☐	For hierarchical and complex designs, identification of the appropriate level for tests and full reporting of outcomes
☐	☒	Estimates of effect sizes (e.g. Cohen's d, Pearson's r), indicating how they were calculated

Our web collection on statistics for biologists contains articles on many of the points above.

Software and code

Policy information about availability of computer code

Data collection	In this study, we collected data from public databases. All datasets are downloaded manually from the corresponding URL. No software was used for data collection.
Data analysis	We extracted high-confidence (level ≥ 0.95) cancer genes using DigSEE software.<br>We used NeoLoopFinder software on raw Hi-C contact maps to remove the complex structural variants.<br>We used deepTools software to calculate the densities for ATAC-seq, H3K4me3, H3K27ac, and CTCF ChIP-seq.<br>We calculated the IC 50 and HillSlope values using GraphPad Prism.<br>We constructed CGMega with Pytorch, and the tutorial and source codes for both model training and interpretation are available at Zenodo repository (https://zenodo.org/records/10086978).

For manuscripts utilizing custom algorithms or software that are central to the research but not yet described in published literature, software must be made available to editors and reviewers. We strongly encourage code deposition in a community repository (e.g. GitHub). See the Nature Portfolio guidelines for submitting code & software for further information.

Data

Policy information about availability of data

All manuscripts must include a data availability statement. This statement should provide the following information, where applicable:

- Accession codes, unique identifiers, or web links for publicly available datasets
- A description of any restrictions on data availability
- For clinical datasets or third party data, please ensure that the statement adheres to our policy

ATAC-seq, H3K4me3, H3K27ac, and CTCF ChIP-seq data were obtained from the ENCODE project (https://www.encodeproject.org/) for MCF7 and K562 cell lines, and from Gene Expression Omnibus (GEO, https://www.ncbi.nlm.nih.gov/geo/) with accession number GSE152136 for AML patients.
The SNVs and CNVs information were retrieved from The Cancer Genome Atlas (TCGA) project using the corresponding data generated from the closest tumour types of breast cancer and myeloid leukemia.
PPI network was obtained from the ConsensusPathDB (CPDB) database (http://cpdb.molgen.mpg.de/).
Hi-C data were obtained from GEO with accession number GSE66733 for the MCF7 cell line, GSE63525 for the K562 cell line, and GSE152136 for AML patients.
External datasets for CGMega performance were described in Supplementary Data 1. Human genome annotation (GRCh38) was used in this study.

Research involving human participants, their data, or biological material

Policy information about studies with human participants or human data. See also policy information about sex, gender (identity/presentation), and sexual orientation and race, ethnicity and racism.

Reporting on sex and gender	Sex and gender were not considered in this work.
Reporting on race, ethnicity, or other socially relevant groupings	Race, ethnicity and other socially relevant groupings were not considered in this work.
Population characteristics	No human research participants were involved in this work.
Recruitment	No recruitment.
Ethics oversight	No organization.

Note that full information on the approval of the study protocol must also be provided in the manuscript.

Field-specific reporting

Please select the one below that is the best fit for your research. If you are not sure, read the appropriate sections before making your selection.

☒ Life sciences ☐ Behavioural & social sciences ☐ Ecological, evolutionary & environmental sciences

For a reference copy of the document with all sections, see nature.com/documents/nr-reporting-summary-flat.pdf

Life sciences study design

All studies must disclose on these points even when the disclosure is negative.

Sample size	Sample sizes were determined based on previous experiments, we repeated drug (or drugs combination) treatment experiments three times under 8 inhibitor dose points (sample size = 8). This sample size was selected to be sufficient to perform paired t-test and draw a statistical conclusion.
Data exclusions	No data exclusions.
Replication	For inhibitor treatment and western blot experiments, independent experiments were repeated three times for each time point. All attempts at replications are successful.
Randomization	Sample allocation was not performed.
Blinding	Blinding was not performed to investigators as MCF-7 cell line with olaparib or with olaparib/RKI-1447 combination and those data are quantitative and would not easily subject to operator bias.

Reporting for specific materials, systems and methods

We require information from authors about some types of materials, experimental systems and methods used in many studies. Here, indicate whether each material, system or method listed is relevant to your study. If you are not sure if a list item applies to your research, read the appropriate section before selecting a response.

Materials & experimental systems

n/a	Involved in the study
[ ]	[x] Antibodies
[ ]	[x] Eukaryotic cell lines
[x]	[ ] Palaeontology and archaeology
[x]	[ ] Animals and other organisms
[x]	[ ] Clinical data
[x]	[ ] Dual use research of concern
[x]	[ ] Plants

Methods

n/a	Involved in the study
[x]	[ ] ChIP-seq
[x]	[ ] Flow cytometry
[x]	[ ] MRI-based neuroimaging

Antibodies

Antibodies used	1. ROCK2 (middle) Polyclonal antibody: SANTA CRUZ Biotechnology, the United States; Cat No 21645-1-AP; clone name: KIAA0619, p164 ROCK2, Rho kinase 2.<br>2. BRCA2 Polyclonal antibody: Abcam, Cambridge, United Kingdom; Cat No 29450-1-AP; clone name: FACD, FANCD1.<br>3. GAPDH: Proteintech, the United States.
Validation	1. ROCK2: HT-1080 cells were subjected to SDS PAGE followed by western blot with 21645-1-AP (ROCK2(middle) antibody) at dilution of 1:1000 incubated at room temperature for 1.5 hours.<br>2. BRCA2: Various lysates were subjected to SDS PAGE followed by western blot with 29450-1-AP (BRCA2 antibody) at dilution of 1:4000 incubated at room temperature for 1.5 hours.<br>3. GAPDH: Western blot of Hela cell with anti-GAPDH (60004-1-Ig) at various dilutions.

Eukaryotic cell lines

Policy information about cell lines and Sex and Gender in Research

Cell line source(s)	MCF-7 human breast cancer cell line was purchased from Beijing YangGuang Biotechnology Co.
Authentication	The cell line was identified as a human cell line, and the results of STR typing were similar to those of MCF-7 Normal.
Mycoplasma contamination	No multiple alleles and no cross contamination were found in this cell line.
Commonly misidentified lines (See ICLAC register)	No cell lines used in this study are found in the database of commonly misidentified cell lines based on short tandem repeats (STR)

Plants

Seed stocks	none.
Novel plant genotypes	none.
Authentication	none.
